# A machine learning method for calculating highly localized protein stabilities

**DOI:** 10.1038/s41467-026-75590-9

**Published:** 2026-09-01

**Authors:** Chenlin Lu, Kyle C. Weber, Savannah K. McBride, Andrew Reckers, Anum Glasgow

**Affiliations:** Department of Biochemistry and Molecular Biophysics, Columbia University, New York, NY USA

**Keywords:** Proteins, Mass spectrometry, Molecular conformation

## Abstract

The residue-level free energy of opening (∆G_op_) is the thermodynamic descriptor of localized protein stability, providing valuable information about the protein ensemble at physiologically relevant timescales and conditions. PFNet instantly determines ∆G_op_ for arbitrarily large proteins and complexes from conventional peptide-level hydrogen exchange-mass spectrometry (HX-MS) datasets. It unlocks the full potential of HX-MS, democratizing the method and establishing quantitative, scalable and accessible analysis.

## Abstract (web summary)

PFNet is a machine learning model that infers residue-level protein stabilities from conventional hydrogen exchange-mass spectrometry data, transforming a widely used structural biology technique into a scalable tool for quantitative protein ensemble analysis.

## Introduction

A complete understanding of protein function requires moving beyond static snapshots to the conformational ensembles that drive biological activity. Mutations, post-translational modifications, and molecular interactions regulate protein functions by reweighting their ensembles. Thus, probing shifts in the conformational ensembles of proteins across physiologically relevant timescales and conditions is essential for truly understanding the molecular basis for protein function and enabling the rational design of sophisticated functional proteins. However, a fundamental technological barrier has hindered this pursuit: the lack of tools to quantitatively monitor these energetic changes at high resolution.

Hydrogen exchange-mass spectrometry (HX-MS) is a powerful technique that monitors the ensemble-averaged kinetics of hydrogen isotope exchange in the protein backbone, thus reporting on localized protein stabilities. The data are typically interpreted qualitatively via plots that show relative differences in peptide deuterium uptake among protein functional states. However, in theory, HX-MS data contain all the information necessary to derive individual hydrogen exchange rates (k_ex_) for most backbone amide groups in a protein, which can be used to calculate residue-level free energies of opening (∆G_op_)^1–3^: the thermodynamic descriptor of the localized stability^4^. Despite recent advances^3–5^, a method that simultaneously achieves accuracy, efficiency, scalability, and user-friendliness remains elusive, thereby impeding the full potential of ∆G_op_ as a scalable tool for protein ensemble analysis.

**Fig. 1:**
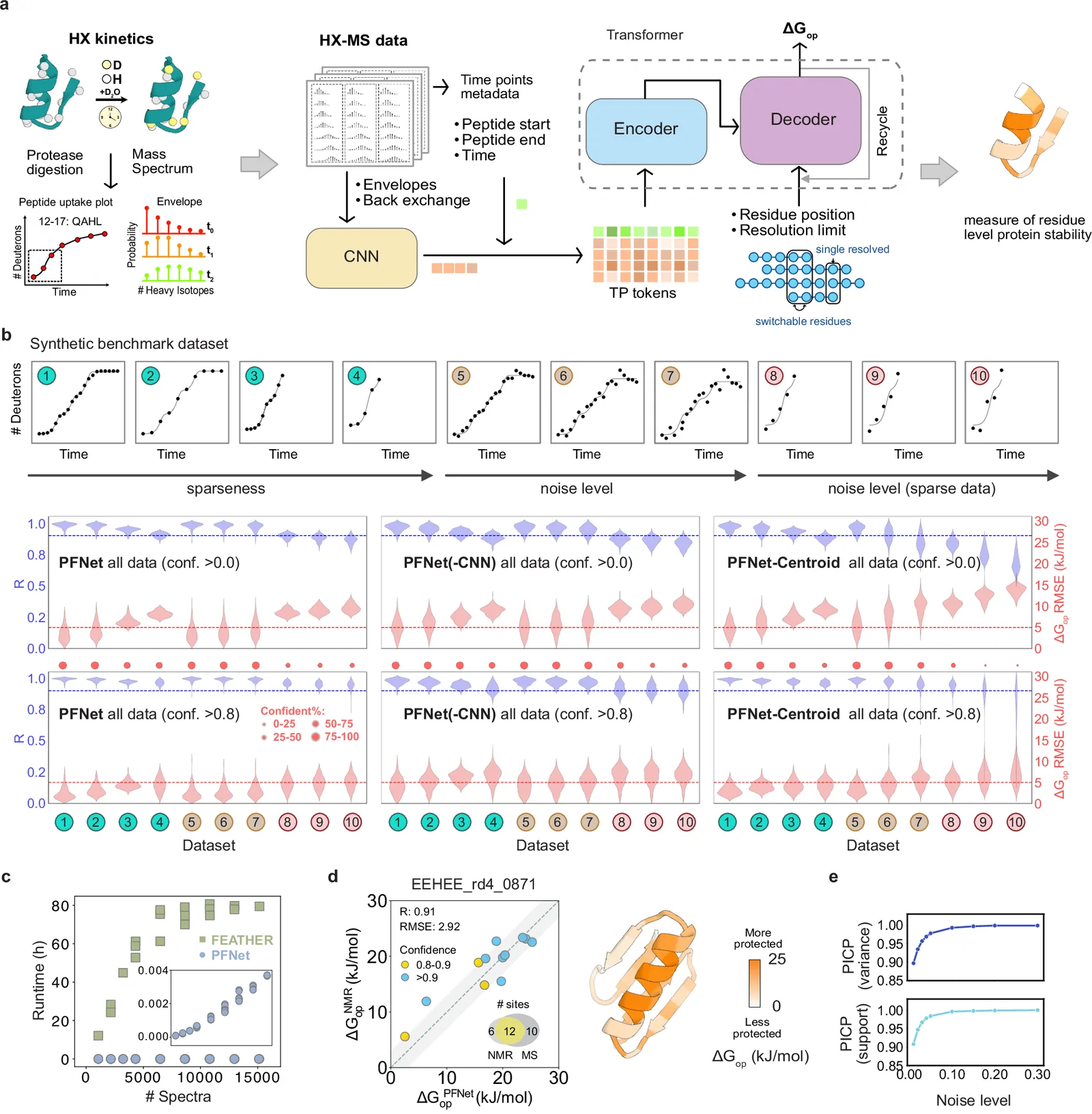
PFNet architecture and benchmarking. **a** Schematic representation of the PFNet architecture and data flow. **b** Benchmarking of PFNet, PFNet(–CNN), and PFNet-Centroid on synthetic datasets with varying time windows, sparseness, and noise levels (Supplementary Table 1), illustrated by a schematic representation of a peptide uptake plot. The Pearson correlation coefficient (R) and root-mean-squared error (RMSE) distributions were calculated with and without applying a confidence threshold of 0.8, respectively. **c** Runtime comparison between PFNet and FEATHER on the *perfect* synthetic dataset (Supplementary Table 1). **d** Experimental benchmarking on the EEHEE_rd4_0871 dataset. The error band indicates ± RMSE along the diagonal between the PFNet-derived values and the HX-NMR measurements. The Venn diagram shows the number of single-residue sites resolved by each technique. **e** PICP analysis estimating the equivalent noise levels of the EEHEE_rd4_0871 HX-MS dataset corresponding to the synthetic benchmarks.

Therefore, in this work, we developed PFNet, a machine learning framework that formulates the inference of residue-level ∆G_op_ from conventional peptide-level HX-MS datasets as a scalable inverse problem. PFNet takes isotopic mass envelopes from HX-MS experiments on proteins as inputs, encodes them as timepoint token representations, and outputs residue-level ${\log }_{10}{k}_{{{{\rm{ex}}}}}$ at the highest spatial resolution that the data afford, with confidence scores (Fig. 1a). We trained PFNet on more than two million proteins with 1000–10,000 synthetic HX-MS isotopic envelopes each. The training data mimicked real HX-MS data by including varied timepoints, noise, peptide coverage, and D-to-H back-exchange, which occurs in the chromatography steps of the experiment. Because most published HX-MS datasets only include MS centroids rather than full isotopic mass envelopes, we also developed the alternative model PFNet-Centroid. Notably, PFNet does not require protein structures, evolutionary information, or other auxiliary biophysical inputs, which demonstrates that residue identities and exchange kinetics in the Linderstrøm-Lang framework^6,7^ are sufficient for deriving residue-level ∆G_op_ from HX-MS data.

## Results

We first developed a synthetic HX-MS benchmark dataset to test PFNet performance and runtime without the confounding factors present in experimental datasets (Fig. 1b). The benchmark set comprises 1000 synthetic proteins that closely resemble experimental observations in terms of quality, noise, redundancy, and spatiotemporal sparseness (Supplementary Table 1). We used two metrics to assess model performance: the Pearson correlation of the ${\log }_{10}{k}_{{{{\rm{ex}}}}}$ predictions to the ground truth (R); and the root-mean-squared error (RMSE).

Testing PFNet on the benchmark data revealed that it achieves higher accuracy and greater robustness to noise than FEATHER (Fig. 1b, Supplementary Figs. 1–3), a state-of-the-art Bayesian method for high-resolution ∆G_op_ determination^3^, but with four to five orders of magnitude less analysis time (Fig. 1c, from tens of hours to seconds). Further, PFNet benefits considerably from increased number of peptides, demonstrating strong data efficiency and scalable performance (Supplementary Figs. 1, 2). By contrast, PFNet-Centroid suffers from reduced accuracy due to information loss from centroiding the data (Fig. 1b, Supplementary Fig. 4)^8^. Benchmarking shows that PFNet-Centroid can approach the accuracy of envelope-based PFNet, but typically requires more informative datasets: broader time-window coverage and greater peptide coverage, especially under higher noise conditions. Full isotopic envelopes therefore remain preferable whenever available (Supplementary Figs. 1, 4). Ablating the convolutional neural network (CNN) that pre-encodes isotopic mass envelopes markedly reduced predictive accuracy, highlighting the essential roles of both envelope information and CNN-based encoding (Fig. 1b, Supplementary Fig. 5). The final PFNet model embeds this CNN-encoded envelope information within its architecture to achieve optimal performance. When predictions were filtered based on their confidence scores, the RMSE was reduced, indicating that the confidence score is a reliable proxy for prediction accuracy, particularly in sparse and noisy datasets. The full PFNet model achieves the highest proportion of confident predictions (residues with confidence >0.8, see red dot size in Fig. 1b), providing both the most confident and most accurate results. Although PFNet-Centroid shows comparatively reduced overall performance, its confident predictions remain reliable.

Satisfied with the theoretical accuracy of PFNet, we then compared its predictions to the experimental HX-NMR-derived ∆G_op_ for three proteins: two small *de novo-*designed mini-proteins EEHEE_rd4_087 (Fig. 1d, Supplementary Figs. 6–8, Supplementary Tables 2, 3), HHH_rd4_0557 (Supplementary Fig. 9, Supplementary Tables 3, 4) and T4 lysozyme (T4L) (Supplementary Figs. 10, 11, Supplementary Tables 6, 7)^9,10^. Although HX-NMR can determine residue-level ∆G_op_, only about 100 datasets are available due to technical limitations of the technique. HX-NMR is ideal for small proteins in a narrow pH range that are isotopically labeled and purified, and it can take months to make the resonance assignments. For both mini-proteins and T4L, PFNet produced ∆G_op_ values that closely matched those determined by HX-NMR (Fig. 1d, Supplementary Figs. 6, 9, 11), with strong overall agreement (Pearson R: 0.63–0.91, RMSE: 2.49–5.53 kJ/mol). PFNet confidence was also strongly associated with prediction error (Supplementary Fig. 11), supporting its use as a reliable indicator of prediction accuracy. An approximate Bayesian assessment using Monte Carlo dropout, a measure of prediction uncertainty, further confirmed this reliability, showing a strong correlation between the posterior standard deviation and predicted absolute error (R: 0.41–0.82, Supplementary Fig. 12).

As an internal benchmark for direct comparison to HX-MS data, we constructed NMR-resolved peptides for single residue-resolved regions in the three benchmark proteins (Supplementary Fig. 13). PFNet reconstructed the HX-MS data more accurately than the HX-NMR-derived values for HHH_rd4_0557 and T4L. Interestingly, the HX-NMR-derived values reproduced the HX-MS data very well for both mini-proteins, but less well for T4L. This result suggests closer agreement between the HX-NMR and HX-MS experiments for the mini-proteins, and greater experimental divergence for T4L.

Benchmarking against other methods for determining ∆G_op_ (HDsite^11^, exPfact^12^, BayesianHDX^13^, FEATHER^3^, HDfleX^14^) on the same datasets further demonstrated that PFNet consistently delivered the strongest overall performance across all evaluated settings (Supplementary Figs. 14–16, Supplementary Notes). Additionally, comparison with the widely used empirical Best/Vendruscolo model^15^ showed moderate agreement (Supplementary Fig. 17), with correlation coefficients ranging from 0.19 to 0.48 and RMSE values ranging from 7.77 to 11.76 kJ/mol, reflecting the limitations of estimating conformational energetics from single static structures.

Although the PFNet analysis agreed well with the HX-NMR results, the synthetic training data do not fully capture the complexity of real HX-MS experiments. Noise sources in HX-MS data are diverse and often systematic, arising from factors such as instrumentation, peptide identification, chromatographic behavior, back exchange, and data processing. Future development of PFNet will therefore benefit from more realistic experimental noise models and training datasets that incorporate experimentally derived variability, which may further improve its robustness and generalizability across HX-MS platforms and protein systems. In specialized cases, such as heavily glycosylated proteins, the model may also need to be retrained with features that account for altered intrinsic exchange kinetics within glycopeptides and the contribution of glycans to overall deuterium retention^16^. To contextualize this limitation and estimate the noise level inherent in the experimental HX-MS datasets in comparison with the benchmarks, we used the prediction interval coverage probability (PICP)^17^ relative to the theoretical intervals of the synthetic noise added during training (Fig. 1e, see “Methods”). The variance equivalent interval measures how well the simulated noise reproduces the variance of the experimental residuals, whereas the support interval compares the maximum range of the possible deviations. The equivalent noise level was defined as the point at which PICP reached the nominal 0.95 coverage. The estimated noise levels of EEHEE_rd4_087 (Fig. 1e), HHH_rd4_0557 and T4L (Supplementary Fig. 18) are 0.03, 0.04 and 0.04, respectively, suggesting that the variation of these datasets is low. This correspondence allows the expected prediction accuracy for datasets to be inferred directly from the synthetic benchmarks (Fig. 1b, Supplementary Fig. 18), in agreement with the NMR benchmarks. Additionally, PFNet is robust not only to noise in the synthetic benchmarks but also in experimental datasets, with additional noise injections showing that prediction accuracy remained stable (Supplementary Fig. 19).

**Fig. 2:**
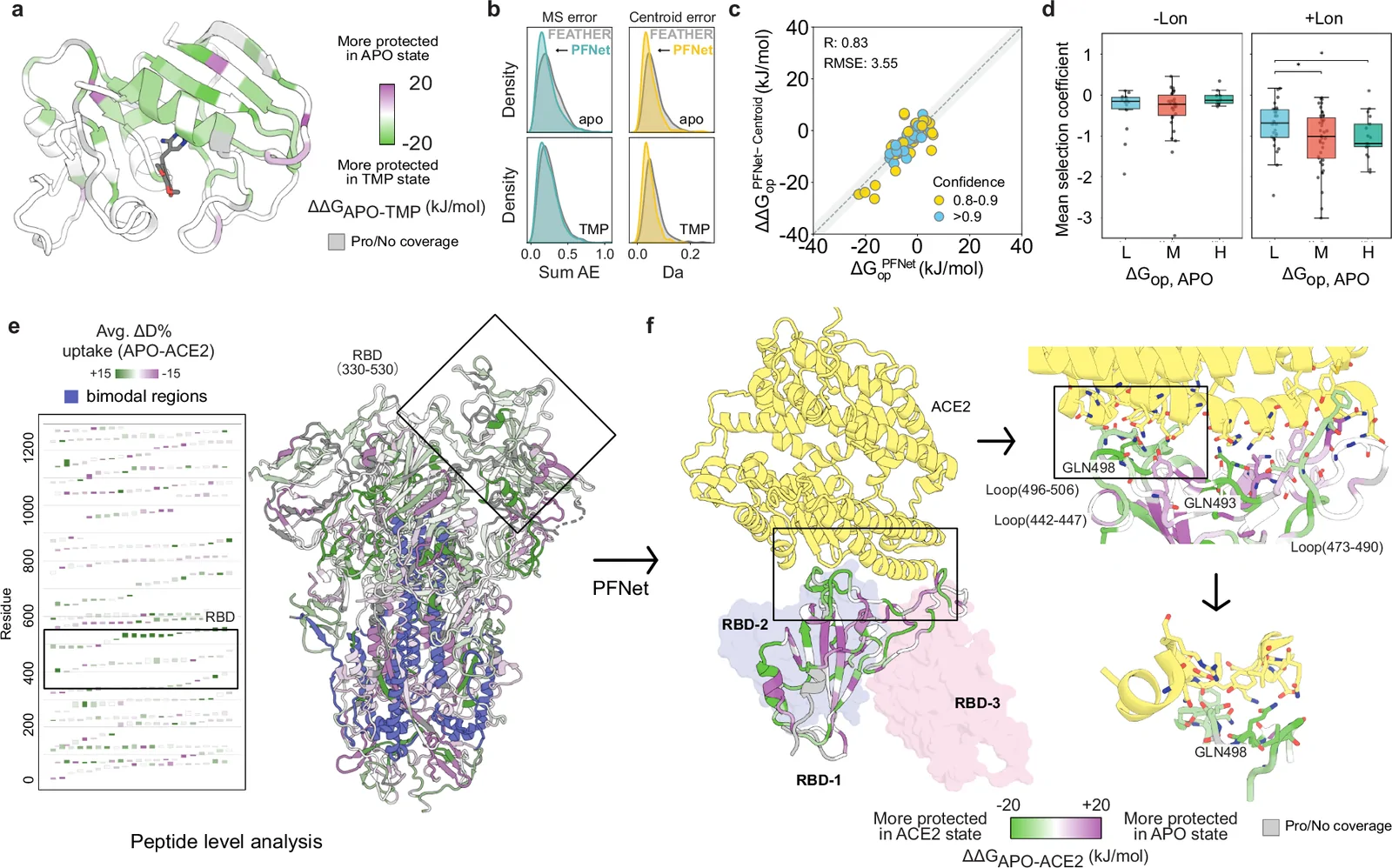
PFNet reveals how therapeutic targets respond to binding partners. **a** PFNet-derived ∆∆G_op(apo-TMP)_ values mapped onto the crystal structure of ecDHFR (PDB: 6XG5). **b** Comparison of fitting errors between PFNet and FEATHER-derived results on ecDHFR dataset, shown as distributions of summed absolute errors for mass spectra and absolute fitting errors for centroids (Supplementary Fig. 13). **c** Correlation between PFNet-derived and PFNet-Centroid-predicted ∆∆G_op(apo-TMP)_ values for ecDHFR. The error band indicates ± RMSE along the diagonal between the values derived from two models. **d** Distribution of selection coefficients across three ∆G_op(apo)_ groups in the absence or presence of Lon protease in DMS, sorted by ∆G_op(apo)_ as defined by the 25th and 75th percentiles: Low (< 9.5 kJ mol^−1^), Medium (9.5–24.1 kJ mol^−1^), and High (>24.1 kJ mol^−1^). For box plots, center lines indicate medians, boxes show the interquartile range, whiskers extend to 1.5 × the interquartile range, and black dots represent individual data points. Significant pairwise differences determined by the two-sided Mann–Whitney *U*-test without adjustment for multiple comparisons are indicated by “*” (*p* < 0.05). *p*-values are as follows: +Lon (L) vs. +Lon (M), *p* = 0.043; +Lon (L) vs. +Lon (H), *p* = 0.044. The ∆G_op(apo)_ values were derived from ecDHFR HX-MS data pooled across 7 replicates. **e** Peptide-level analysis of deuterium uptake differences in the SARS-CoV-2 spike protein between the apo and ACE2-bound states. Bimodal regions are shown in blue (Supplementary Fig. 15). **f** PFNet-derived ∆∆G_op_ profiles enable high-resolution and unbiased thermodynamic epitope mapping.

HX-MS can reveal how a protein’s ensemble responds to molecular perturbations like ligand binding and mutations. To determine how well PFNet captured energetic differences between protein functional states (∆∆G_op_), we compared its performance to FEATHER for apo and trimethoprim (TMP)-bound *E. coli* dihydrofolate reductase (ecDHFR). TMP is an antibiotic that stabilizes a distinct conformational state of ecDHFR^3^. PFNet recapitulated the FEATHER results (Fig. 2a, Supplementary Fig. 20) with lower fitting errors (Fig. 2b, Supplementary Fig. 21), which suggests that the PFNet-derived ∆G_op_ values more closely match the experimental observations. Notably, the predictions of PFNet-Centroid showed good correlation with those of PFNet (Fig. 2c), as expected from the synthetic dataset results.

Interestingly, in comparing the apo ecDHFR PFNet results with a deep mutational scanning (DMS) dataset collected by Thompson et al^18^., we find that highly protected residues show significant drops in DMS selection coefficients upon mutation in the presence of the global regulatory protease Lon, but remain comparatively tolerant to mutation in its absence (Fig. 2d). Although this trend is partly expected from residue burial, given that solvent-accessible surface area (SASA) is positively correlated with ∆G_op_ (Supplementary Fig. 22), ∆G_op_ is not equivalent to SASA because it reflects the energetic cost of local unfolding and thereby captures additional features such as local packing, hydrogen bonding, and conformational flexibility. This indicates that mutations in the most locally stable regions of ecDHFR become significantly less tolerated when Lon protease is active, reflecting increased sensitivity to local destabilization and partial unfolding. Thus, understanding residue-level ∆G_op_ provides a thermodynamic basis for predicting mutational tolerance, offering valuable guidance for protein stability engineering and directed evolution.

To investigate how PFNet might illuminate viral protein-host interactions, we applied the method to the SARS-CoV-2 spike protein ectodomain interaction with the human ACE2 receptor, using an HX-MS dataset collected on a different mass spectrometer by a different group (Supplementary Tables 8, 9)^19^. The spike-ACE2 interaction is the first step of viral cell invasion. Notably, such large complexes are intractable to HX-NMR. Comparing the peptide-level qualitative analysis with the residue-level PFNet analysis (Fig. 2e, f, Supplementary Figs. 23, 24), we found that the PFNet-derived ∆∆G_op_ values enable high-resolution and unbiased thermodynamic epitope mapping, providing quantitative insights that are inaccessible to conventional peptide-level HX-MS. At the peptide level (Supplementary Fig. 23), the affected region appears as a broad and relatively weak protection pattern, without sufficient resolution to distinguish differential stabilization among overlapping segments or assign the strongest effect to specific residues. In contrast, this analysis revealed that loops 497–506 and 442–447 are more strongly protected than loop 473–482, whereas loop 473–482 shows partial destabilization. Among all residues, Gln498 and Gln493 exhibit the strongest protection, pinpointing these as the thermodynamic centers of ACE2 recognition (Fig. 2f). These findings are consistent with prior structural^20,21^ and mutational studies^22,23^: X-ray crystal structures show that Gln498 and Gln493 form key hydrogen bonds with ACE2^20,21^, which informed the design of ACE2 receptor traps as therapeutics^24^; DMS demonstrated that substitutions of Gln498 and Gln493 alter ACE2 affinity, defining them as a functional hotspots^22,23^; and molecular dynamics simulations revealed a long-lived contact between Gln498 and ACE2 Lys353^25^. In the Omicron variant of SARS-CoV-2, RBD mutations Q493R and G496S further increase the binding affinity via new hydrogen bonds with ACE2 Glu35 and Lys353^26^. Together, these observations reinforce the roles of Gln498 and Gln493 as dominant contributors to ACE2 binding stability.

Moreover, the ∆∆G_op_ profile derived from PFNet revealed coupled thermodynamic changes upon ACE2 binding. The ACE2 binding-induced population shift toward the RBD “up” conformation^20,27^ leads to increased protection and rigidity in several loop regions (Supplementary Fig. 24). This local stabilization is accompanied by destabilization of the central β-sheet, suggesting an enthalpy-entropy compensation mechanism^28,29^ that preserves the overall spontaneity of ACE2 binding (Fig. 2f). Structural comparisons between the RBD “up” and “down” conformations support this interpretation, showing that apart from increased electron density in loop regions, the global fold remains nearly identical (root-mean-square deviation of 1.42 Å)^30,31^. The quantitative HX-MS pipeline also enabled the identification of additional bimodal regions to those reported^19^; these regions have been shown to report on two slowly-interconverting conformations of the same regions that exist simultaneously in solution (Supplementary Fig. 25). These results show that PFNet resolves allosterically coupled thermodynamic changes between local loop protection and central β-sheet destabilization that cannot be captured by static structural analyses alone, even in large, dynamic complexes.

HX-MS experiments should be designed towards optimizing the data analysis. An ideal dataset for PFNet is easy to collect: three pooled technical replicates, each with different timepoints and proteases, alongside fully deuterated controls for back-exchange correction and tandem MS experiments for peptide identification. The inclusion of data points across a large time window spanning seconds to 24+ h is more valuable than dense data points in a narrow time window. Data collection and analysis can be completed in 1–2 days, with less than an hour of hands-on instrument time if the experiment is automated^32^. For example, Supplementary Fig. 1 shows that, for a protein with a range of ∆G_op_ as in our benchmarks (Supplementary Table 1), approximately five timepoints spanning 10^1^ to 10^5^ s are sufficient to achieve a predicted error below 5 kJ/mol, even at a high noise level of 0.3. This level of accuracy requires unique peptide coverage roughly matching the protein length, for example, 100 unique peptides for a 100-residue protein, together with an average redundancy of about 10.

While HX-MS remains popular for qualitative epitope-mapping applications, here we report how coupling PFNet with a standard HX-MS workflow can reveal detailed biomolecular mechanisms, making full use of the information-rich data without complicating the experiment. Although confidence filtering reduces residue-level coverage, it does not eliminate information from the experiment, because the original peptide-level HX-MS data remain available; PFNet simply provides a higher-resolution thermodynamic interpretation for residues that are well-constrained by the data. The method is transformative: compared to other methods for high-resolution ∆G_op_ determination, PFNet maintains higher accuracy under reduced peptide coverage and narrower experimental time windows (Supplementary Figs. 1 vs. 3), is dramatically faster and easier to use, thereby democratizing HX-MS data collection and analysis such that all labs can use the method to extract the maximum amount of information about protein ensembles. The increased accuracy afforded by PFNet allows for the detection of subtle but crucial energetic differences among protein functional states within a single exceptionally sensitive framework, enabling a detailed understanding of biological machines. PFNet can be applied to hundreds or thousands of proteins simultaneously, or to proteins with hundreds or thousands of residues, to understand their free energy landscapes and to generate large and accurate datasets for future machine learning applications. By providing a high-resolution energetic blueprint for protein interactions or mutational effects, PFNet may enable the precise rational design of allosteric therapeutics. The SI describes how to access the benchmark and experimental HX-MS datasets, convert HX-MS data for compatibility with PFNet, and use PFNet locally or on the cloud-based public installation.

## Methods

### Model

PFNet was built using the deep-learning Python packages PyTorch^33^ and PyTorch-Lightning^34^. PFNet employs a conventional encoder–decoder transformer architecture tailored for peptide-level HX-MS data. For each time point of each peptide, the isotopic mass envelope is first processed by a one-dimensional convolutional neural network (1D-CNN), which captures local spectral features. The resulting embeddings are concatenated with time encodings and peptide positional encodings to form a unified timepoint token representation. These timepoint tokens are subsequently passed through the transformer encoder, which models the temporal and spatial dependencies across all peptides and timepoints. The decoder queries consist of residue-level representations, incorporating information about intrinsic exchange rates (${k}_{{{{\rm{ch}}}}}$, the exchange rate for an unfolded peptide) and the resolution limits imposed by the peptide coverage of the dataset. PFNet outputs both the predicted residue-level ${\log }_{10}{k}_{{{{\rm{ex}}}}}$ values and their associated confidence scores by separate output heads using the same decoder embeddings. The confidence head is trained by mapping the predicted confidence values to the predicted absolute error using a single exponential decay function. The decoder also employs an iterative recycling mechanism, in which predicted outputs from the previous iteration are incorporated into the query embeddings, and the corresponding reconstruction errors are included as part of the key embeddings. This design allows the model to iteratively refine its predictions by leveraging prior outputs and their associated reconstruction errors. We provide pseudocode for PFNet in Supplementary Note 1. These predicted ${\log }_{10}{k}_{{{{\rm{ex}}}}}$ are subsequently used to compute the ∆G_op_. Although PFNet was trained on proteins with 100–400 residues to balance generality and computational tractability, 400 residues does not represent a hard limit at inference time. For proteins with more than 400 residues, the input is divided into overlapping fragments of 200 residues, with 100-residue contextual padding on each side as needed. Using this strategy, we tested PFNet across proteins of varying size and did not observe a substantial loss of accuracy, including for synthetic proteins up to 5000 residues (Supplementary Fig. 26). In principle, this fragmentation-based inference scheme allows the model to be applied to proteins of essentially arbitrary length.

### Loss function

We designed a composite loss function that balances predictive accuracy and physical consistency during model training:

1 $${{{{\mathcal{L}}}}}_{{{{\rm{total}}}}}={{{{\mathcal{L}}}}}_{{{{\rm{mse}}}}}+{\lambda }_{{{{\rm{conf}}}}}{{{{\mathcal{L}}}}}_{{{{\rm{conf}}}}}+{\lambda }_{{{{\rm{env}}}}}{{{{\mathcal{L}}}}}_{{{{\rm{env}}}}}+{\lambda }_{{{{\rm{cent}}}}}{{{{\mathcal{L}}}}}_{{{{\rm{cent}}}}}+{\lambda }_{{{{\rm{kch}}}}}{{{{\mathcal{L}}}}}_{{{{\rm{kch}}}}}$$

Here, C is the number of recycling cycles; ${{{{\mathcal{M}}}}}_{{{{\rm{res}}}}}$ denotes the set of valid residues included in the residue-level loss calculation; and ${{{{\mathcal{M}}}}}_{{{{\rm{env}}}}}$ denotes the set of valid peptide-timepoint observations included in the envelope and centroid loss calculations. For residue i at recycling cycle c, ${\widetilde{y}}_{i}^{\left(c\right)}$ denotes the predicted residue-level ${\log }_{10}{k}_{{{{\rm{ex}}}}}$, ${\widetilde{y}}_{i}^{*}$ denotes the corresponding ground-truth value, ${\widetilde{u}}_{i}^{\left(c\right)}$ denotes the predicted confidence score, the confidence target is denoted by ${q}_{i}^{\left(c\right)}$, and ${k}_{{{{\rm{ch}}}},i}$ denotes the intrinsic chemical exchange rate.

The residue-level mean squared error is:

2 $${{{{\mathcal{L}}}}}_{{{{\rm{mse}}}}}=\frac{1}{C} {\sum}_{c=1}^{C}\,\frac{1}{\left|{{{{\mathcal{M}}}}}_{{{{\rm{res}}}}}\right|} {\sum}_{i\in {{{{\mathcal{M}}}}}_{{{{\rm{res}}}}}}\,{\left({\widetilde{y}}_{i}^{\left(c\right)}-{\widetilde{y}}_{i}^{*}\right)}^{2}$$

The confidence loss is defined as a Smooth L1 loss between the predicted confidence score and a target derived from the absolute prediction error:

3 $${q}_{i}^{\left(c\right)}=\exp \left(-0.2\left|{\tilde{y}}_{i}^{\left(c\right)}-{\tilde{y}}_{i}^{*}\right|\right)$$

4 $${{{{\mathcal{L}}}}}_{{{{\rm{conf}}}}}=\frac{1}{C} {\sum}_{c=1}^{C}\,\frac{1}{\left|{{{{\mathcal{M}}}}}_{{{{\rm{res}}}}}\right|} {\sum}_{i\in {{{{\mathcal{M}}}}}_{{{{\rm{res}}}}}}\,{{{\rm{SmoothL}}}}1\left({\widetilde{u}}_{i}^{\left(c\right)},{q}_{i}^{\left(c\right)}\right)$$

The ${k}_{{{{\rm{ch}}}}}$ consistency term penalizes predictions that exceed the intrinsic exchange rate limit:

5 $${{{{\mathcal{L}}}}}_{{{{\rm{kch}}}}}=\frac{1}{C} {\sum}_{c=1}^{C}\,\frac{1}{\left|{{{{\mathcal{M}}}}}_{{{{\rm{res}}}}}\right|} {\sum}_{i\in {{{{\mathcal{M}}}}}_{{{{\rm{res}}}}}}\,\max \left(0,{\widetilde{y}}_{i}^{\left(c\right)}-{k}_{{{{\rm{ch}}}},i}\right)$$

Let ${{{{\bf{p}}}}}_{p,t}\in {{\mathbb{R}}}^{B}$ denote the observed isotopic envelope for peptide p at labeling time t, and let ${\hat{{{{\bf{p}}}}}}_{p,t}\in {{\mathbb{R}}}^{B}$ denote the corresponding reconstructed envelope. Envelope reconstruction is performed using the same forward calculation as previously reported, given the predicted exchange rates^3^. Then the envelope loss is:

6 $${{{{\mathcal{L}}}}}_{{{{\rm{env}}}}}=\frac{1}{\left|{{{{\mathcal{M}}}}}_{{{{\rm{env}}}}}\right|} {\sum}_{(p,t)\in {{{{\mathcal{M}}}}}_{{{{\rm{env}}}}}}\, {\sum}_{b=1}^{B}\,{{{\rm{SmoothL}}}}1\left({\hat{p}}_{p,t,b},{p}_{p,t,b}\right)$$

The centroid for each peptide-timepoint is computed from the isotopic envelope as:

7 $${\hat{c}}_{p,t}={\sum}_{b=1}^{B}\,(b-1){\hat{p}}_{p,t,b},{c}_{p,t}={\sum}_{b=1}^{B}\,(b-1){p}_{p,t,b}$$

and the centroid loss is:

8 $${{{{\mathcal{L}}}}}_{{{{\rm{cent}}}}}=\frac{1}{\left|{{{{\mathcal{M}}}}}_{{{{\rm{env}}}}}\right|} {\sum}_{(p,t)\in {{{{\mathcal{M}}}}}_{{{{\rm{env}}}}}}\,{{{\rm{SmoothL}}}}1\left({\hat{c}}_{p,t},{c}_{p,t}\right)$$

Loss weights were adjusted according to a fixed two-stage training schedule to emphasize different objectives at different phases of training, as described in the Training section.

### Synthetic training datasets

Synthetic datasets were generated using an established method^3^. These datasets span a wide range of conditions, including protein sizes from 100–400 residues, peptide number ranging from 0.7–1.3 times the protein length, peptide lengths between 3 and 48 residues, labeling time windows from 10^−6^–10^18^ s and back exchange from 0.0–0.8, so as to mimic the range of conditions observed in experimental datasets, such as the *ec*DHFR data previously reported by our laboratory^3^. Importantly, although labeling times were sampled from a broad window (10^−6^–10^18^ s), each synthetic protein was not forced to span the entire time range. Instead, time points were sampled randomly, with a higher probability (60%) assigned to ranges that are more commonly used in typical HX-MS experiments (10^1^–10^6^ s). To expose the model to a broad range of possible experimental perturbations, we incorporated diverse noise sources during training, including additive noise to isotopic envelopes, back exchange, and side-chain exchange effects, all scaled by a global parameter (noise level nl). At non-zero time points, side-chain exchange noise was added as a small positive offset up to ${nl}/10$ of the theoretical maximum uptake. Back exchange levels were perturbed with Gaussian noise proportional to ${nl}/5$. Here, side-chain exchange was included only as an additional perturbation to improve robustness to this potential source of experimental variability, ranging from 0 to ${nl}/10$ multiplied by the theoretical maximum uptake. Isotopic envelopes were further perturbed by baseline fluctuations proportional to both nl and peak intensity, together with a time-dependent term scaled by $(({nl})/10){\log }_{10}(t)$. All envelopes were truncated to non-negative values and renormalized. The nl ranges from 0.1 to 0.3 in the training. Synthetic data were generated continuously in an online manner during training to ensure diversity and improve model generalization.

### Synthetic benchmarking datasets

Benchmarking datasets were generated in the same manner as the synthetic training datasets, with controlled protein size, peptide number, time windows, and noise levels to assess their effects on model performance (Supplementary Table 1). For each dataset, five different proteins were generated to ensure adequate diversity and statistical robustness. Note: The noise sources differ from those used in the benchmarking dataset for PIGEON-FEATHER^3^. We therefore add the prefix PFsim to indicate this distinction.

### Training

PFNet training was conducted in two stages. The ${{{{\mathcal{L}}}}}_{{{{\rm{mse}}}}}$ was applied throughout both phases. In phase 1, ${\lambda }_{{{{\rm{conf}}}}}$ and ${\lambda }_{{{{\rm{kch}}}}}$ was set to 0, and only the MSE and reconstruction losses, including envelope and centroid reconstruction, were used, with the HX-MS-related losses ${\lambda }_{{{{\rm{env}}}}}{{{{\mathcal{L}}}}}_{{{{\rm{env}}}}}$ and ${\lambda }_{{{{\rm{cent}}}}}{{{{\mathcal{L}}}}}_{{{{\rm{cent}}}}}$ weighted at approximately 10% of ${{{{\mathcal{L}}}}}_{{{{\rm{mse}}}}}$ term. This allowed the model to focus on learning accurate ${\log }_{10}{k}_{{{{\rm{ex}}}}}$ and reproducing peptide-level envelopes with respect to the HX physics. In phase 2, the confidence loss was enabled with ${\lambda }_{{{{\rm{conf}}}}}{{{{\mathcal{L}}}}}_{{{{\rm{conf}}}}}$ a weight of 10% and the weights of reconstruction losses were reduced to 5% of ${{{{\mathcal{L}}}}}_{{{{\rm{mse}}}}}$ term, shifting the emphasis toward predictive accuracy and uncertainty calibration while reducing the risk of overfitting to synthetic noise. Training was performed on two NVIDIA RTX 4090 GPUs for two weeks. The training dataset comprised over 100 million synthetic protein examples, corresponding to approximately 10 billion simulated mass spectra.

### Back exchange modeling

There are two common approaches for modeling back exchange in the forward HX calculations, which convert residue-level ${\log }_{10}{k}_{{{{\rm{ex}}}}}$ values into peptide-level isotopic mass envelopes. The first approach assigns a uniform back exchange value to each residue, independent of its peptide sequence context^11^. The second approach distributes the peptide-level measured back exchange evenly across residues within a peptide^3^. Both methods rely on a widely used assumption that the first or first and second residues of a peptide do not retain deuterium, *i.e*., their back exchange is 100%. However, this assumption is not universally valid for the second residue, which heavily depends on the sequence context^35^. HX-NMR experiments show that back exchange behavior is primarily governed by ${k}_{{{{\rm{ch}}}}}$, the intrinsic exchange rates of residues in the unfolded state, which are influenced by both solution conditions and the peptide sequence. For example, when a peptide begins with bulky residues such as “PI,” the second residue “I” significantly contributes to deuterium retention. In contrast, internal sequence motifs like “HH” can exhibit complete back exchange at the second “H” residue^35,36^. These observations highlight that residue-specific back exchange is heterogeneous and sequence-dependent.

To incorporate such context-dependent heterogeneity into back exchange modeling, we introduce an equivalent back exchange time under ideal quench conditions (pH 2.3, 0 °C). A similar back exchange correction strategy has been proposed previously^37^, in which back exchange was modeled using two peptide-specific effective times and two global effective-time parameters, whereas here we use a simpler implementation with a single effective time for each peptide to reduce model complexity and limit overfitting. For each peptide p, this effective time, ${t}_{{{{\rm{ideal}}}},p}$, is fitted to reproduce the experimentally measured fully deuterated value and is then used to infer residue-level back exchange values within that peptide. This formulation provides a sequence-aware refinement of conventional back exchange correction.

Let ${D}_{{{{\rm{theo}}}},p}$ denote the theoretical deuterium retention for peptide p, and let ${D}_{{{{\rm{theo}}}},p,i}$ denote the contribution from residue i within peptide p. Let ${k}_{{{{\rm{ch}}}},p,i}$ denote the intrinsic exchange rate of residue i in peptide p under ideal quench conditions, ${t}_{{{{\rm{ideal}}}},p}$ the fitted equivalent back exchange time for peptide p, and ϕ the D_2_O saturation level. The theoretical deuterium retention is then given by:

9 $${D}_{{{{\rm{theo}}}},p}={\sum}_{i}\,{D}_{{{{\rm{theo}}}},p,i}=\phi {\sum}_{i}\,\exp \left(-{k}_{{{{\rm{ch}}}},p,i}{t}_{{{{\rm{ideal}}}},p}\right)$$

The parameter ${t}_{{{{\rm{ideal}}}},p}$ is determined using the Brent root-finding algorithm (scipy.optimize.brentq) such that the calculated peptide-level deuterium retention matches the experimentally measured fully deuterated value for peptide p. The back exchange value of residue i in the context of peptide p, denoted by ${\beta }_{p,i}$, is then defined as:

10 $${\beta }_{p,i}=1-\frac{{D}_{{{{\rm{theo}}}},p,i}}{\phi }=1-\exp \left(-{k}_{{{{\rm{ch}}}},p,i}{t}_{{{{\rm{ideal}}}},p}\right)$$

We also empirically compared this back-exchange strategy with the two alternative models described above using NMR-resolved peptide regions from the two mini-proteins in our benchmark (Supplementary Fig. 27). The approach used in the current study performed best overall, the uniform residue-level model performed reasonably well, and the peptide-average model performed worst.

### Estimation of equivalent experimental noise level

To estimate the experimental noise level that is consistent with synthetic data, residuals were compared against the theoretical intervals of the synthetic two uniform noise used during training. We computed the prediction interval coverage probability (PICP) using two complementary interval definitions:

1. Variance equivalent interval, which reflects agreement in terms of variance.

Let nl denote the noise level, and let a and b denote the half-widths of the two uniform noise components, defined as:

11 $$a=\frac{{nl}}{10},b={nl}\cdot s$$

where s denotes the normalized peak intensity. The variance-equivalent interval is then given by:

12 $$[-{h}_{{{\mathrm{var}}}},{h}_{{{\mathrm{var}}}}],{h}_{{{\mathrm{var}}}}=\sqrt{{a}^{2}+{b}^{2}}$$

Equivalently, let σ denotes the standard deviation of the combined synthetic noise, then

13 $${h}_{{{\mathrm{var}}}}=\sqrt{3}\sigma$$

2. Support interval, which provides a complementary check based on the maximum possible error range.

14 $$[-{h}_{\sup },{h}_{\sup }],{h}_{\sup }=a+b$$

The noise level at which the PICP reached the nominal 0.95 coverage was defined as the equivalent experimental noise level, thereby aligning experimental variation with synthetic benchmarking conditions.

### Monte Carlo dropout

For MC dropout analysis, the trained PFNet model was evaluated with Dropout layers selectively reactivated during inference to enable approximate Bayesian sampling. For each input, 100 stochastic forward passes were performed without parameter updates, and the residue-level predicted ${\log }_{10}{k}_{{{{\rm{ex}}}}}$ values were recorded. The uncertainty of each prediction was quantified as the standard deviation across the 100 sampled outputs.

### Statistical significance of ∆∆G_op_ comparisons

To compare two biological states, PFNet confidence scores were converted to predicted absolute errors using the same calibration function defined in the Loss function section. For residue i in state s,

15 $${\widehat{{{{\rm{AE}}}}}}_{i,s}=\frac{-\log {\widetilde{u}}_{i,s}}{0.2}$$

and the corresponding standard deviation was estimated as

16 $${\hat{\sigma }}_{i,s}={\widehat{{{{\rm{AE}}}}}}_{i,s}\sqrt{\frac{\pi }{2}}$$

The residue-level difference between states A and B was defined as

17 $$\Delta \Delta {G}_{{{{\rm{op}}}},i}=\Delta {G}_{{{{\rm{op}}}},i}^{\left(B\right)}-\Delta {G}_{{{{\rm{op}}}},i}^{\left(A\right)}$$

Assuming independent errors, the combined uncertainty was estimated by error propagation:

18 $${\hat{\sigma }}_{\Delta \Delta G,i}=\sqrt{{\hat{\sigma }}_{i,A}^{2}+{\hat{\sigma }}_{i,B}^{2}}$$

The propagated uncertainty was then used to interpret residue-level ∆∆G_op_ differences. Differences exceeding the associated propagated uncertainty were considered meaningful, whereas smaller differences were interpreted more cautiously as potentially reflecting noise-related variation.

### HX-MS data collection and analysis

For T4L, we introduced a T4 bacteriophage cysteine-free pseudo-wildtype gene with an N-terminal 6xHis tag into a modified pET9a plasmid via Golden Gate cloning using a BsaI restriction endonuclease to produce the pET9a6xHis-T4L plasmid. We chemically transformed the pET9a-6xHis-T4L plasmid into a BL21 LOBSTR strain. Cells were grown at 37 °C overnight, shaking at 200 rpm in LB media with 50 µg/ml kanamycin, then subcultured 1/100 into 0.5 L LB media with 50 µg/ml kanamycin and grown at 30 °C until the optical density at 600 nm was measured between 0.2 and 0.4. Protein expression was induced by the addition of 1 mM IPTG, and the cells were grown at 16 °C for 12–16 h. The culture was centrifuged at 6000 × *g* for 20 min, and the cell pellet was frozen at −20 °C. After several weeks, the pellet was thawed in 20 ml lysis buffer: 50 mM Tris, 150 mM NaCl, 0.5 mM TCEP, pH 8.0 with 5 mM MgCl2, 1 mM MnCl2, 100 µM CaCl2, a protease inhibitor cocktail (Pierce), and 2000 units/ml DNase I. The mixture was vortexed at 20 °C for 15 min, then sonicated in an ice bath at 30% amplitude for 10 min, with a four-second break following every two seconds of sonication to prevent overheating. Next, the lysate was centrifuged at 27,000 × *g* for 30 min at 4 °C. The soluble fraction was applied to 2 ml NiNTA resin in 5 ml sample buffer (50 mM Tris, 150 mM NaCl, 0.5 mM TCEP, pH 8.0), and the mixture was left to nutate overnight at 4 °C. The mixture was then added to a 20 ml benchtop gravity column, allowed to drain and settle, and washed with 50 mL wash buffer (50 mM Tris, 250 mM NaCl, 0.5 mM TCEP, 20 mM imidazole, pH 8.0). The protein was eluted in 14 ml of elution buffer (50 mM Tris, 150 mM NaCl, 0.5 mM TCEP, 200 mM imidazole, pH 8.0) and then purified further on a HiLoad 16/600 Superdex 200 size exclusion column (Cytiva). The eluent at the retention time corresponding to the monomeric molecular weight was concentrated to 0.5 mg/mL using a 15 ml ultracentrifugal filter column with a 10 KDa MWCO (MilliporeSigma). This purified stock was split into aliquots, flash frozen in liquid nitrogen, and stored at −80 °C. On the day of HX-MS analysis, the protein stocks were thawed, and the 25 µM sample was diluted tenfold into the exchange buffer (50 mM Tris, 150 mM NaCl, 0.5 mM TCEP, pH 8.0 in D_2_O). Details on data collection and analysis for T4L, ecDHFR, and the mini-protein, as well as the expression and purification of ecDHFR, were previously described^3^.

### Merging T4 lysozyme datasets collected at two pHs

To obtain a broader exchange time window, we combined seven replicate datasets collected at pH 8.0 and pH 6.0 (Supplementary Table 2). The measured timepoints at pH 8.0 were converted to their equivalents at pH 6.0 using the method described by Hamuro et al.^38,39^, which has been shown to effectively extend the time window range of exchange measurements for proteins that exhibit similar behavior across these pH conditions (Supplementary Fig. 8).

### Reporting summary

Further information on research design is available in the Nature Portfolio Reporting Summary linked to this article.

## Supplementary information

Supplementary Information

Description of Additional Supplementary Files

Supplementary Data 1

Supplementary Data 2

Reporting Summary

Transparent Peer Review file

## Source data

Source Data

## Data Availability

The data are provided in HXMS format^40^, which includes the isotopic mass envelope peaks or centroids for all timepoints and peptides as defined by starting and ending residues, as well as important experimental conditions that influence k_ex_ determination by the Linderstrøm-Lang model^41^: pH, D_2_O saturation level, and temperature. PFLink [https://huggingface.co/spaces/glasgow-lab/PFLink] enables the conversion of HX-MS data from commonly used softwares^40^. The datasets used in this study are available under the following accession codes: Zenodo 17353731^42^ [https://zenodo.org/records/17353731] (synthetic benchmark dataset); PXD069693 (raw HX-MS data for T4 lysozyme and HHH_rd4_0557). Source data are provided with this paper.

## Code availability

Code for dataset generation, model training, inference, and access to trained models is publicly available and has been deposited in GitHub at https://github.com/glasgowlab/PFNet, under the MIT License. The specific version of the code associated with this publication is archived in Zenodo and is accessible via 10.5281/ZENODO.17353731^43^ To facilitate broader accessibility, the trained model is also hosted on Hugging Face at https://huggingface.co/spaces/glasgow-lab/PFNet.
