# Supplementary material for: A machine learning method for calculating highly localized protein stabilities
Source: Nat Commun. 2026 Sep 1;17:10414. doi: 10.1038/s41467-026-75590-9 (PMC13631364; doi:10.1038/s41467-026-75590-9)
Supplement: Supplementary file 1 [file 41467_2026_75590_MOESM1_ESM.pdf]

Supplementary figures

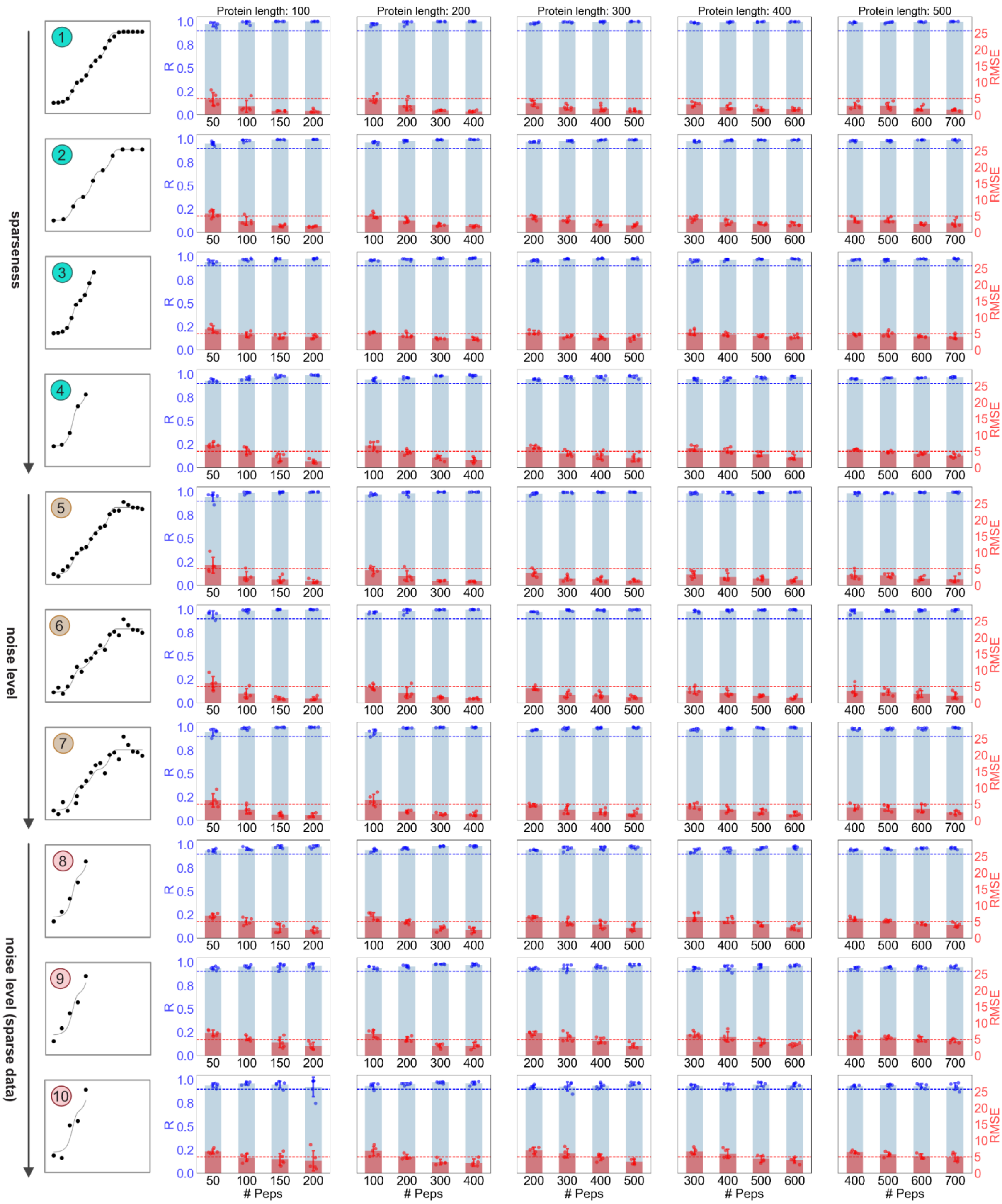

Supplementary Figure 1. Expanded data of Fig. 1b for PFNet predictions with conf. > 0.8.

PFNet performance on synthetic datasets (Supplementary Table 1) with varying degrees of sparseness and noise across proteins of different sizes and peptide numbers, with R (blue) and RMSE (red). Data are shown as mean ± s.d. from five independently generated proteins per dataset.

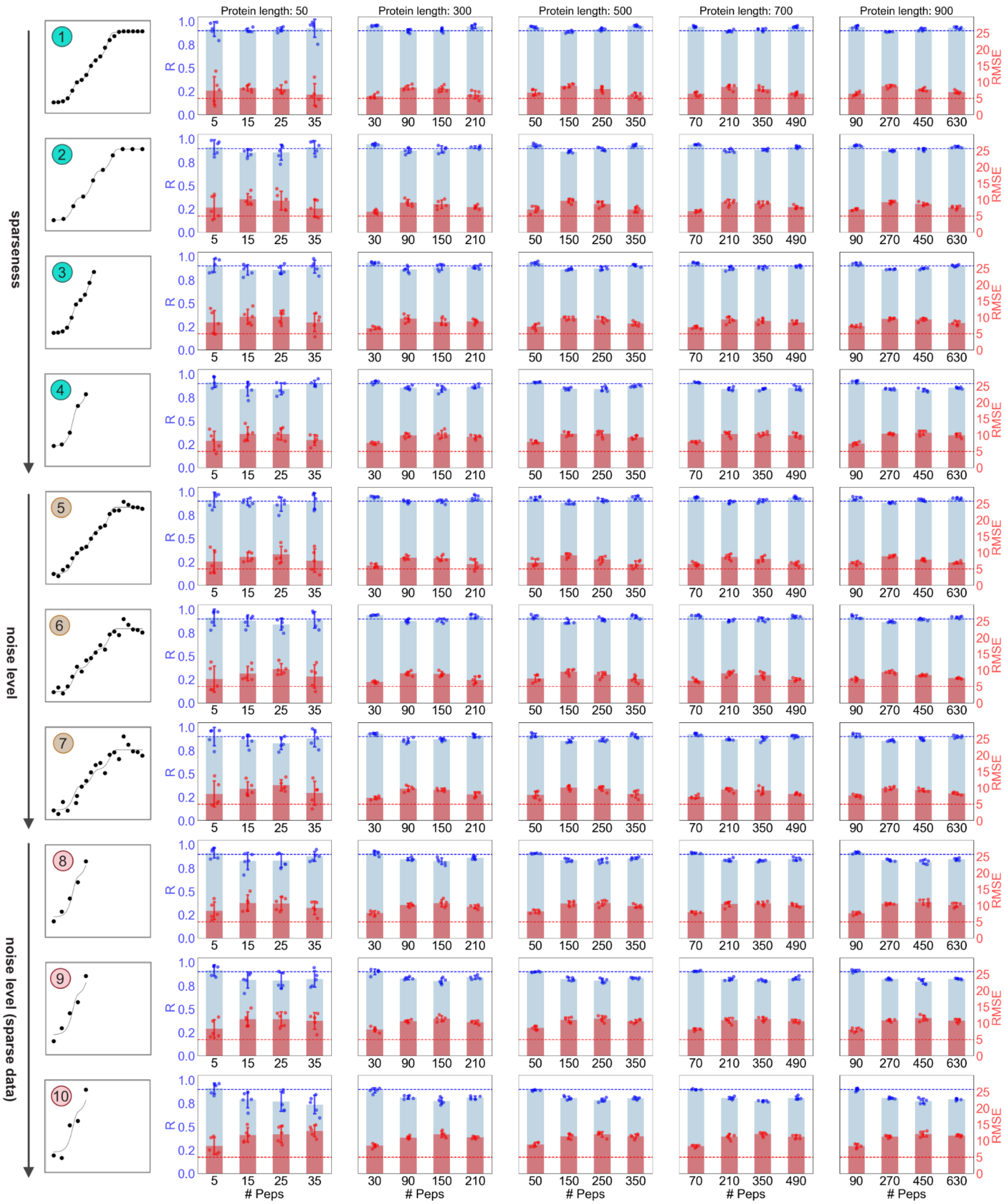

Supplementary Figure 2. PFNet predictions with conf. > 0.8 for datasets with peptide coverage outside the training window.

PFNet performance on synthetic datasets (Supplementary Table 1) with varying levels of sparseness and noise, across proteins of different sizes and peptide numbers outside the training window. Data are shown as mean ± s.d. from five independently generated proteins per dataset.

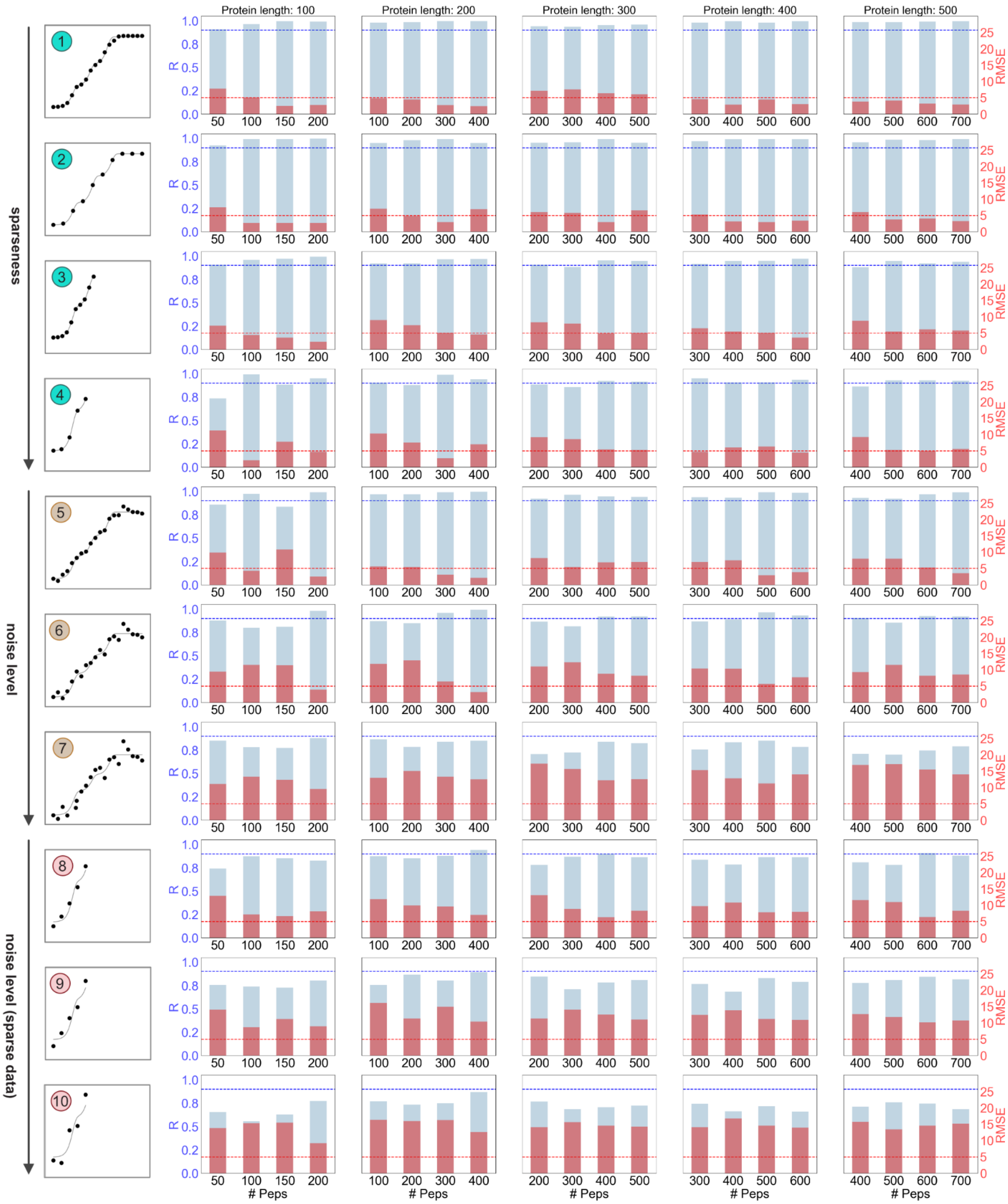

Supplementary Figure 3. FEATHER results with uncertainties < 1.0 log(k_{ex}).

FEATHER performance on synthetic datasets (Supplementary Table 1), with R (blue) and RMSE (red). Only one protein was evaluated for each case due to the computational cost of running FEATHER on so many proteins.

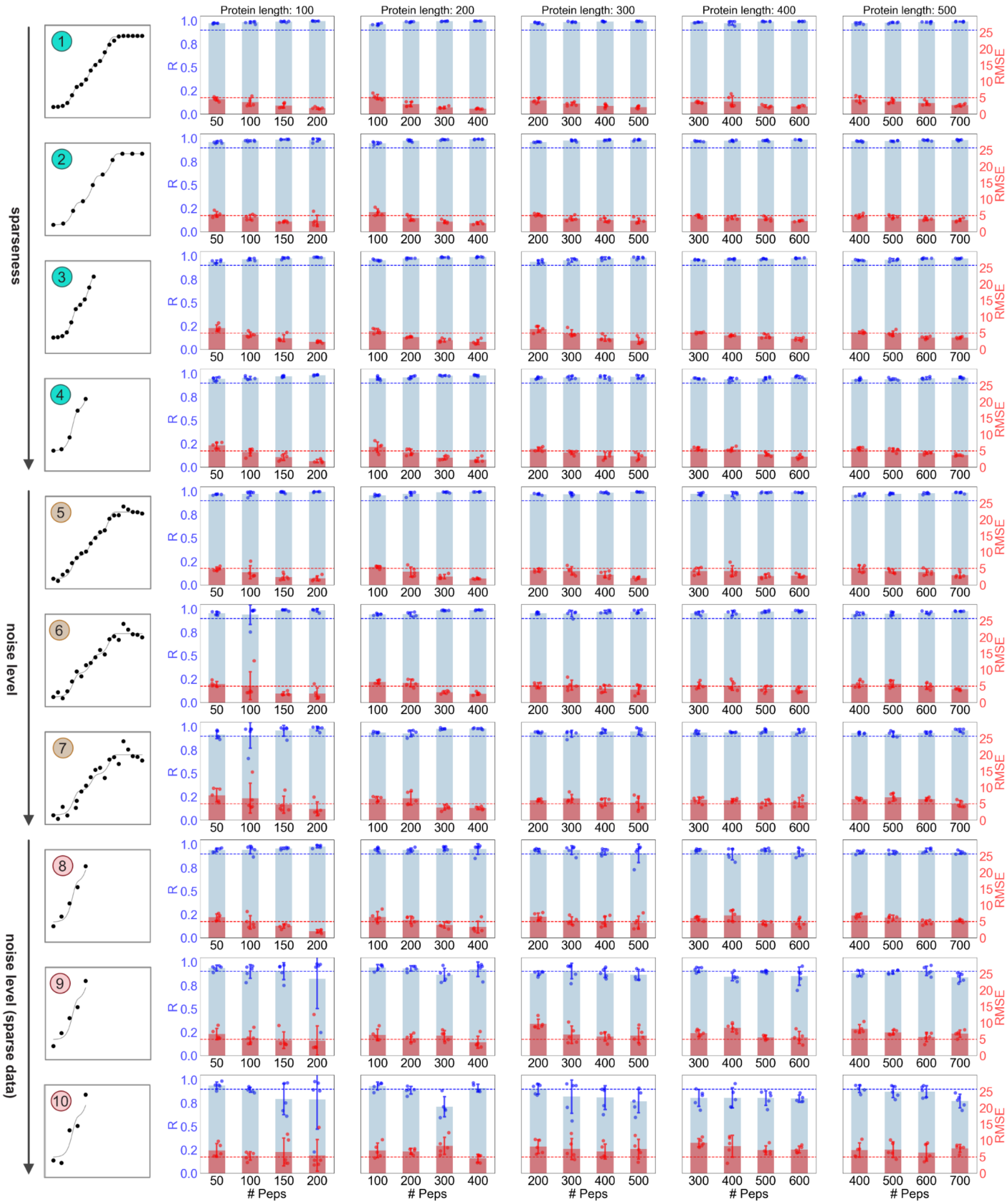

Supplementary Figure 4. Expanded data of Fig. 1b for PFNet-Centroid predictions with confidence > 0.8.

PFNet-Centroid performance on synthetic datasets with varying degrees of sparseness and noise across proteins of different sizes and peptide numbers, with R (blue) and RMSE (red). Data are shown as mean ± s.d. from five independently generated proteins per dataset.

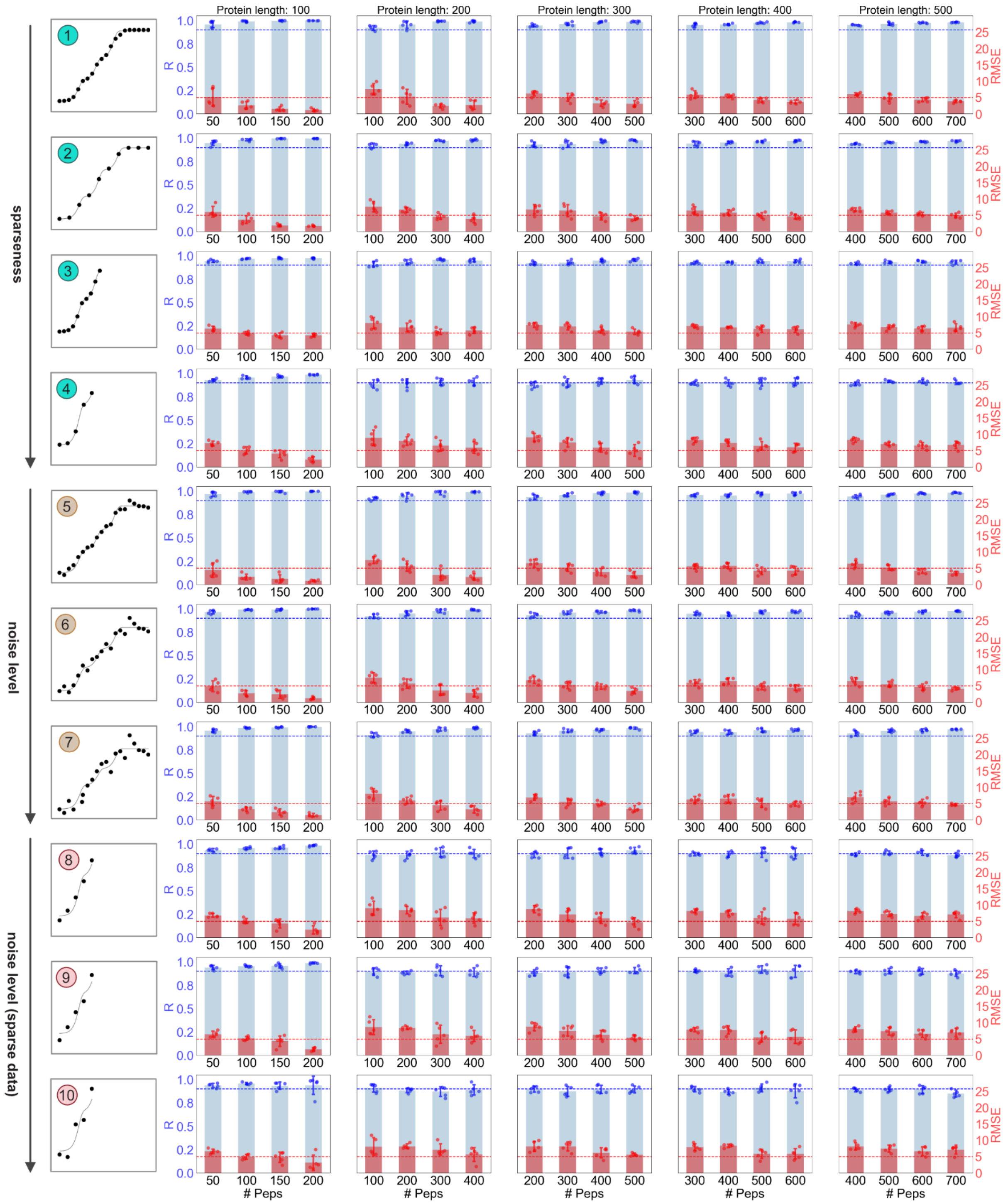

Supplementary Figure 5. Expanded data of Fig. 1b for PFNet(-CNN) predictions with confidence > 0.8.

PFNet(-CNN) performance on synthetic datasets with varying degrees of sparseness and noise across proteins of different sizes and peptide numbers, with R (blue) and RMSE (red). Data are shown as mean ± s.d. from five independently generated proteins per dataset.

a

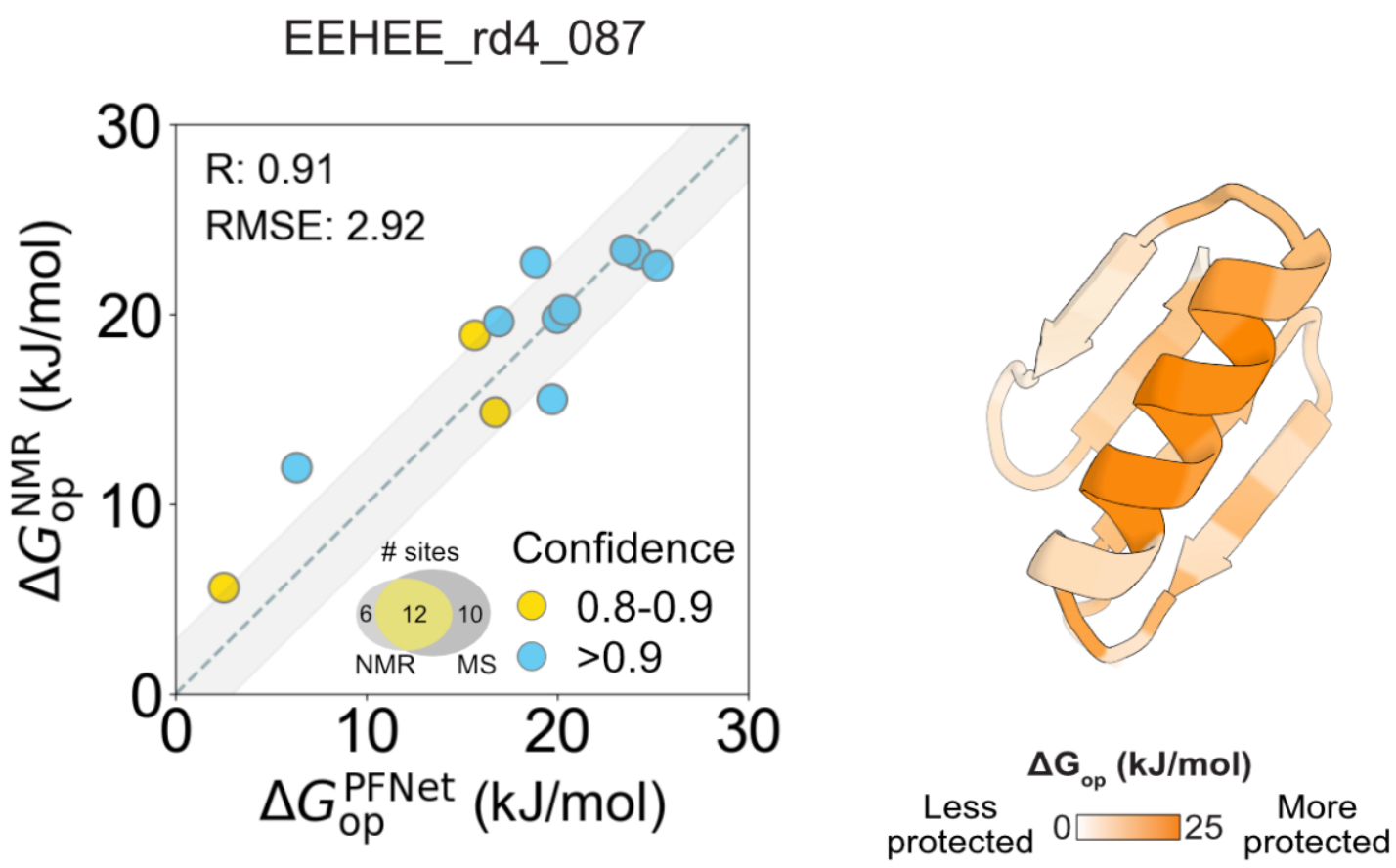

b

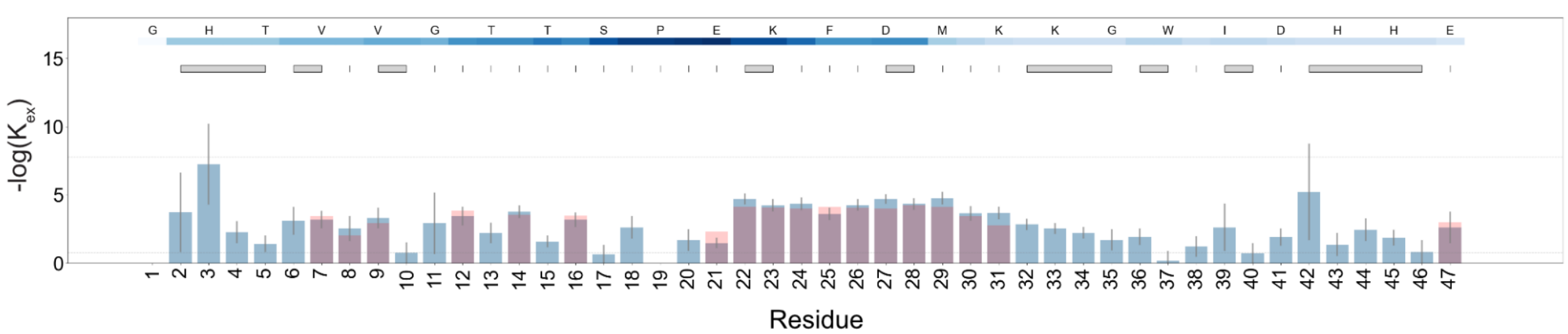

Supplementary Figure 6. Experimental benchmarking on the EEHEE_rd4_087 dataset.

a, PFNet-derived ΔG_{op} values mapped onto the AlphaFold3 predicted structure of HHH_rd4_0557. The Venn diagram shows the number of single-residue sites resolved by each technique/study. **b.** Residue-by-residue comparisons of NMR-derived and PFNet-predicted exchange rates along the sequence, accompanied by standard deviations calculated from the PFNet confidence score.

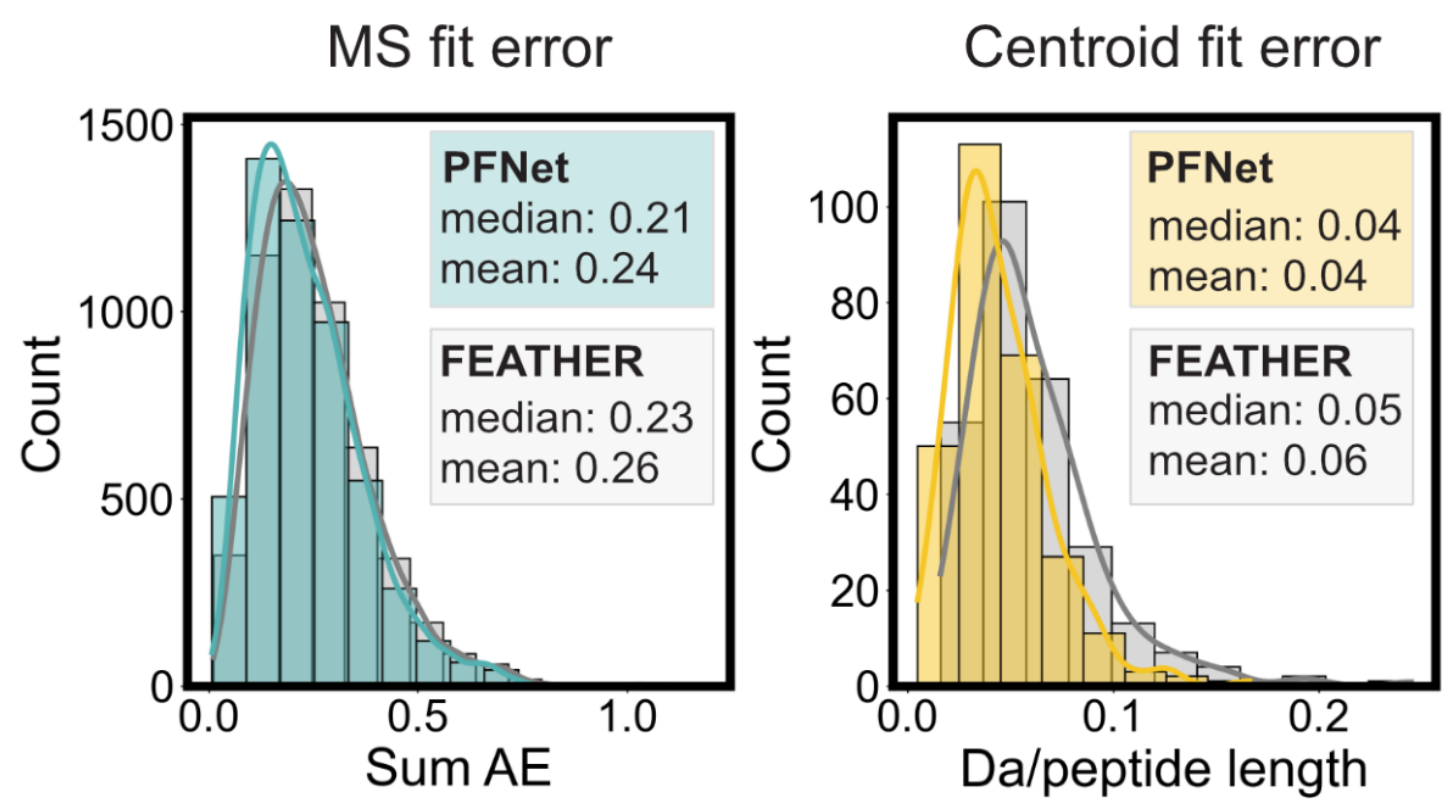

Supplementary Figure 7. Comparison of PFNet and FEATHER fitting errors for the EEHEE_rd4_087 dataset.

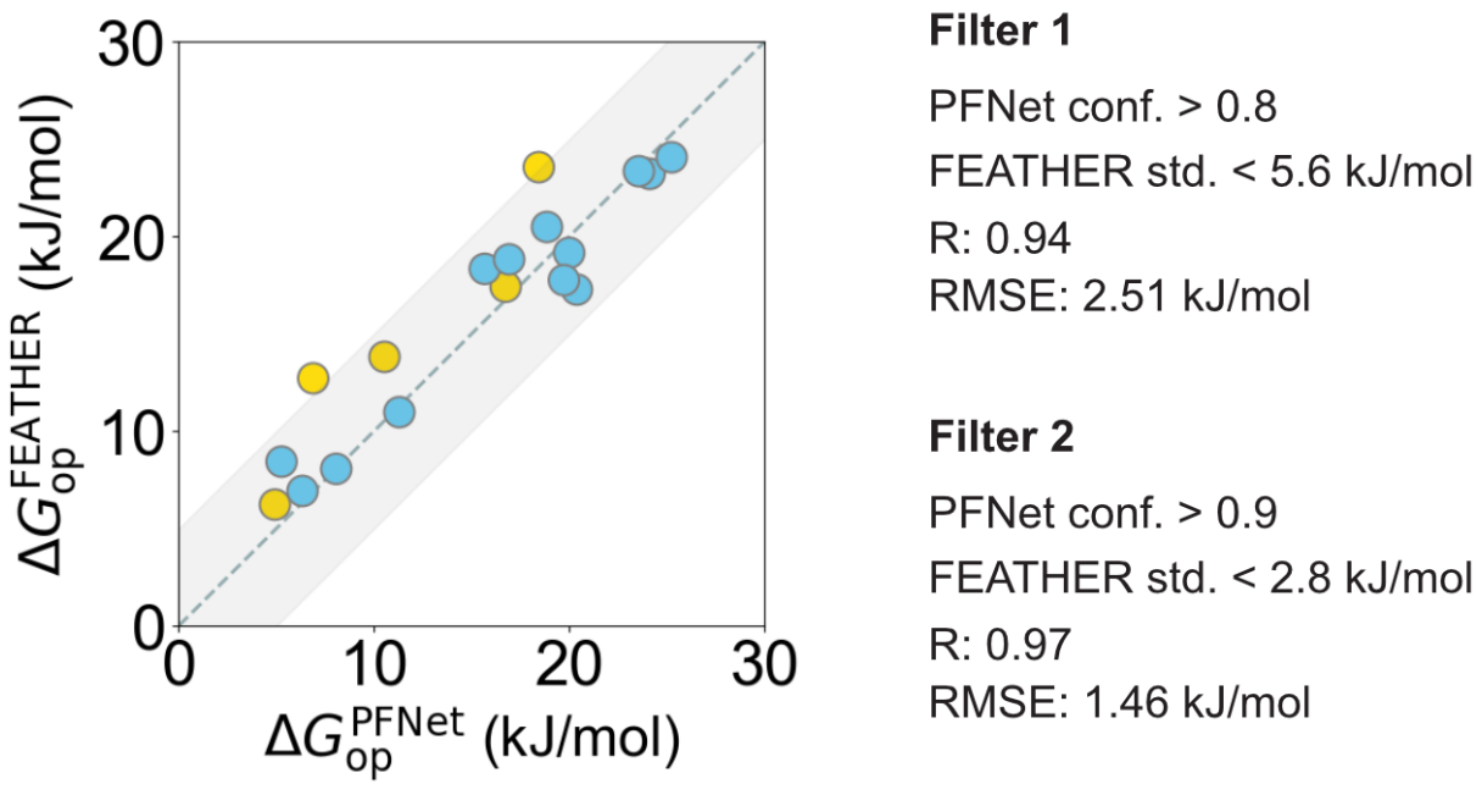

Supplementary Figure 8. Comparison of PFNet and FEATHER ΔG_{op} for the EEHEE_rd4_087 dataset.

R and RMSE were calculated at two different thresholds.

a

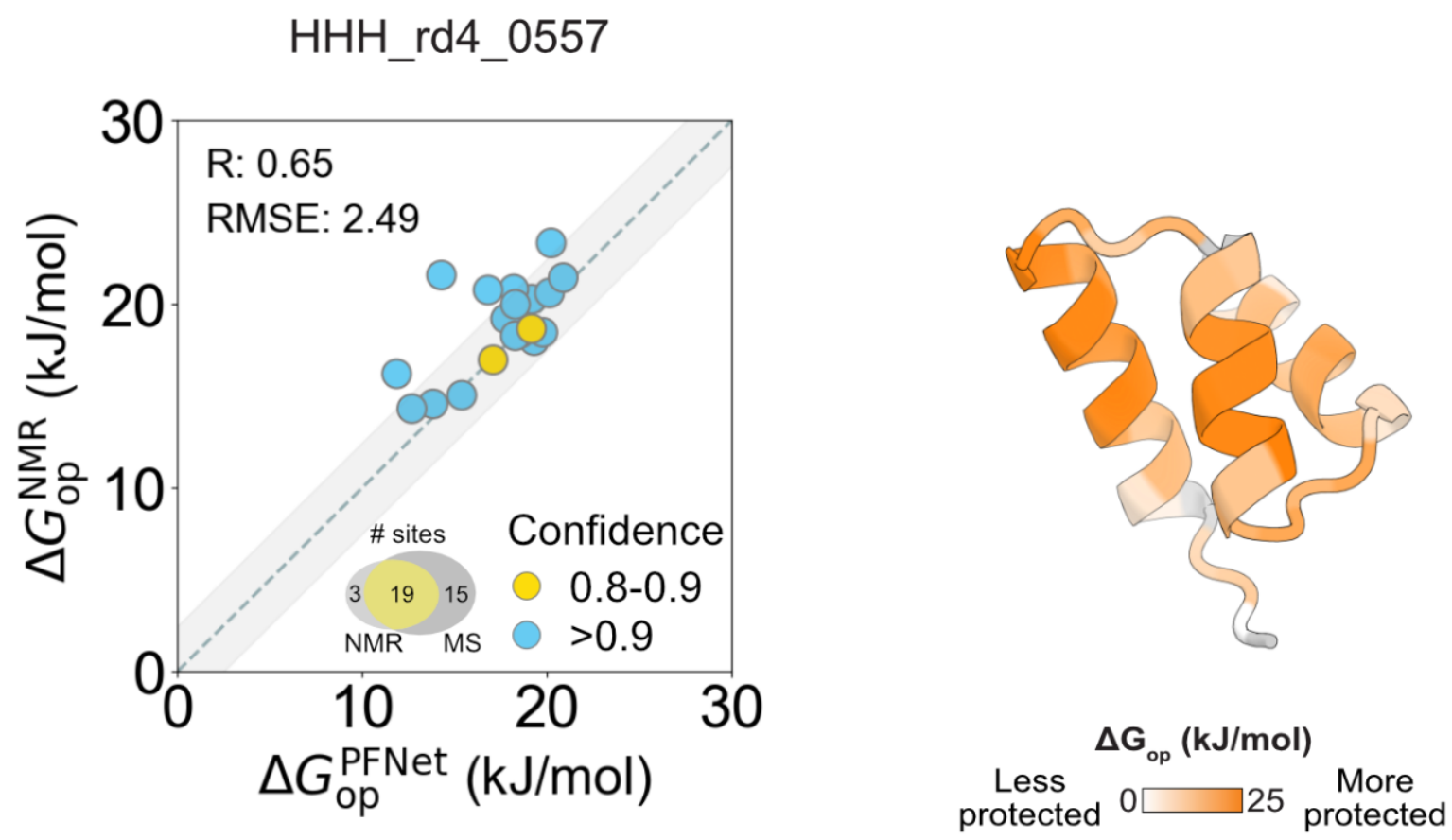

b

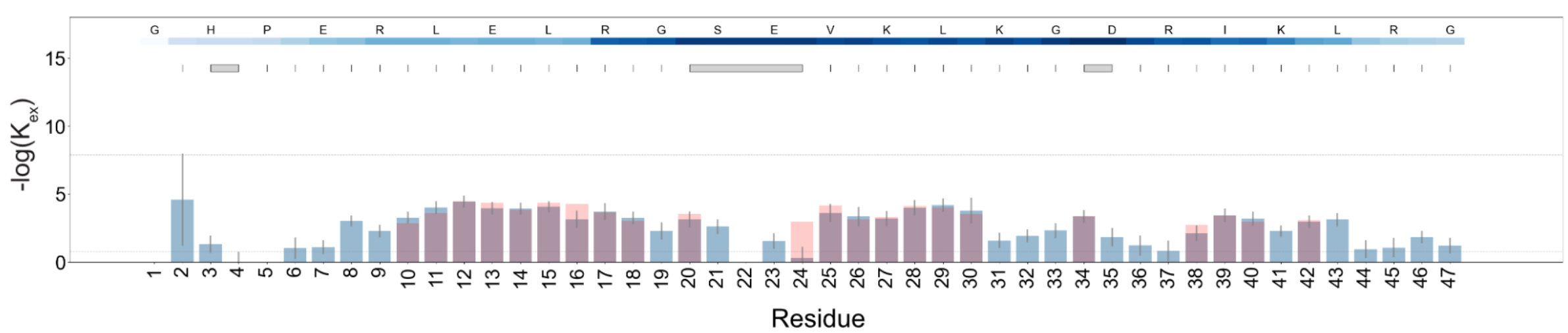

Supplementary Figure 9. Experimental benchmarking on the HHH_rd4_0557 dataset.

a, PFNet-derived ΔG_{op} values mapped onto the AlphaFold3 predicted structure of HHH_rd4_0557. The Venn diagram shows the number of single-residue sites resolved by each technique/study. **b.** Residue-by-residue comparisons of NMR-derived and PFNet-predicted exchange rates along the sequence, accompanied by standard deviations calculated from the PFNet confidence scores.

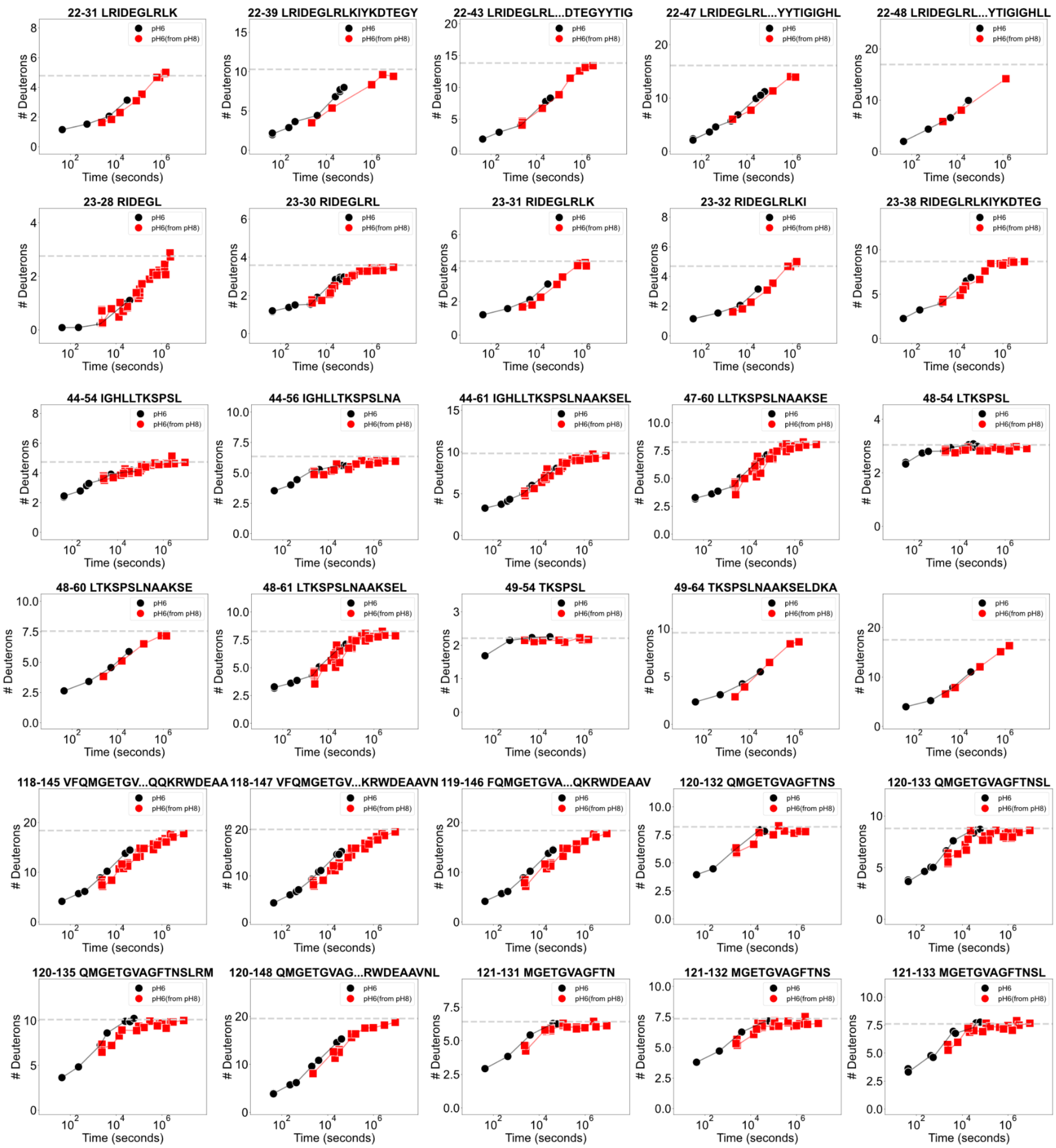

Supplementary Figure 10. Expanded exchange time window by pH conversion for T4L.

Representative peptide uptake plots from HX-MS experiments comparing direct measurements at pH 6.0 with values converted from pH 8.0, illustrating the broadened exchange time coverage achieved through pH conversion.

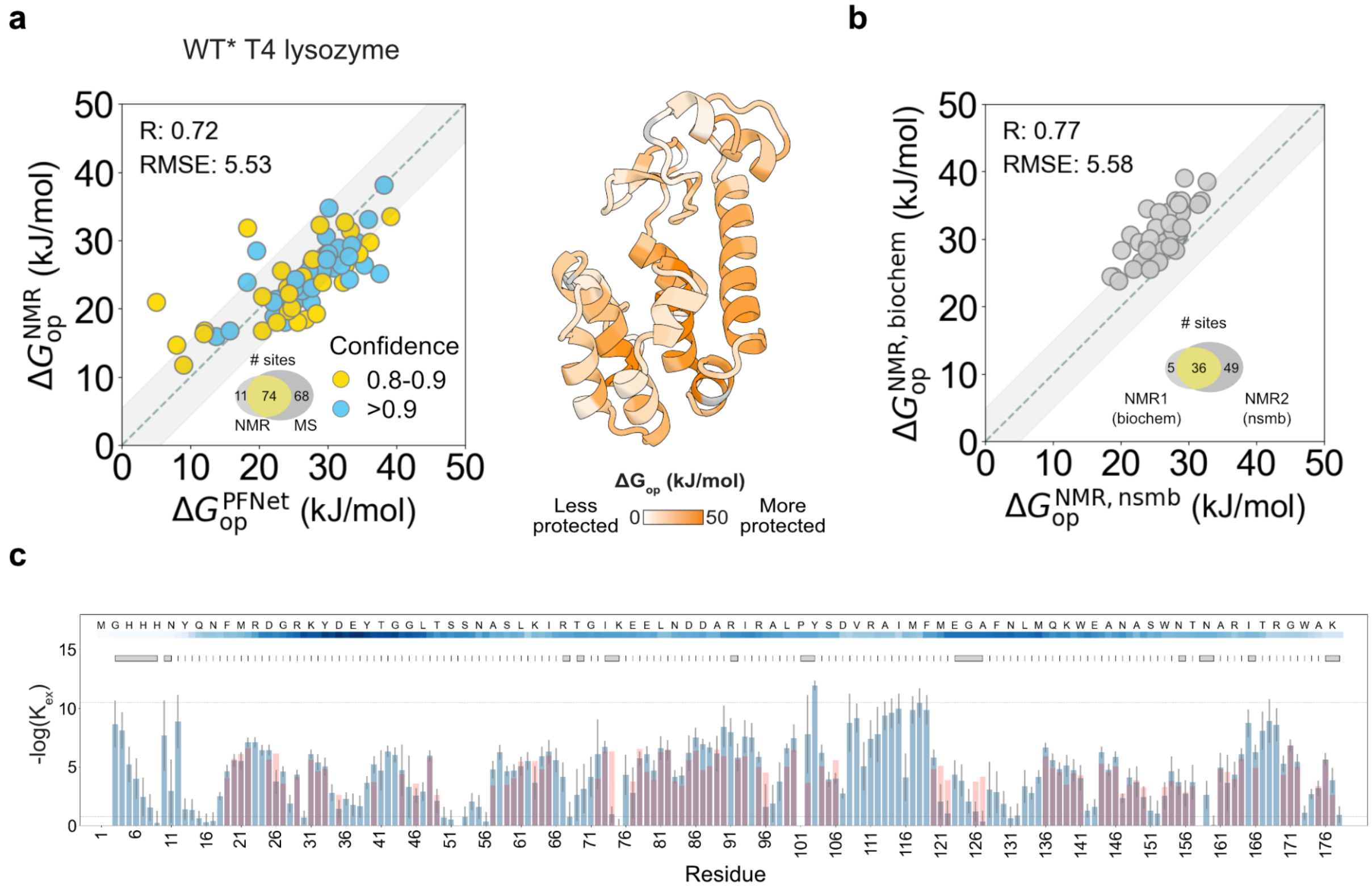

Supplementary Figure 11. Experimental benchmarking on the T4L dataset.

a, PFNet-derived ΔG_{op} values mapped onto the crystal structure of T4L (PDB: 5VNR). **b,** Comparison of ΔG_{op} values obtained from two independent HX-NMR studies: NMR1[1] and NMR2.[2] The NMR1 dataset was measured at 20 °C over a range of pH (read) values from 3.53 to 7.76, whereas the NMR2 dataset was measured at pH (read) 5.6 and 25 °C. The Venn diagram shows the number of single-residue sites resolved by each technique/study. **c.** Residue-by-residue comparisons of NMR-derived and PFNet-predicted exchange rates along the sequence, accompanied by standard deviations calculated from the PFNet confidence scores.

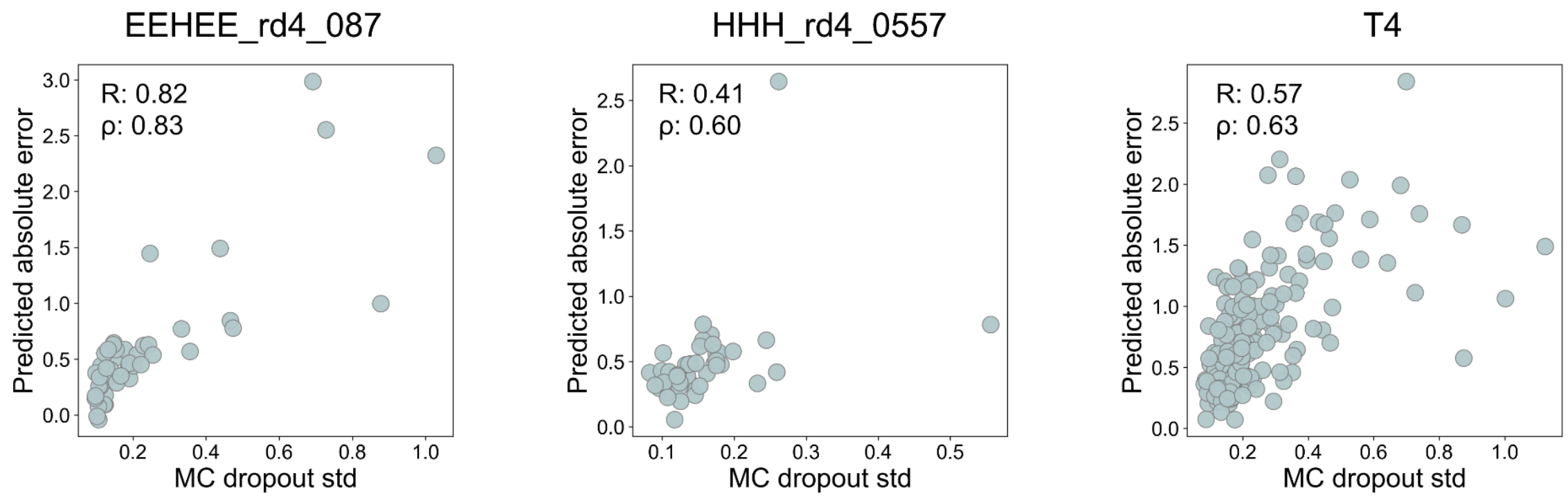

Supplementary Figure 12. Correlation between MC dropout standard deviation and the predicted absolute error from confidence for three experimental benchmark datasets.

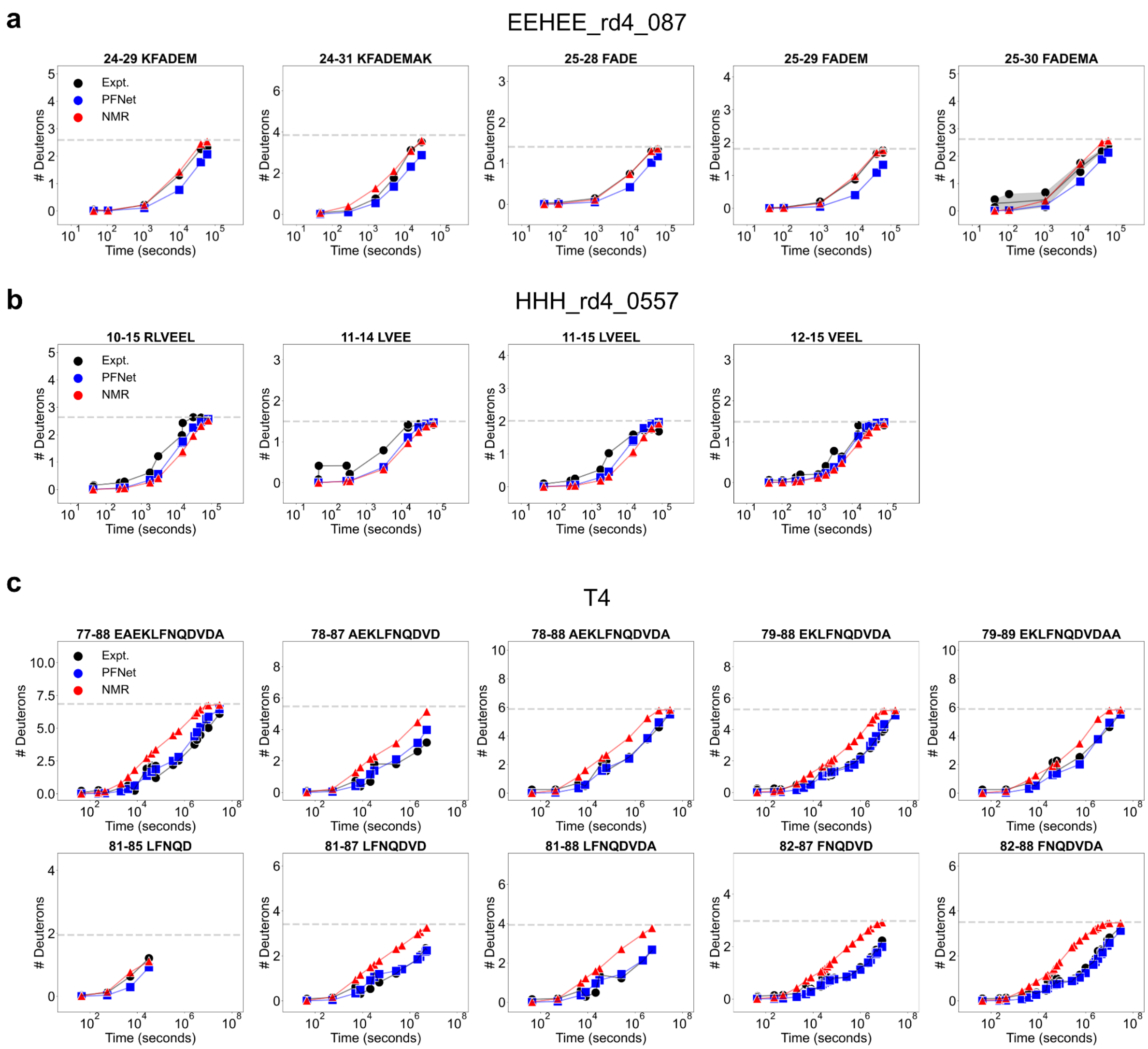

Supplementary Figure 13. Comparison of reconstructed uptake plots generated from exchange rates derived by HX-MS-PFNet or HX-NMR.

These regions represent peptide sequences that are fully covered by residue-resolved NMR values.

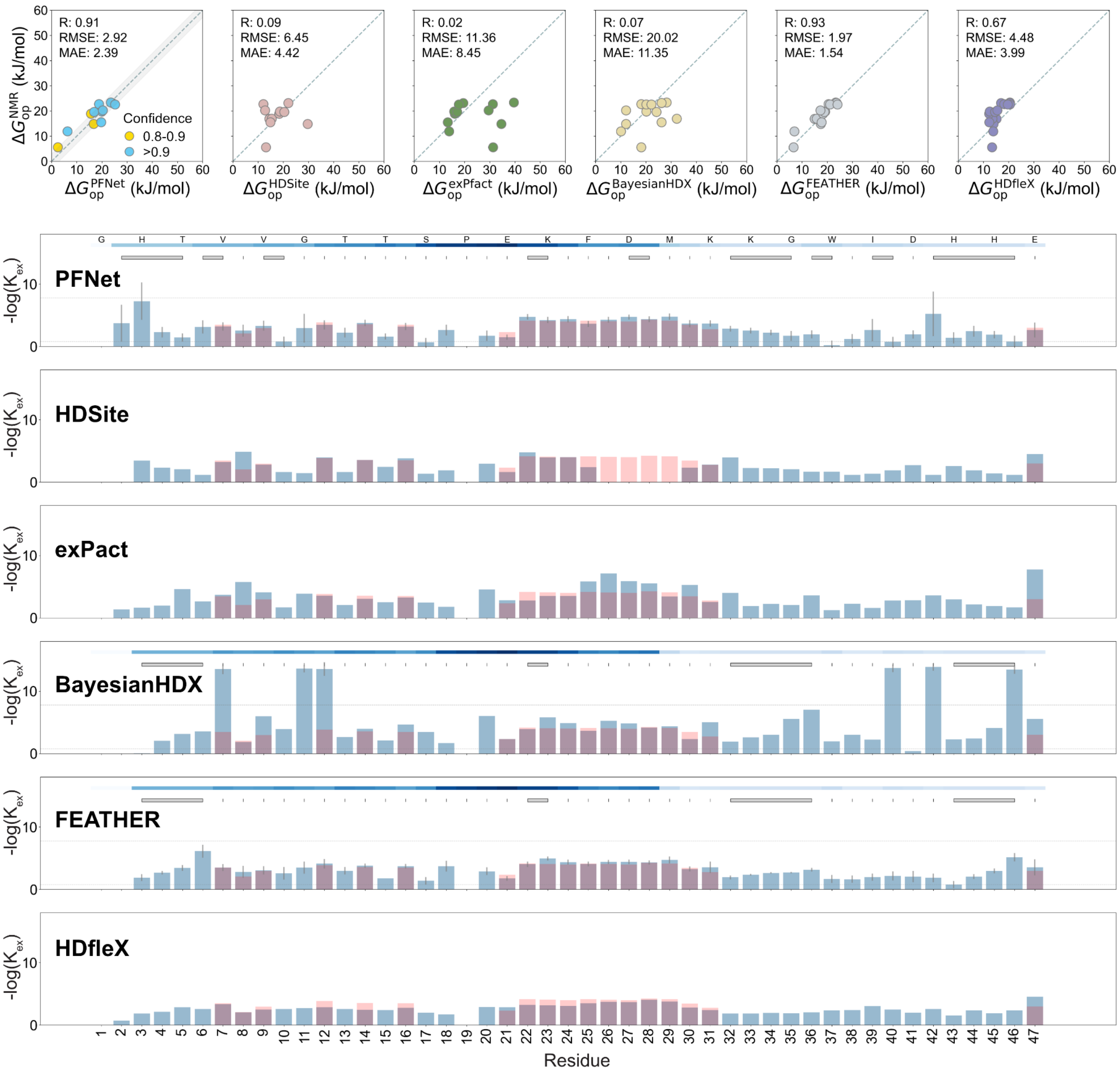

Supplementary Figure 14. Experimental HX-NMR benchmark comparison of PFNet with other methods on the EEHEE_rd4_087 dataset.

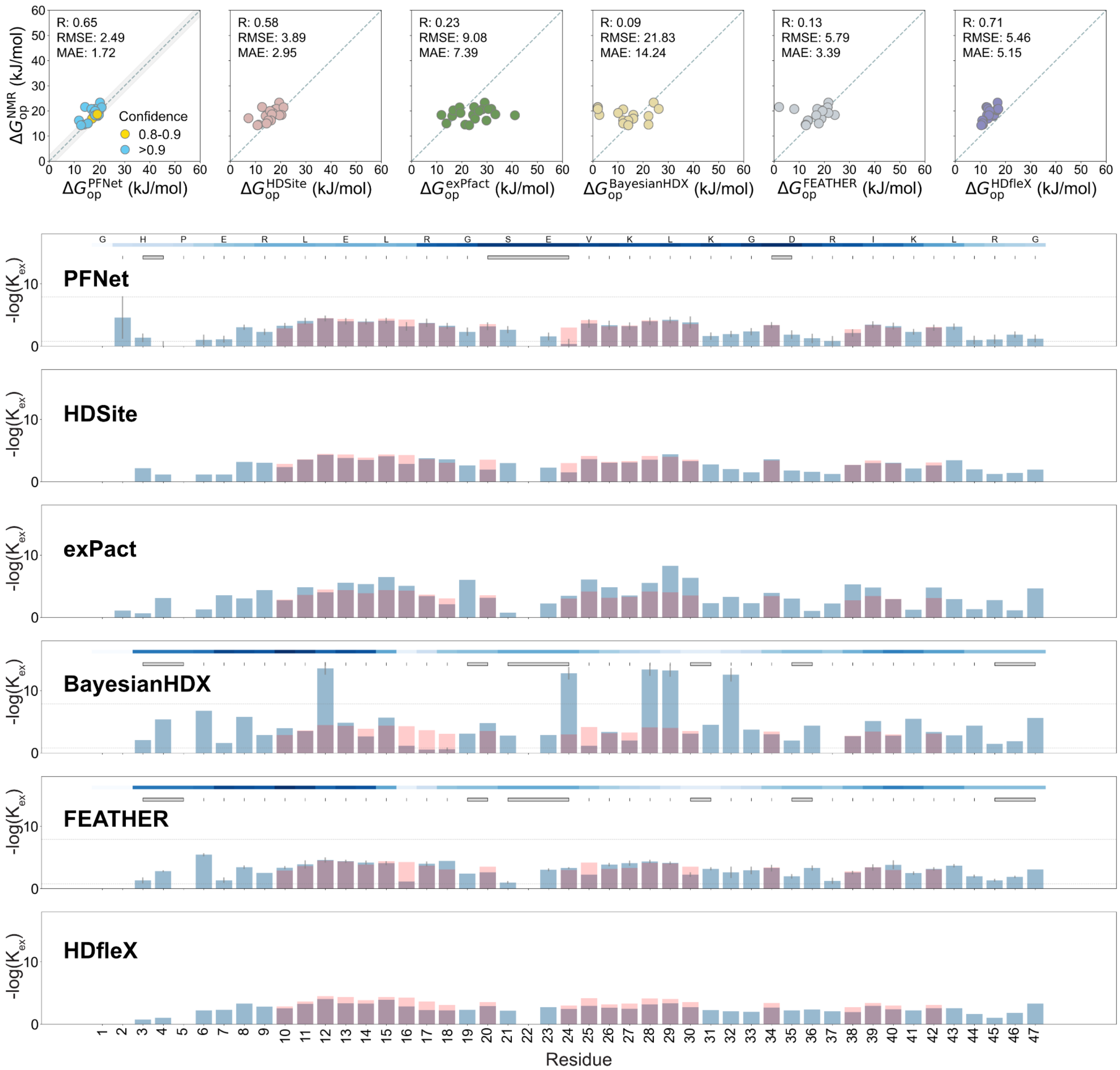

Supplementary Figure 15. Experimental HX-NMR benchmark comparison of PFNet with other methods on the HHH_rd4_0557 dataset.

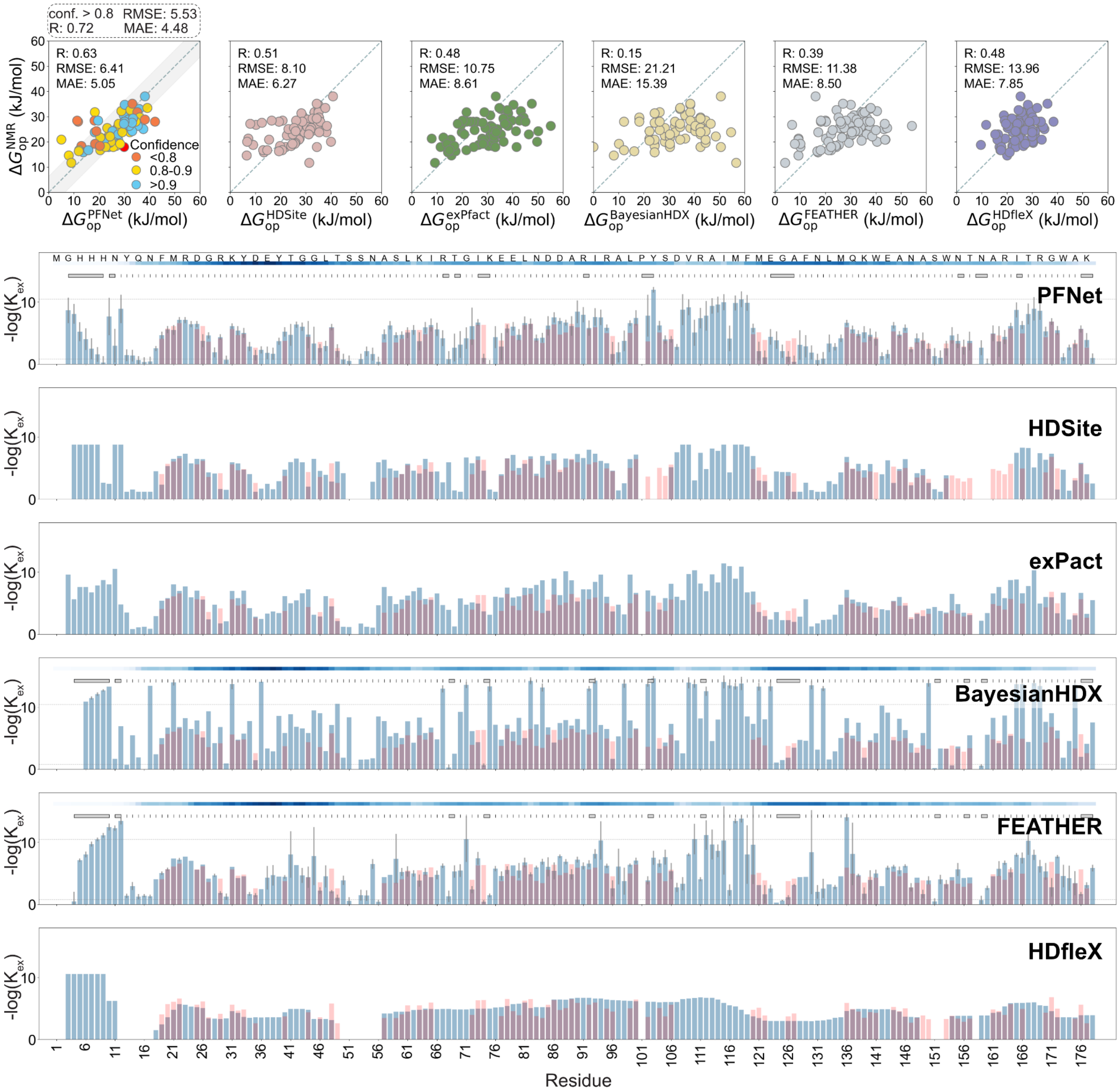

Supplementary Figure 16. Experimental HX-NMR benchmark comparison of PFNet with other methods on the T4L dataset.

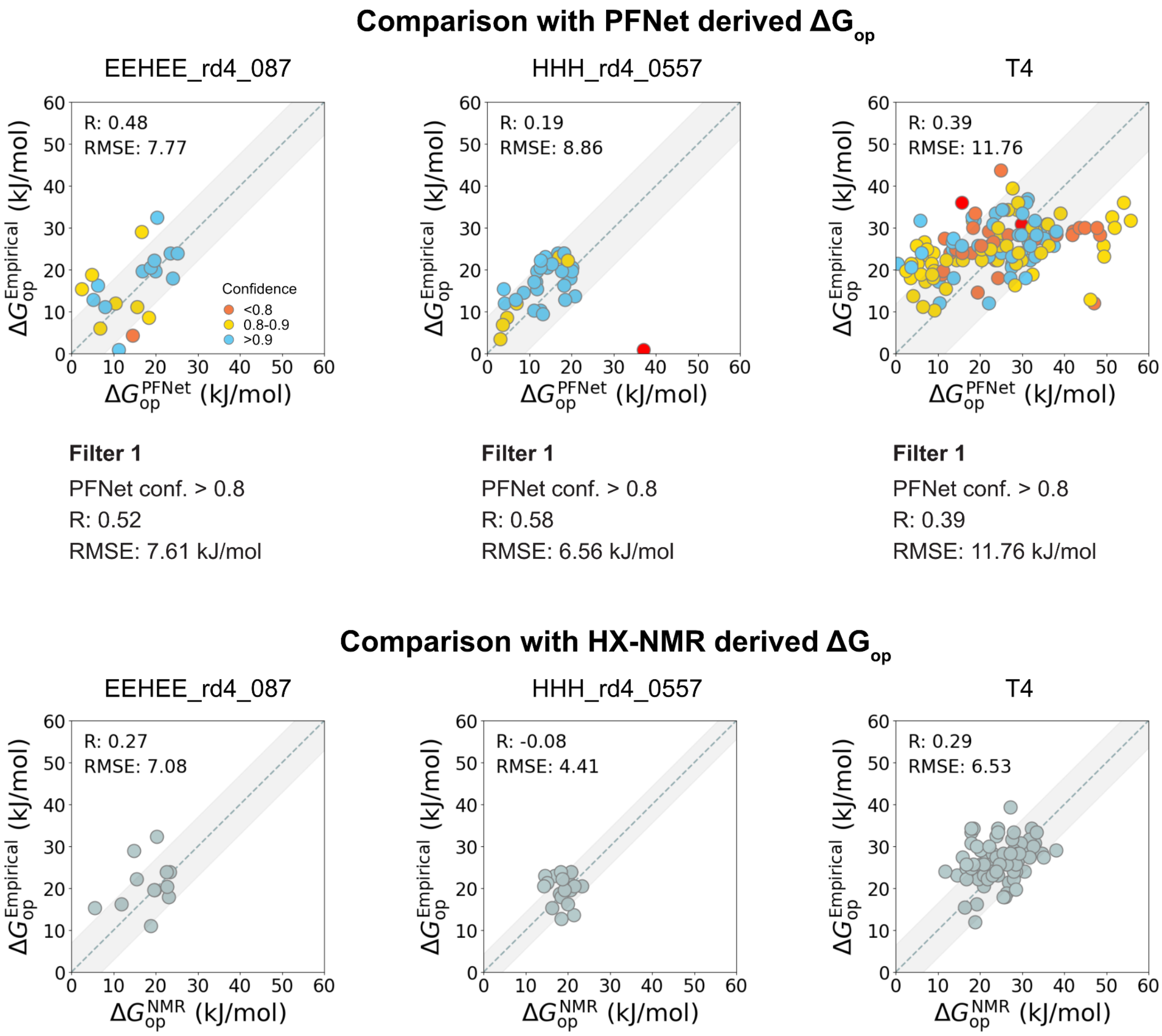

Supplementary Figure 17. Comparison of PFNet-derived and HX-NMR-derived ΔGop values with estimates from the Best/Vendruscolo empirical model.[3]

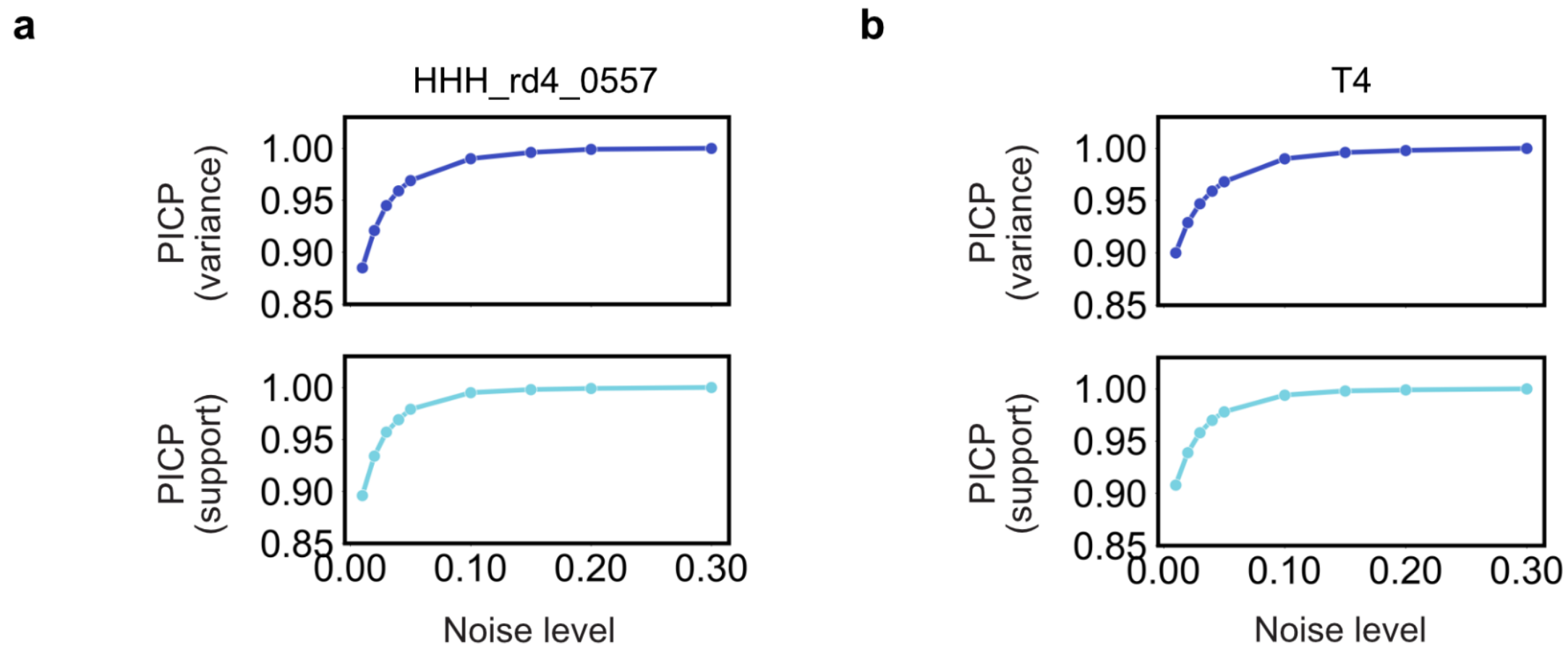

Supplementary Figure 18. PICP analysis for HHH_rd4_0557 and T4L HX-MS datasets.

The PICP analysis estimates the equivalent noise levels corresponding to the synthetic benchmarks.

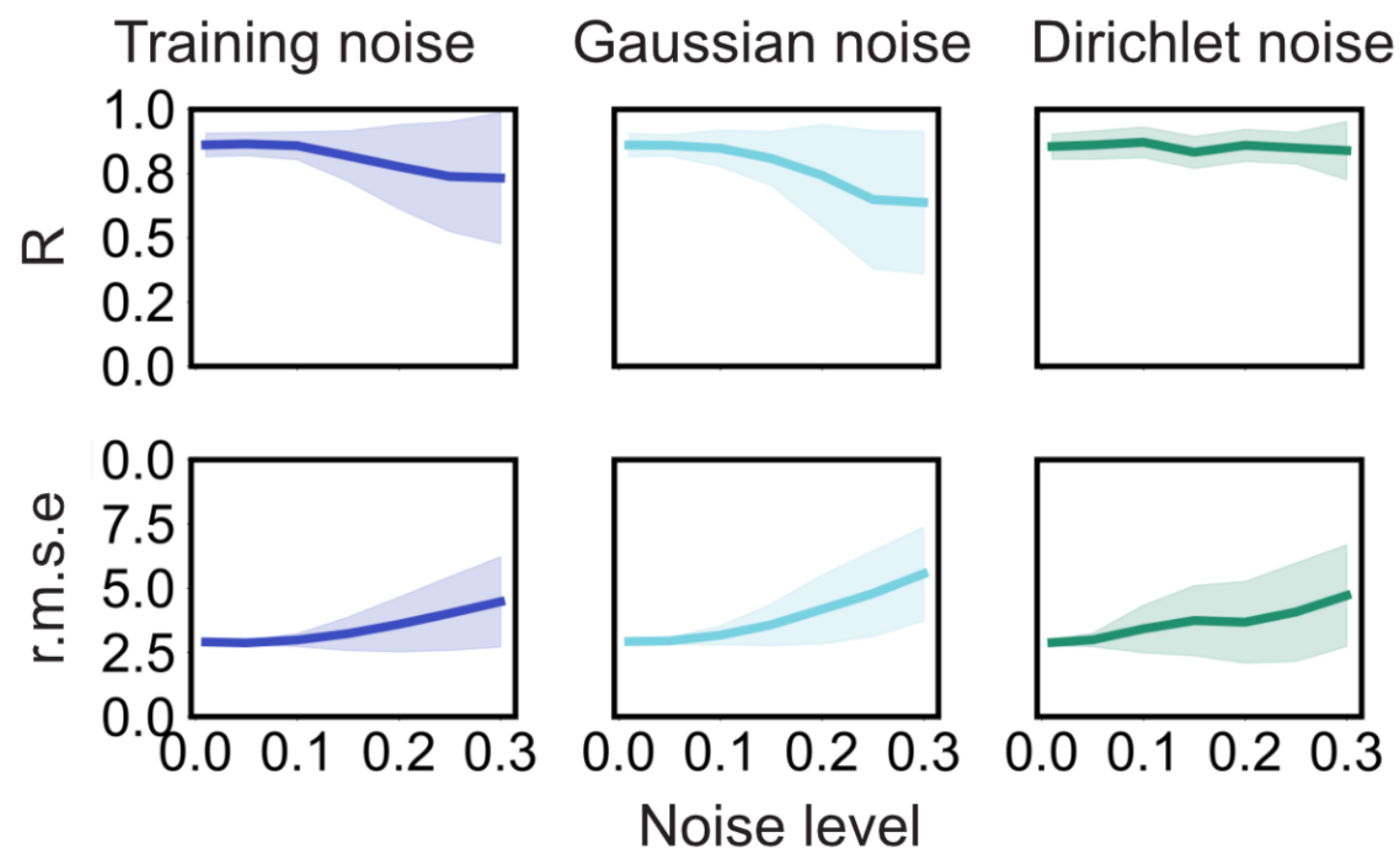

Supplementary Figure 19. PFNet robustness to noise injections in the EEHEE_rd4_087 dataset.

PFNet performance on the EEHEE_rd4_087 dataset incorporating additive training, Gaussian, and Dirichlet noise. The shaded area indicates the variation across 20 independent runs.

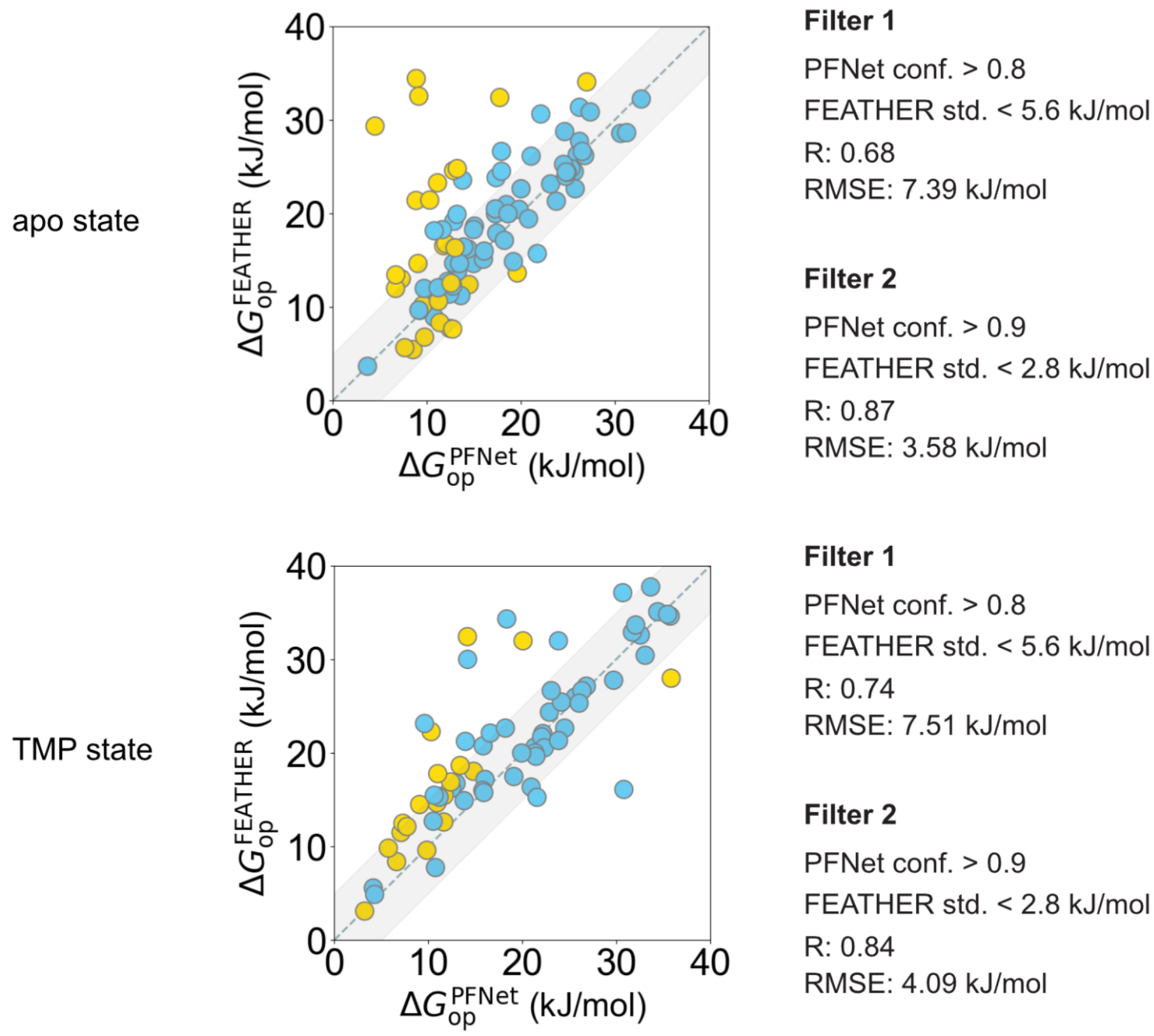

Supplementary Figure 20. Comparison of PFNet and FEATHER ΔG_{op} for apo and TMP-ecDHFR.

R and RMSE were calculated at two different thresholds.

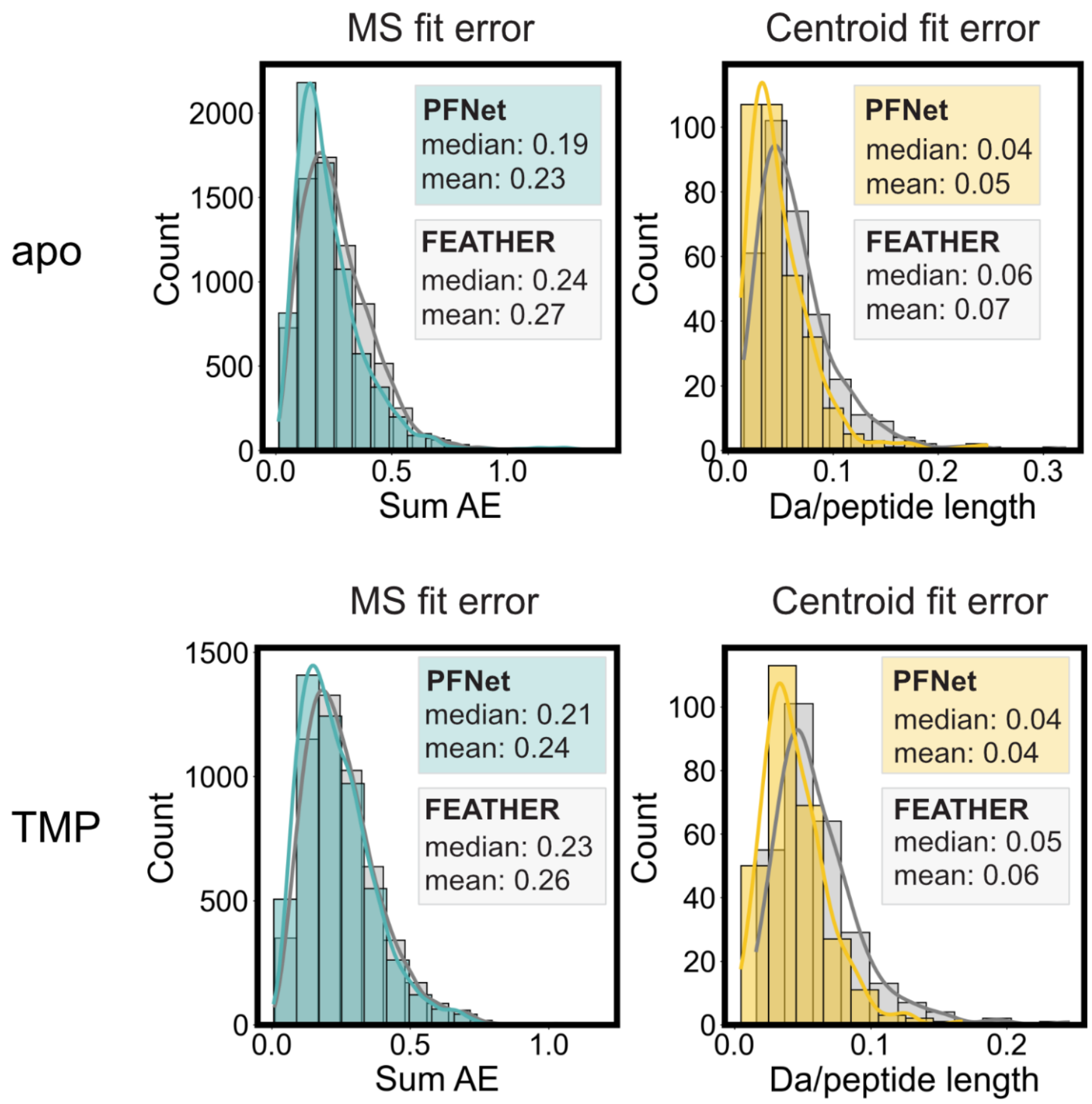

Supplementary Figure 21. Comparison of PFNet and FEATHER fitting errors on ecDHFR.

An expanded version of Fig. 2b showing detailed statistical comparisons of fitting errors between PFNet and FEATHER.

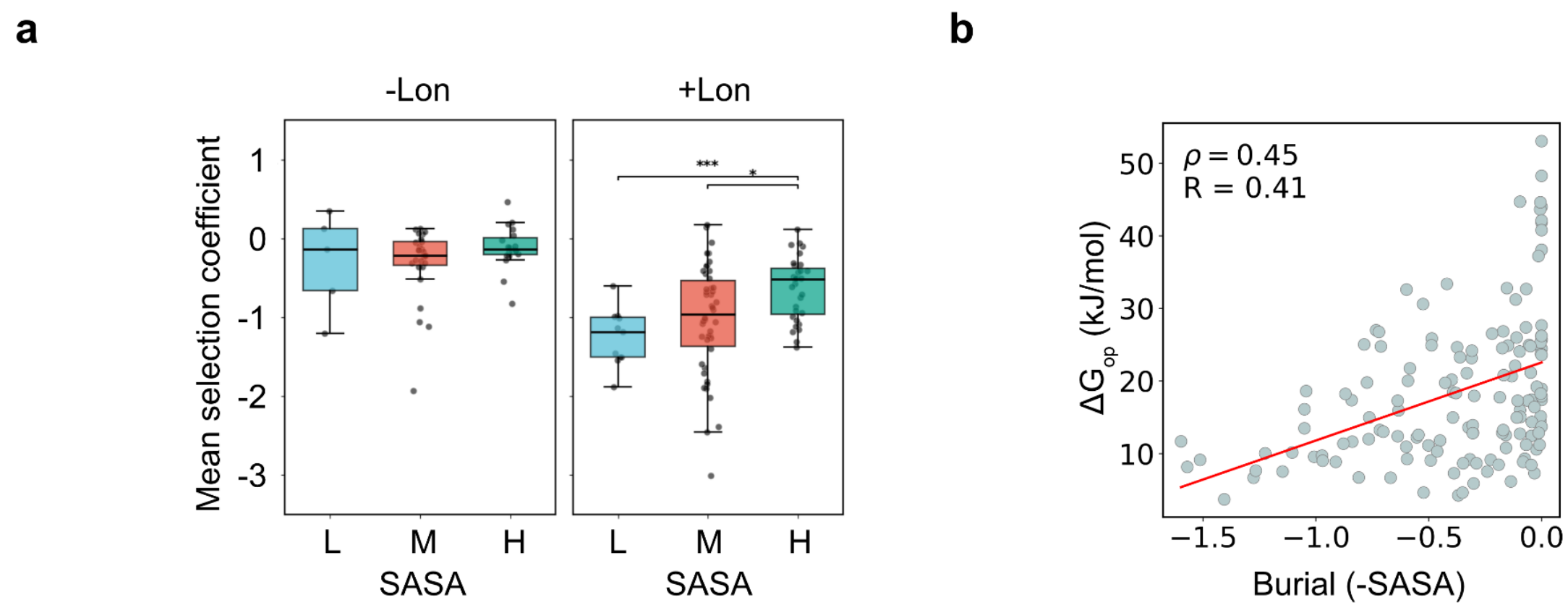

Supplementary Figure 22. DMS selection coefficients across SASA groups.

a. Distribution of selection coefficients across three solvent accessible surface area (SASA) groups in the absence or presence of Lon protease in DMS,[4] sorted by SASA as defined by the 25th and 75th percentiles: Low (< 0.06 nm²), Medium (0.06–0.60 nm²), and High (> 0.60 nm²). Significant pairwise differences determined by the Mann–Whitney U test are indicated by "*"*($p <$ 0.05)*, "***"*($p < 0.01$)*. **b.** Correlation between residue burial, quantified as negative SASA, and $\Delta G_{op.}$

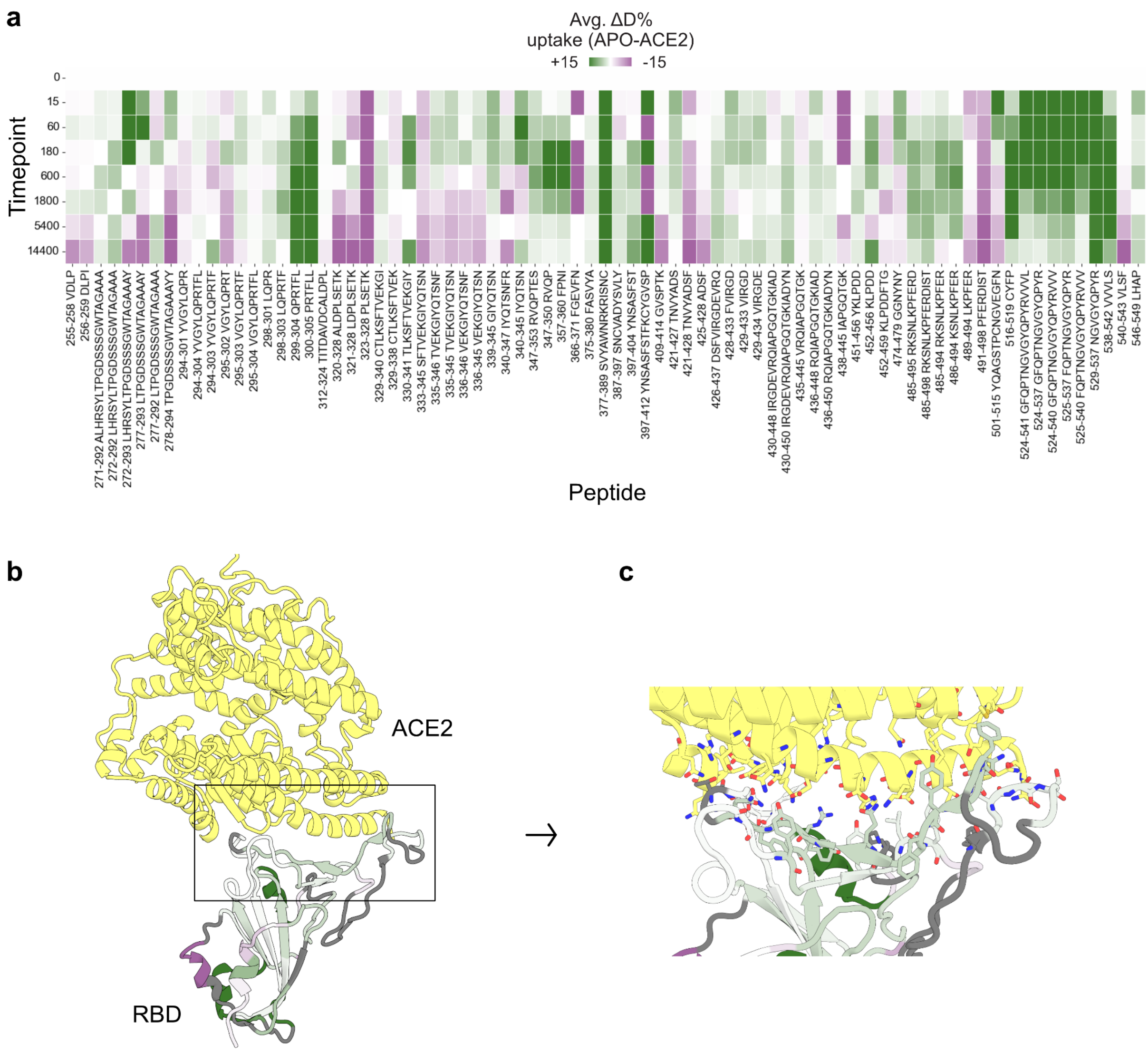

Supplementary Figure 23. Peptide level analysis of SARS-CoV-2 spike protein datasets

Colors indicate average deuterium uptake across the timecourse, normalized by peptide length (average ΔD%).[5] **a.** Heat map showing all the peptides colored by ΔD% of each timepoint. **b-c.** structural models (PDB: 8H3D, 6M0J) painted by ΔD%.

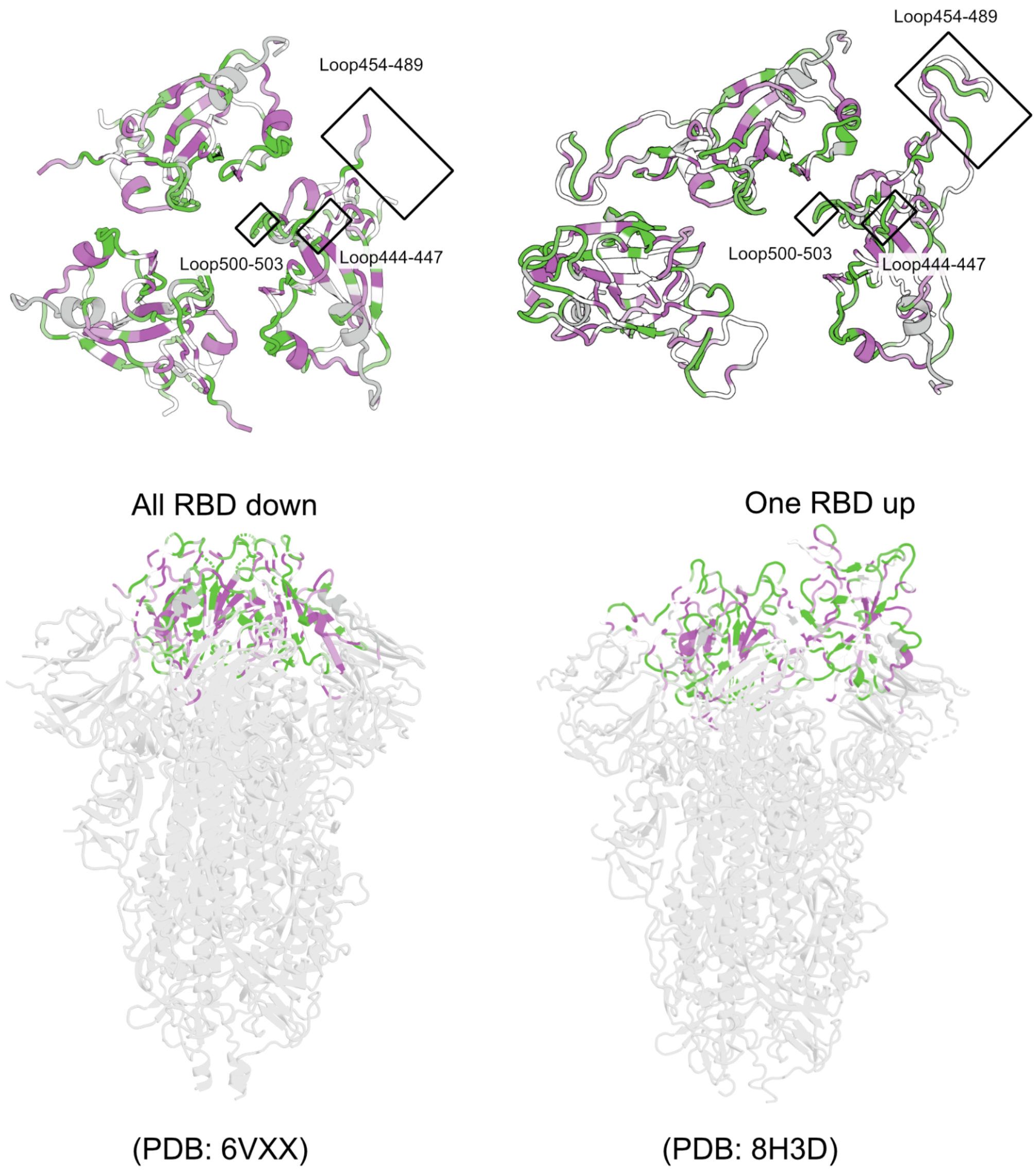

Supplementary Figure 24. PFNet-derived $\Delta\Delta G_{op(apo\text{-}ACE2)}$ values mapped on the spike structure.

Three loop regions become rigidified upon the ACE2 binding-driven population shift in the spike protein, as shown in the all-RBD-down (PDB: 6VXX, left) and one RBD-up conformations (PDB: 8H3D, right).

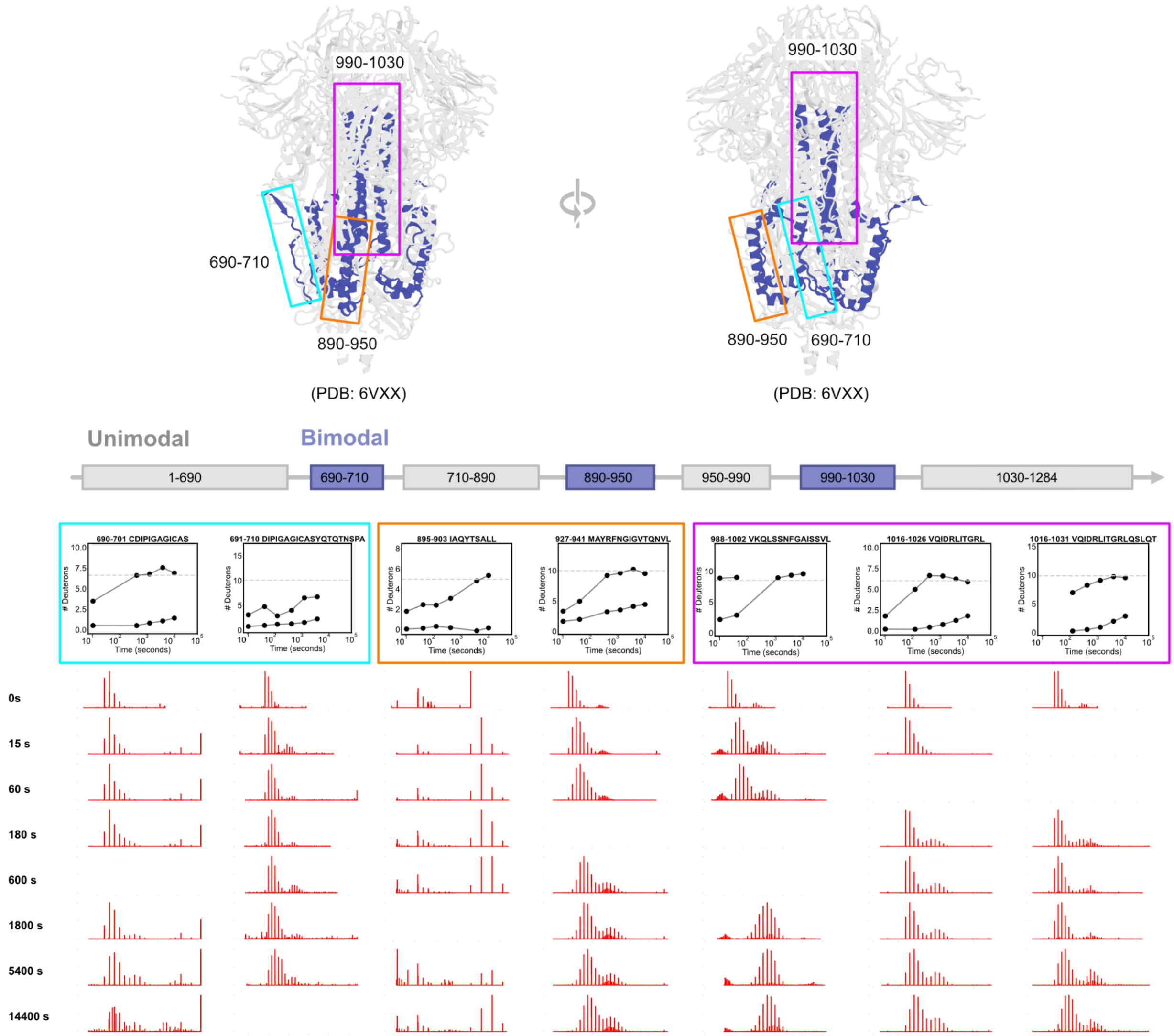

Supplementary Figure 25. Bimodal regions of the SARS-CoV-2 spike protein.

Representative peptides shown for each region, alongside the raw isotopic mass envelopes.

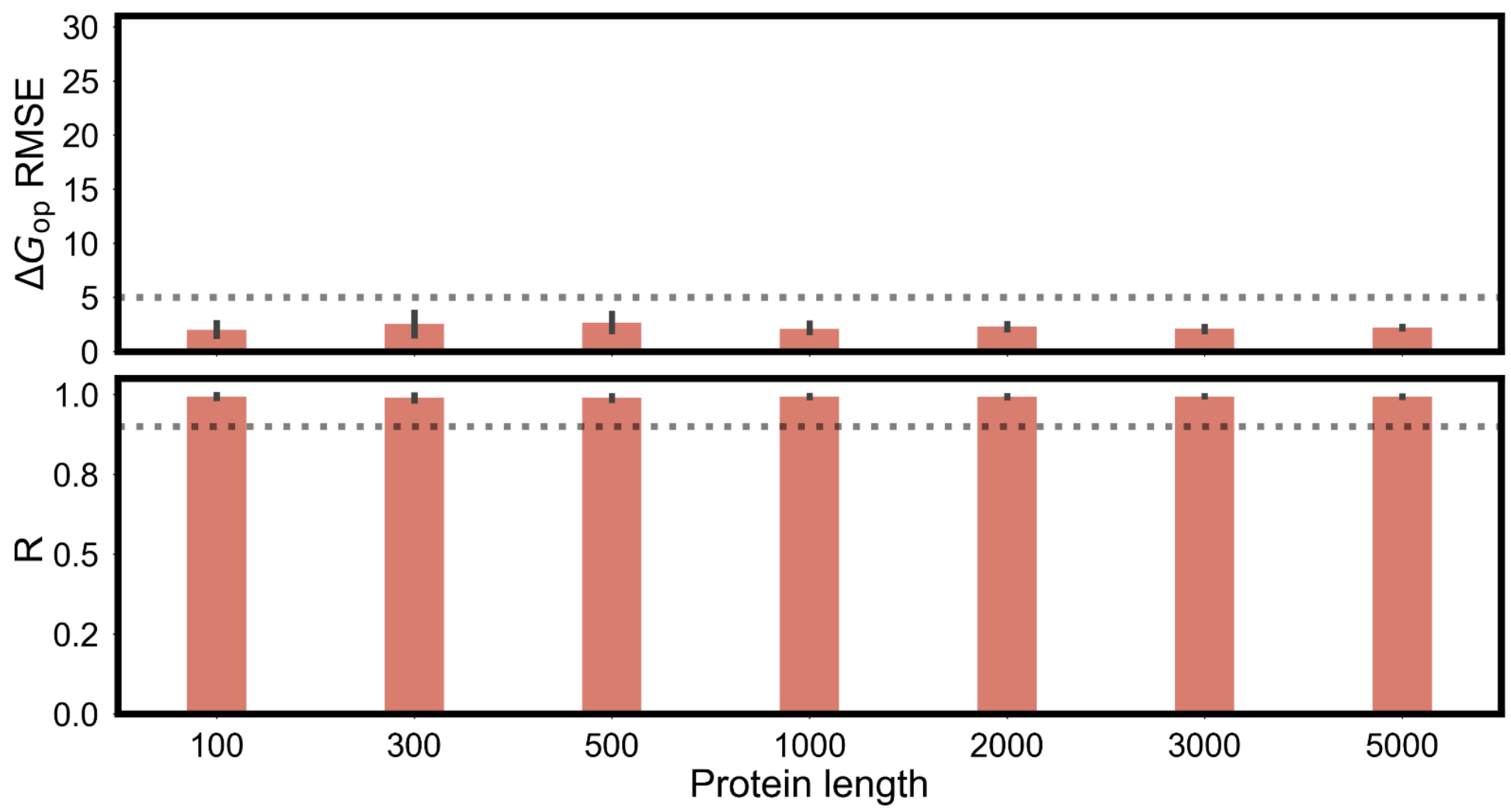

Supplementary Figure 26. PFNet scales to large proteins without loss of predictive accuracy.

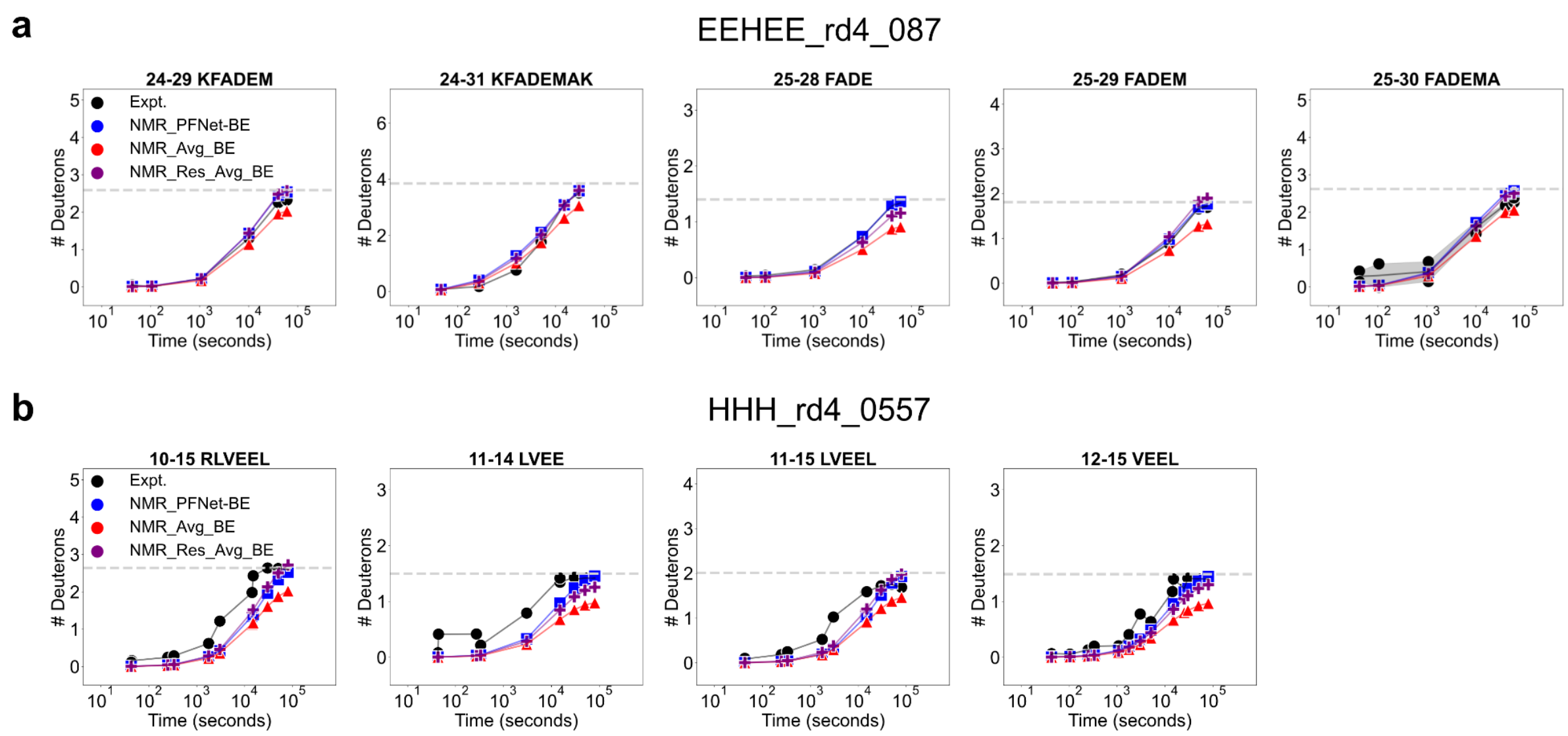

Supplementary Figure 27. Comparison of reconstructed uptake plots under three different back-exchange correction methods.

Exchange rates were derived from HX-NMR data for peptide sequences fully covered by residue-resolved NMR values.

2. Supplementary tables

Supplementary Table 1. Synthetic benchmarking datasets

Dataset index	Data quality	log(P) range	# timepoints	Time window (s)	Noise level
1	perfect	2-12	20	1e-6 to 1e12	0
2	sparse	2-12	10	1e1 to 1e12	0
3	tp_1e6	2-12	10	1e1 to 1e6	0
4	sparse_tp_1e5	2-12	5	1e1 to 1e5	0
5	perfect_noise10	2-12	20	1e1 to 1e12	0.1
6	perfect_noise20	2-12	20	1e1 to 1e12	0.2
7	perfect_noise30	2-12	20	1e1 to 1e12	0.3
8	sparse_tp_1e5_noise10	2-12	5	1e1 to 1e5	0.1
9	sparse_tp_1e5_noise20	2-12	5	1e1 to 1e5	0.2
10	sparse_tp_1e5_noise30	2-12	5	1e1 to 1e5	0.3

Supplementary Table 2. HX experiment summary table for EEHEE_rd4_087.

This dataset was previously reported[6] but is included here for convenience. All experiments were performed with the KEEP setting in PIGEON. Abbreviations: FP, immobilized protease type XIII. Nep, nepenthesin 2. AP, alanyl aminopeptidase.

Dataset	**EEHEE_rd4_087 Rep1**	**EEHEE_rd4_087 Rep2**	**EEHEE_rd4_087 Rep3**
Protein states	APO	APO	APO
Date	08/19/24	09/10/24	10/23/24
Protease column	Nep/pepsin	AP/pepsin	FP/pepsin
Reaction details	25mM MES, pH 6.0, temperature 20 °C, final D_2O concentration 86.3%		
Time course (s)	4.2e1-6.0e4	4.6e1-3.0e4	4.4e1-2.5e4
# of time points	6	6	5
Control samples	Maximally-labeled sample (full-D)		
Back-exchange (mean / IQR)	0.23/0.07	0.29/0.13	0.20/0.12
# of peptides	35	21	46
Sequence coverage	0.96	0.96	0.96
Average peptide length / Redundancy	1.3	2.2	1.0

Supplementary Table 3. ΔG_{op} values for EEHEE_rd4_087.

Res. name	Res. number	Apo	pStd Dev	Confidence	Single resolve
GLY	1				
SER	2	31.98	3.08	0.64	FALSE
HIS	3	25.56	3.08	0.63	FALSE
MET	4	24.04	3.08	0.89	FALSE
THR	5	20.09	3.08	0.92	FALSE
GLN	6	18.4	3.24	0.86	FALSE
VAL	7	14.14	3.24	0.91	FALSE
HIS	8	16.73	4.81	0.87	TRUE
VAL	9	11.03	3.99	0.9	FALSE
ASP	10	10.56	3.99	0.89	FALSE
GLY	11	14.6	12.37	0.7	TRUE
VAL	12	15.66	3.56	0.9	TRUE
THR	13	10.53	3.82	0.9	TRUE
TYR	14	19.99	2.27	0.94	TRUE
THR	15	8.06	2.1	0.94	TRUE
PHE	16	16.91	2.63	0.93	TRUE
SER	17	5.26	3.5	0.91	TRUE
ASN	18	18.43	4.4	0.88	TRUE
PRO	19				
GLU	20	6.88	4.14	0.89	TRUE
GLU	21	6.34	1.8	0.95	TRUE
ALA	22	23.68	2.17	0.95	FALSE
LYS	23	24.17	2.17	0.94	FALSE
LYS	24	24.11	2.32	0.94	TRUE
PHE	25	18.85	2.12	0.94	TRUE
ALA	26	23.55	2.04	0.94	TRUE
ASP	27	25.24	2.05	0.95	FALSE
GLU	28	23.16	2.05	0.94	FALSE
MET	29	25.23	2.26	0.94	TRUE

ALA	30	20.38	2.65	0.93	TRUE
LYS	31	19.72	2.29	0.94	TRUE
ARG	32	13.43	4.03	0.94	FALSE
LYS	33	13.32	4.03	0.95	FALSE
GLY	34	12.81	4.03	0.94	FALSE
GLY	35	13.09	4.03	0.89	FALSE
THR	36	5.76	3.76	0.92	FALSE
TRP	37	4.02	3.76	0.9	FALSE
GLU	38	4.92	3.88	0.9	TRUE
ILE	39	3.86	3.96	0.76	FALSE
LYS	40	7.15	3.96	0.89	FALSE
ASP	41	11.29	3.21	0.91	TRUE
GLY	42	11.34	4.49	0.57	FALSE
HIS	43	17.39	4.49	0.88	FALSE
ILE	44	12.55	4.49	0.88	FALSE
HIS	45	15.15	4.49	0.92	FALSE
VAL	46	12.72	4.49	0.88	FALSE
GLU	47	2.52	6.07	0.84	TRUE

Supplementary Table 4. HX experiment summary table for HHH_rd4_0557.

All experiments were performed with the KEEP setting in PIGEON. Abbreviations: FP, immobilized protease type XIII. Nep, nepenthesin 2. AP, alanyl aminopeptidase.

Dataset	**HHH_rd4_0557 Rep1**	**HHH_rd4_0557 Rep2**	**HHH_rd4_0557 Rep3**	**HHH_rd4_0557 Rep4**
Protein states	APO	APO	APO	APO
Date	09/01/24	10/23/24	08/20/24	01/18/25
Protease column	AP/pepsin	FP/pepsin	Nep/pepsin	Nepenthesin-1
Reaction details	25mM MES, pH 6.0, temperature 20 °C, final D_2O concentration 86.3%			
Time course (s)	4.4e1-3.0e4	4.4e1-1.4e4	4.3e1-2.5e4	4.3e1-8.0e4
# of time points	5	4	5	7
Control samples	Maximally-labeled sample (full-D)			
Back-exchange (mean / IQR)	0.16/0.09	0.19/0.07	0.15/0.10	0.19/0.11
# of peptides	41	47	64	34
Sequence coverage	0.96	0.96	0.96	0.96
Average peptide length / Redundancy	1.1	1.0	0.7	1.4

Supplementary Table 5. ΔG_{op} values for HHH_rd4_0557.

Res. name	Res. number	Apo	pStd Dev	Confidence	Single resolve
GLY	1				
SER	2	37.15	18.63	0.59	TRUE
HIS	3	8.59	3.69	0.91	FALSE
MET	4	7.06	3.69	0.89	FALSE
PRO	5				
GLU	6	3.15	4.07	0.89	TRUE
GLU	7	4.18	2.57	0.93	TRUE
VAL	8	11.64	1.84	0.95	TRUE
ARG	9	11.88	2.24	0.94	TRUE
ARG	10	19.3	2.07	0.94	TRUE
LEU	11	19.77	2.34	0.94	TRUE
VAL	12	19.16	2.08	0.94	TRUE
GLU	13	20.21	2.27	0.94	TRUE
GLU	14	20.13	2.12	0.94	TRUE
LEU	15	18.19	1.96	0.95	TRUE
TYR	16	14.28	3.28	0.91	TRUE
ARG	17	20.87	3.17	0.91	TRUE
LYS	18	18.52	2.2	0.94	TRUE
GLY	19	12.68	3.21	0.91	TRUE
VAL	20	7.08	3.21	0.92	FALSE
SER	21	11.35	3.21	0.93	FALSE
PRO	22				
GLU	23	8.16	3.21	0.92	FALSE
GLN	24	9.68	3.21	0.89	FALSE
VAL	25	16.8	3.43	0.91	TRUE
LYS	26	17.07	3.63	0.9	TRUE
LYS	27	17.75	2.78	0.92	TRUE
ILE	28	18.28	2.88	0.92	TRUE
LEU	29	18.27	2.41	0.93	TRUE

LYS	30	19.16	4.94	0.87	TRUE
LYS	31	8.69	2.85	0.92	TRUE
GLN	32	11.16	2.38	0.93	TRUE
GLY	33	13.24	2.77	0.92	TRUE
ILE	34	10.73	2.82	0.94	FALSE
ASP	35	13.2	2.82	0.91	FALSE
GLU	36	4.73	3.79	0.9	TRUE
ARG	37	3.73	3.88	0.9	TRUE
GLU	38	11.86	2.88	0.92	TRUE
ILE	39	13.83	2.37	0.93	TRUE
GLU	40	15.38	2.6	0.93	TRUE
LYS	41	11.19	1.98	0.95	TRUE
VAL	42	12.68	2.22	0.94	TRUE
LEU	43	12.74	2.34	0.94	TRUE
ARG	44	3.95	3.42	0.91	TRUE
ARG	45	6.99	3.74	0.9	TRUE
ILE	46	6.8	2.28	0.94	TRUE
GLY	47	-5.49	2.96	0.92	TRUE

Supplementary Table 6. HX experiment summary tables for T4 lysozyme.

All experiments were performed with KEEP setting in PIGEON. Abbreviations: FP, immobilized protease type XIII. Nep, nepenthesin 2. AP, alanyl aminopeptidase.

Dataset	**AP_pH8_Digest1**	**AP_pH8_Digest2**	**FP_pH8_Digest**
Protein states	APO	APO	APO
Date	11/02/2024	03/18/25	03/14/25
Protease column	AP/pepsin	AP/pepsin	FP/pepsin
Reaction details	50 mM tris hydrochloride, 150 mM NaCl, 0.5 mM TCEP, pH 8.0, temperature 15 °C, final D_2O concentration 90.9%		
Time course (s)	4.3e1-2.5e4	4.4e1-4.5e4	4.2e1-1.6e5
# of time points	5	4	6
Control samples	Maximally-labeled sample (full-D)		
Back-exchange (mean / IQR)	0.23/0.08	0.25/0.08	0.23/0.08
# of peptides	168	182	215
Sequence coverage	0.92	0.93	0.93
Average peptide length / Redundancy	1.1	1.0	0.8

	N1__pH8_Digest	NP_pH8_Digest	AP_pH6_Digest
Protein states	APO	APO	APO
Date	03/24/25	11/04/24	05/10/25
Protease column	Nepenthesin-1	NP/pepsin	AP/pepsin
Reaction details	50 mM tris hydrochloride, 150 mM NaCl, 0.5 mM TCEP, pH 8.0, temperature 15 °C, final D_2O concentration 90.9%		
Time course (s)	4.3e1-2.5e4	4.4e1-2.8e4	4.3e1-4.0e4
# of time points	5	5	5
Control samples	Maximally-labeled sample (full-D)		
Back-exchange (mean / IQR)	0.24/0.11	0.24/0.10	0.25/0.07
# of peptides	179	244	158
Sequence coverage	0.93	0.94	0.92
Average peptide length / Redundancy	1.0	0.7	1.1

	**FP_pH6_Digest**	**N1_pH6_Digest**	**NP_pH6_Digest**
Protein states	APO	APO	APO
Date	05/09/25	05/11/25	05/10/25
Protease column	FP/pepsin	Nepenthesin-1	NP/pepsin
Reaction details	50 mM tris hydrochloride, 150 mM NaCl, 0.5 mM TCEP, pH 8.0, temperature 15 °C, final D_2O concentration 90.9%		
Time course (s)	4.3e1-6.0e4	4.3e1-2.2e4	4.4e1-3.1e4
# of time points	5	4	4
Control samples	Maximally-labeled sample (full-D)		
Back-exchange (mean / IQR)	0.23/0.08	0.24/0.11	0.23/0.11
# of peptides	199	130	190
Sequence coverage	0.93	0.93	0.94
Average peptide length / Redundancy	0.9	1.4	0.9

Supplementary Table 7. ΔG_{op} values for T4 lysozyme.

Res. name	Res. number	Apo	pStd Dev	Confidence	Single resolve
MET	-14				
THR	-13				
GLY	-12	23.89	8.21	0.73	FALSE
HIS	-11	28.04	8.21	0.79	FALSE
HIS	-10	31.43	8.21	0.79	FALSE
HIS	-9	31.43	8.21	0.77	FALSE
HIS	-8	31.43	8.21	0.78	FALSE
HIS	-7	31.43	8.21	0.84	FALSE
HIS	-6	31.43	8.21	0.86	FALSE
GLU	-5	32.34	15.67	0.63	FALSE
ASN	-4	30.62	15.67	0.66	FALSE
LEU	-3	47.22	12.4	0.7	TRUE
TYR	-2	3.82	4.78	0.87	TRUE
PHE	-1	5.19	4.73	0.87	TRUE
GLN	0	2.79	3.28	0.91	TRUE
SER	1	3.63	3.82	0.9	TRUE
ASN	2	5.23	4.02	0.89	TRUE
ILE	3	10.48	1.6	0.96	TRUE
PHE	4	22.15	2.71	0.93	TRUE
GLU	5	29.77	2.66	0.93	TRUE
MET	6	28.83	3.88	0.9	TRUE
LEU	7	35.92	2.07	0.94	TRUE
ARG	8	37.83	2.21	0.94	TRUE
ILE	9	31.98	2.21	0.94	TRUE
ASP	10	34.24	2.22	0.94	TRUE
GLU	11	18.31	5.32	0.86	TRUE
GLY	12	23.78	2.25	0.94	TRUE
LEU	13	6.87	3.99	0.89	TRUE
ARG	14	22.43	1.98	0.95	TRUE

LEU	15	0.52	3.05	0.92	TRUE
LYS	16	31.58	1.97	0.95	TRUE
ILE	17	25.53	3.46	0.91	TRUE
TYR	18	24.11	4	0.89	TRUE
LYS	19	14.46	3.72	0.9	TRUE
ASP	20	7.99	4.09	0.89	TRUE
THR	21	10.24	2.68	0.93	TRUE
GLU	22	9.25	5.18	0.86	TRUE
GLY	23	7.1	4.89	0.87	TRUE
TYR	24	18.76	2.71	0.93	TRUE
TYR	25	26.7	3.8	0.9	TRUE
THR	26	25.01	9.29	0.77	TRUE
ILE	27	31.37	2.28	0.94	TRUE
GLY	28	31.04	3.31	0.91	TRUE
ILE	29	22.14	11.87	0.71	TRUE
GLY	30	15.76	14.61	0.66	TRUE
HIS	31	18.24	3.25	0.91	TRUE
LEU	32	15.96	4.18	0.89	TRUE
LEU	33	27.66	2.35	0.94	TRUE
THR	34	8.99	5.31	0.86	TRUE
LYS	35	3.59	4.7	0.88	TRUE
SER	36	4.19	4.38	0.88	TRUE
PRO	37				
SER	38	3.73	4.42	0.88	TRUE
LEU	39	8.58	3.85	0.9	TRUE
ASN	40	9.15	5.42	0.86	TRUE
ALA	41	2.5	4.16	0.89	TRUE
ALA	42	25.6	3.87	0.9	TRUE
LYS	43	33.52	3.25	0.91	TRUE
SER	44	27.56	2.16	0.94	TRUE
GLU	45	26.23	2.72	0.93	TRUE

LEU	46	23.22	4.28	0.89	TRUE
ASP	47	29.07	3.96	0.89	TRUE
LYS	48	18.18	7.57	0.81	TRUE
ALA	49	32.48	4.39	0.88	TRUE
ILE	50	29.92	5.33	0.86	TRUE
GLY	51	28.59	5.45	0.86	TRUE
ARG	52	13.94	6.69	0.83	FALSE
ASN	53	16.53	6.69	0.83	FALSE
THR	54	16.35	11.85	0.71	FALSE
ASN	55	18.82	11.85	0.72	FALSE
GLY	56	23.68	9.26	0.77	TRUE
VAL	57	29.92	16.4	0.63	TRUE
ILE	58	7.97	3.2	0.93	FALSE
THR	59	11.18	3.2	0.91	FALSE
LYS	60	13.77	3.2	0.9	FALSE
ASP	61	24.31	9.4	0.77	TRUE
GLU	62	12.97	11.87	0.71	TRUE
ALA	63	30.15	3.14	0.91	TRUE
GLU	64	32.24	3.58	0.9	TRUE
LYS	65	24.21	4.72	0.87	TRUE
LEU	66	33.27	5.42	0.86	TRUE
PHE	67	32.41	4.38	0.88	TRUE
ASN	68	25.13	2.86	0.92	TRUE
GLN	69	24.72	3.89	0.9	TRUE
ASP	70	35.16	8.21	0.79	TRUE
VAL	71	36.15	4.23	0.89	TRUE
ASP	72	37.54	2.29	0.94	TRUE
ALA	73	35.31	2.28	0.94	TRUE
ALA	74	33.17	8.24	0.79	TRUE
VAL	75	42.17	9.82	0.76	TRUE
ARG	76	37.02	6.42	0.83	FALSE

GLY	77	38.43	6.42	0.83	FALSE
ILE	78	38.17	7.48	0.81	TRUE
LEU	79	36.38	6.54	0.83	TRUE
ARG	80	30.85	2.39	0.93	TRUE
ASN	81	11.66	10.55	0.74	TRUE
ALA	82	11.04	8.5	0.79	TRUE
LYS	83	19.49	8.9	0.78	TRUE
LEU	84	33.13	2.01	0.94	TRUE
LYS	85	39.14	6.33	0.84	TRUE
PRO	86				
VAL	87	48.96	10.19	0.59	FALSE
TYR	88	51.95	10.19	0.95	FALSE
ASP	89	34.51	3.66	0.9	TRUE
SER	90	22.07	3.18	0.91	TRUE
LEU	91	19.61	2.47	0.93	TRUE
ASP	92	13.59	2.09	0.94	TRUE
ALA	93	47.11	13.57	0.68	TRUE
VAL	94	46.24	6.62	0.83	TRUE
ARG	95	26.77	11.28	0.73	TRUE
ARG	96	41.97	8.7	0.78	TRUE
ALA	97	43.67	12.79	0.7	TRUE
ALA	98	51.4	6.45	0.83	TRUE
LEU	99	49.35	6.69	0.83	TRUE
ILE	100	49.48	6.71	0.83	TRUE
ASN	101	23.23	11.05	0.73	TRUE
MET	102	55.8	6.29	0.84	TRUE
VAL	103	54.16	6.66	0.83	TRUE
PHE	104	51.99	6.71	0.83	TRUE
GLN	105	31.96	3.42	0.91	TRUE
MET	106	11.29	8.04	0.8	TRUE
GLY	107	5.04	5.72	0.85	TRUE

GLU	108	12.42	5.73	0.83	FALSE
THR	109	10.94	5.73	0.81	FALSE
GLY	110	12.95	5.73	0.86	FALSE
VAL	111	9	5.73	0.88	FALSE
ALA	112	11.2	5.73	0.87	FALSE
GLY	113	15.72	12.98	0.69	TRUE
PHE	114	14.36	9.64	0.76	TRUE
THR	115	8.96	5.86	0.85	TRUE
ASN	116	6.09	5.22	0.86	TRUE
SER	117	7.47	5.35	0.86	TRUE
LEU	118	15.84	7.46	0.81	TRUE
ARG	119	13.54	2.69	0.93	TRUE
MET	120	23.16	2.92	0.92	TRUE
LEU	121	33.44	2	0.95	TRUE
GLN	122	29.19	2.92	0.92	TRUE
GLN	123	27.24	2.87	0.92	TRUE
LYS	124	28.34	4.9	0.87	TRUE
ARG	125	26.35	4.99	0.87	TRUE
TRP	126	20.47	5.87	0.85	TRUE
ASP	127	5.89	2.96	0.92	TRUE
GLU	128	6.2	3.43	0.91	TRUE
ALA	129	33.08	2.48	0.93	TRUE
ALA	130	25.31	2.65	0.93	TRUE
VAL	131	27.64	2.34	0.94	TRUE
ASN	132	13.73	1.94	0.95	TRUE
LEU	133	20.44	3.98	0.89	TRUE
ALA	134	18.4	7.14	0.82	TRUE
LYS	135	12.05	5.13	0.86	TRUE
SER	136	8.68	6.74	0.83	TRUE
ARG	137	6.52	6.04	0.84	TRUE
TRP	138	11.94	6.19	0.84	TRUE

TYR	139	22.55	6.86	0.82	TRUE
ASN	140	19.82	6.04	0.85	FALSE
GLN	141	18.91	6.04	0.84	FALSE
THR	142	20.32	7.9	0.8	TRUE
PRO	143				
ASN	144	7.63	5.19	0.84	FALSE
ARG	145	8.47	5.19	0.89	FALSE
ALA	146	27.77	6.55	0.83	TRUE
LYS	147	18.9	13.26	0.69	TRUE
ARG	148	24.33	5.82	0.85	TRUE
VAL	149	30.1	2.66	0.93	TRUE
ILE	150	35.98	4.7	0.84	FALSE
THR	151	39.19	4.7	0.91	FALSE
THR	152	44.93	8.87	0.78	TRUE
PHE	153	48.52	10.16	0.75	TRUE
ARG	154	47.8	7.64	0.81	TRUE
THR	155	29.18	3.61	0.9	TRUE
GLY	156	38.16	2.57	0.93	TRUE
THR	157	29.86	3.3	0.91	TRUE
TRP	158	3.74	3.08	0.92	TRUE
ASP	159	13.79	2.06	0.94	TRUE
ALA	160	15.73	3.28	0.91	TRUE
TYR	161	16.74	4.05	0.93	FALSE
LYS	162	18.31	4.05	0.85	FALSE
ASN	163	11.56	4.05	0.9	FALSE

Supplementary Table 8. HX experiment summary tables for SARS-CoV-2 spike protein.

Data were obtained from Costello *et al.*[7] The peptide list was generated using PIGEON.

Dataset	**Spike_APO_rep1**	**Spike_APO_rep2**	**Spike_APO_rep3**	**Spike_APO_rep4**
Protein states	APO	APO	APO	APO
Date	07/09/20	07/13/20	07/14/20	03/15/21
Time course (s)	15-14400			
# of time points	7			
Control samples	Maximally-labeled sample (full-D)			
Back-exchange (mean / IQR)	0.39/0.14	0.38/0.13	0.38/0.13	0.39/0.14
# of peptides	887	701	701	801
Sequence coverage	0.95	0.92	0.93	0.95
Average peptide length / Redundancy	1.4	1.8	1.8	1.6

Dataset	**Spike_ACE2_rep1**	**Spike_ACE2_rep2**	**Spike_ACE2_rep3**
Protein states	ACE2	ACE2	ACE2
Date	07/08/20	08/06/20	08/06/20
Time course (s)	15-14400		
# of time points	7		
Control samples	Maximally-labeled sample (full-D)		
Back-exchange (mean / IQR)	0.40/0.14	0.40/0.14	0.40/0.14
# of peptides	572	684	688
Sequence coverage	0.95	0.98	0.98
Average peptide length / Redundancy	2.2	1.9	1.9

Supplementary Table 9. ΔG_{op} values for the RBD region of the SARS-CoV-2 spike protein

Res. name	Res. number	Apo	pStd Dev	Confidence	Single resolved	ACE2	pStd Dev	Confidence	Single resolved
ALA	250	39.9	12.09	0.87	FALSE	36.59	16.82	0.62	TRUE
LEU	251	34.88	12.09	0.78	FALSE	25.76	7.24	0.69	FALSE
GLU	252	36.38	12.09	0.53	FALSE	27.25	7.24	0.9	FALSE
PRO	253								
LEU	254	23.69	2.4	0.94	TRUE	24.39	7.24	0.87	FALSE
VAL	255	25.63	2.87	0.92	TRUE	37.8	15.36	0.65	TRUE
ASP	256	4.75	3.99	0.89	TRUE	33.68	11.37	0.68	FALSE
LEU	257	40.94	16.6	0.63	TRUE	29.57	11.37	0.77	FALSE
PRO	258								
ILE	259	7.87	10.72	0.74	TRUE	2.8	5.77	0.85	TRUE
GLY	260								
ILE	261								
ASN	262								
ILE	263								
THR	264								
ARG	265								
PHE	266								
GLN	267								
THR	268								
LEU	269					34.35	16.08	0.63	FALSE
LEU	270					32.01	16.08	0.65	FALSE
ALA	271	27.56	17.47	0.61	TRUE	47.05	17.47	0.61	TRUE
LEU	272	10.36	10.64	0.62	FALSE	15.84	8.63	0.66	FALSE
HIS	273	14.77	10.64	0.88	FALSE	20.25	8.63	0.64	FALSE
ARG	274	17.55	9.74	0.56	FALSE	22.39	8.63	0.88	FALSE
SER	275	17.67	9.74	0.87	FALSE	22.51	8.63	0.9	FALSE
TYR	276	14.48	9.74	0.91	FALSE	19.32	8.63	0.91	FALSE
LEU	277	5.22	4.92	0.87	TRUE	5.14	5.45	0.86	TRUE
THR	278	5.26	4.99	0.82	FALSE	9.93	6.95	0.71	FALSE

PRO	279								
GLY	280	5.32	4.99	0.92	FALSE	9.99	6.95	0.8	FALSE
ASP	281	17.14	10.69	0.61	FALSE	13.08	6.95	0.89	FALSE
SER	282	16.7	10.69	0.75	FALSE	12.63	6.95	0.91	FALSE
SER	283	19.42	10.69	0.89	FALSE	21.1	13.42	0.69	TRUE
SER	284	11.11	5.29	0.76	FALSE	10.88	3.46	0.89	FALSE
GLY	285	8.83	5.29	0.92	FALSE	8.59	3.46	0.91	FALSE
TRP	286	5.92	5.29	0.92	FALSE	5.68	3.46	0.91	FALSE
THR	287	7.59	4.01	0.93	FALSE	6.02	3.46	0.92	FALSE
ALA	288	9.76	4.01	0.88	FALSE	11.37	5.55	0.68	FALSE
GLY	289	8.45	4.01	0.88	FALSE	10.06	5.55	0.89	FALSE
ALA	290	15.27	6.14	0.84	TRUE	11.2	5.55	0.91	FALSE
ALA	291	8.08	3.72	0.9	FALSE	10.23	5.55	0.92	FALSE
ALA	292	8.08	3.72	0.91	FALSE	10.23	5.55	0.91	FALSE
TYR	293	5.93	3.69	0.9	TRUE	24.61	3.63	0.9	TRUE
TYR	294	4.77	3.29	0.91	TRUE	20.1	4.17	0.89	TRUE
VAL	295	36.19	21.38	0.55	TRUE	31.68	7.64	0.74	FALSE
GLY	296	22.3	3.48	0.91	TRUE	34.42	7.64	0.77	FALSE
TYR	297	52.3	9.79	0.76	TRUE	34.82	7.64	0.93	FALSE
LEU	298	37.12	8.29	0.79	TRUE	41.69	19.4	0.58	TRUE
GLN	299	52.95	9.01	0.78	TRUE	46.18	14.36	0.67	TRUE
PRO	300								
ARG	301	52.29	9.62	0.76	TRUE	51.52	8.3	0.79	FALSE
THR	302	14.2	1.64	0.96	TRUE	12.92	5.05	0.87	TRUE
PHE	303	16.19	8.4	0.79	TRUE	32.26	15.04	0.66	TRUE
LEU	304	49.98	12.2	0.71	TRUE	50.95	10.99	0.74	TRUE
LEU	305	31.29	23.23	0.52	TRUE	41.81	12.18	0.71	TRUE
LYS	306	16.7	10.17	0.59	FALSE	3.88	4.24	0.89	TRUE
TYR	307	17.27	10.17	0.71	FALSE	5.3	4.38	0.85	FALSE
ASN	308	21.2	10.17	0.85	FALSE	9.24	4.38	0.89	FALSE
GLU	309	19.33	10.17	0.89	FALSE	7.36	4.38	0.91	FALSE

ASN	310	32.39	17.17	0.59	FALSE	14.27	4.42	0.89	FALSE
GLY	311	32.09	17.17	0.65	FALSE	13.97	4.42	0.88	FALSE
THR	312	22.13	7.56	0.62	FALSE	24.5	4.76	0.93	FALSE
ILE	313	18.53	7.56	0.69	FALSE	20.91	4.76	0.82	FALSE
THR	314	19.84	7.56	0.85	FALSE	18.97	10.97	0.59	FALSE
ASP	315	23.28	7.56	0.87	FALSE	22.41	10.97	0.91	FALSE
ALA	316	20.54	7.56	0.87	FALSE	31.78	15.75	0.64	TRUE
VAL	317	17.56	7.56	0.88	FALSE	50.83	10.87	0.74	TRUE
ASP	318	21.34	7.56	0.88	FALSE	36.94	15.51	0.75	FALSE
CYS	319	24.08	7.56	0.87	FALSE	39.69	15.51	0.56	FALSE
ALA	320	6.43	4.47	0.88	TRUE	27.28	23.79	0.51	TRUE
LEU	321	45.46	14.24	0.67	TRUE	44.39	12.36	0.71	TRUE
ASP	322	37.43	7.14	0.82	TRUE	28.63	4.51	0.88	TRUE
PRO	323								
LEU	324	14.36	8.03	0.8	TRUE	38.53	12.25	0.71	FALSE
SER	325	27.03	4.02	0.88	FALSE	4.88	3.66	0.9	TRUE
GLU	326	27.21	4.02	0.89	FALSE	25.5	4.97	0.87	TRUE
THR	327	24.89	4.02	0.89	FALSE	20.04	3.88	0.9	TRUE
LYS	328	27.03	4.02	0.91	FALSE	14.4	6.54	0.83	TRUE
CYS	329	46.11	13.81	0.68	TRUE	22.91	3.33	0.91	TRUE
THR	330	42.92	19.8	0.57	TRUE	37.99	9.01	0.85	FALSE
LEU	331	51.13	11.18	0.73	TRUE	33.08	9.01	0.71	FALSE
LYS	332	11.78	7.08	0.82	TRUE	19.45	5.02	0.87	TRUE
SER	333	10.11	5.58	0.86	TRUE	53.54	9.07	0.8	FALSE
PHE	334	22.84	7.2	0.82	TRUE	51.08	9.07	0.75	FALSE
THR	335	11.63	5.27	0.86	TRUE	11.67	10.82	0.74	TRUE
VAL	336	2.23	2.81	0.92	FALSE	2.07	2.71	0.93	FALSE
GLU	337	3.67	2.81	0.93	FALSE	3.51	2.71	0.93	FALSE
LYS	338	4.02	2.81	0.92	FALSE	3.86	2.71	0.92	FALSE
GLY	339	26.88	12.29	0.71	TRUE	52.8	9.15	0.77	TRUE
ILE	340	21.93	2.69	0.93	TRUE	22.99	2.61	0.93	TRUE

TYR	341	20.34	3.29	0.91	TRUE	12.02	7.51	0.81	TRUE
GLN	342	9.04	5.1	0.87	TRUE	6.05	3.88	0.9	TRUE
THR	343	8.68	4.84	0.87	TRUE	6.96	5.08	0.87	TRUE
SER	344	8.86	4.95	0.87	TRUE	15.21	3.34	0.93	FALSE
ASN	345	6.4	3.94	0.9	TRUE	16.47	3.34	0.89	FALSE
PHE	346	15.81	5.29	0.86	TRUE	18.99	5.14	0.87	TRUE
ARG	347	6.95	3.67	0.9	TRUE	18.46	4.26	0.89	TRUE
VAL	348	9.24	2.42	0.94	FALSE	5.31	2.81	0.92	TRUE
GLN	349	11.53	2.42	0.93	FALSE	18.3	2.91	0.92	FALSE
PRO	350								
THR	351	3.18	3.02	0.92	FALSE	5.26	4.02	0.89	TRUE
GLU	352	5.48	3.02	0.92	FALSE	7.44	4.78	0.88	TRUE
SER	353	6.23	3.02	0.91	FALSE	3.16	3.49	0.91	TRUE
ILE	354	30.64	8.47	0.79	TRUE	7.89	4.86	0.88	FALSE
VAL	355	5.23	2.68	0.93	TRUE	5.03	4.86	0.86	FALSE
ARG	356	2.66	3.26	0.91	TRUE	12	7.54	0.81	TRUE
PHE	357	8.97	4.61	0.88	TRUE	10.36	3.92	0.92	FALSE
PRO	358								
ASN	359	13.58	2.96	0.93	FALSE	11.9	3.92	0.88	FALSE
ILE	360	9.81	2.96	0.91	FALSE	3.25	3.49	0.91	TRUE
THR	361					29.67	5.49	0.86	TRUE
ASN	362					30.19	7.83	0.85	FALSE
LEU	363					24.77	7.83	0.76	FALSE
CYS	364					7.91	3.94	0.9	FALSE
PRO	365					0	0		
PHE	366					13.63	6.54	0.83	TRUE
GLY	367					10.33	4.09	0.89	TRUE
GLU	368					10.62	3.25	0.89	FALSE
VAL	369					5.44	3.25	0.94	FALSE
PHE	370					46.14	7.56	0.8	FALSE
ASN	371	36.52	13.44	0.58	FALSE	51.45	7.56	0.82	FALSE

ALA	372	35.21	13.44	0.69	FALSE	27.73	3.79	0.9	TRUE
THR	373	32.98	13.44	0.65	FALSE	24.73	4	0.89	TRUE
ARG	374	34.98	13.44	0.84	FALSE	16.85	8.63	0.79	TRUE
PHE	375	37.41	12.77	0.69	FALSE	16.86	7.16	0.82	TRUE
ALA	376	37.87	12.77	0.71	FALSE	52	10.61	0.74	TRUE
SER	377	49.17	11.27	0.76	FALSE	43.93	9.82	0.8	FALSE
VAL	378	44.77	11.27	0.7	FALSE	39.53	9.82	0.8	FALSE
TYR	379	24.88	26.95	0.47	TRUE	39.47	9.82	0.69	FALSE
ALA	380	53.39	8.63	0.79	TRUE	50.66	12.46	0.71	TRUE
TRP	381	31.51	7.27	0.72	FALSE	41.76	11.5	0.72	FALSE
ASN	382	36.02	7.27	0.92	FALSE	46.27	11.5	0.73	FALSE
ARG	383	51.54	11.66	0.72	TRUE	35.12	19.22	0.58	TRUE
LYS	384	4.89	2.88	0.92	FALSE	8.64	6.08	0.84	TRUE
ARG	385	5	2.88	0.93	FALSE	20.46	15.02	0.6	FALSE
ILE	386	11.42	12.18	0.71	TRUE	16.41	15.02	0.71	FALSE
SER	387	15.69	12.47	0.71	TRUE	35.91	12.4	0.68	FALSE
ASN	388	32.49	11.29	0.7	FALSE	39.62	12.4	0.77	FALSE
CYS	389	33.34	11.29	0.77	FALSE	40.47	12.4	0.68	FALSE
VAL	390	50.68	10.95	0.74	TRUE	31.77	14.84	0.66	TRUE
ALA	391	45.15	9.33	0.77	FALSE	38.47	18.21	0.6	TRUE
ASP	392	46.53	9.33	0.77	FALSE	36.62	22.4	0.54	TRUE
TYR	393	25.51	9.76	0.68	FALSE	8.02	9.88	0.76	TRUE
SER	394	30.46	9.76	0.8	FALSE	16.26	13.53	0.51	FALSE
VAL	395	25.78	9.76	0.81	FALSE	11.58	13.53	0.93	FALSE
LEU	396	37.44	19.18	0.59	TRUE	13.95	12.5	0.7	TRUE
TYR	397	5.74	3.43	0.91	TRUE	53.46	9.95	0.76	TRUE
ASN	398	7.93	3.76	0.88	FALSE	34.82	6.63	0.73	FALSE
SER	399	8.79	3.76	0.91	FALSE	35.67	6.63	0.86	FALSE
ALA	400	6.56	3.76	0.91	FALSE	33.45	6.63	0.86	FALSE
SER	401	4.96	3.22	0.91	FALSE	33.85	6.63	0.88	FALSE
PHE	402	3.19	3.22	0.92	FALSE	7.12	4.92	0.87	TRUE

SER	403	32.56	7.15	0.82	TRUE	13.98	11.23	0.73	TRUE
THR	404	30.49	4.04	0.89	TRUE	20.18	16.25	0.64	TRUE
PHE	405	54.03	9.12	0.77	TRUE	51.4	10.85	0.74	TRUE
LYS	406	50.99	12.49	0.71	TRUE	17.14	6.41	0.84	TRUE
CYS	407	10.37	3.7	0.9	FALSE	54.79	9.61	0.76	TRUE
TYR	408	7.75	3.7	0.9	FALSE	50.73	12.27	0.71	TRUE
GLY	409	5.46	3.93	0.9	TRUE	59.74	6.99	0.81	FALSE
VAL	410	44.99	15.81	0.64	TRUE	56.6	6.99	0.82	FALSE
SER	411	8.49	3.46	0.91	FALSE	60.94	6.99	0.84	FALSE
PRO	412								
THR	413	5.4	3.46	0.91	FALSE	23.5	3.92	0.9	TRUE
LYS	414	8.09	3.46	0.9	FALSE	49.66	12.05	0.71	TRUE
LEU	415	15.99	4.23	0.89	TRUE	52.52	10.22	0.75	TRUE
ASN	416	22.98	7.15	0.82	TRUE	28.97	8.4	0.7	FALSE
ASP	417	9.12	5.15	0.87	TRUE	29.78	8.4	0.87	FALSE
LEU	418	16.67	5.53	0.86	TRUE	23.05	8.4	0.83	FALSE
CYS	419	24.89	3.92	0.9	FALSE	29.71	8.4	0.78	FALSE
PHE	420	24.32	3.92	0.89	FALSE	20.26	9.97	0.76	TRUE
THR	421	39.16	8.11	0.78	FALSE	16.15	6	0.85	TRUE
ASN	422	43.16	8.11	0.81	FALSE	35.65	8.5	0.69	FALSE
VAL	423	37.05	8.11	0.75	FALSE	29.54	8.5	0.78	FALSE
TYR	424	36.88	8.11	0.85	FALSE	29.37	8.5	0.91	FALSE
ALA	425	49.15	15.47	0.65	TRUE	45.29	15.92	0.64	TRUE
ASP	426	38.91	6.82	0.77	FALSE	51.73	9.23	0.77	TRUE
SER	427	39.43	6.82	0.89	FALSE	29.86	4.5	0.88	TRUE
PHE	428	43.16	12.28	0.71	TRUE	15.1	5.99	0.85	TRUE
VAL	429	48.27	12.31	0.71	TRUE	21.1	7.63	0.81	TRUE
ILE	430	49.28	6.52	0.84	FALSE	48.28	11.88	0.72	TRUE
ARG	431	53.39	6.52	0.83	FALSE	50.37	11.09	0.73	TRUE
GLY	432	29.45	11.49	0.73	TRUE	25.67	9.75	0.88	FALSE
ASP	433	14.21	4.5	0.88	TRUE	26.14	9.75	0.66	FALSE

GLU	434	28.13	3.21	0.91	TRUE	9.57	6.4	0.84	TRUE
VAL	435	43.87	13.77	0.68	TRUE	44.26	13.88	0.68	TRUE
ARG	436	2.31	3.42	0.92	FALSE	41.42	8.78	0.72	FALSE
GLN	437	4.25	3.42	0.9	FALSE	43.36	8.78	0.85	FALSE
ILE	438	1.29	2.57	0.93	TRUE	45.07	13.34	0.69	TRUE
ALA	439	51.92	10.53	0.75	TRUE	16.72	5.07	0.87	TRUE
PRO	440								
GLY	441	17.07	4.9	0.83	FALSE	10.2	4.64	0.83	FALSE
GLN	442	19.93	4.9	0.9	FALSE	13.05	4.64	0.9	FALSE
THR	443	19.36	4.9	0.9	FALSE	12.48	4.64	0.91	FALSE
GLY	444	19.59	4.9	0.92	FALSE	7	6.01	0.8	FALSE
LYS	445	19.36	4.9	0.82	FALSE	6.77	6.01	0.9	FALSE
ILE	446	30.62	18.85	0.59	TRUE	20.27	4.21	0.89	TRUE
ALA	447	26.95	21.89	0.54	TRUE	51.99	7.26	0.81	FALSE
ASP	448	50.56	11.43	0.73	TRUE	53.88	7.26	0.82	FALSE
TYR	449	51.96	11.27	0.73	TRUE	51.65	10.97	0.74	TRUE
ASN	450	51.31	11.63	0.72	TRUE	52.07	10.82	0.74	TRUE
TYR	451	51.34	10.79	0.74	TRUE	23.01	5.2	0.86	TRUE
LYS	452	44.11	7.56	0.83	FALSE	13.33	12.36	0.71	TRUE
LEU	453	41.42	7.56	0.86	FALSE	49.1	13.63	0.68	TRUE
PRO	454								
ASP	455	43.26	7.56	0.74	FALSE	26.68	10.52	0.66	FALSE
ASP	456	5.56	3.83	0.9	TRUE	27.04	10.52	0.84	FALSE
PHE	457	11.53	1.78	0.95	TRUE	31.54	21.01	0.56	TRUE
THR	458	31.05	7.33	0.72	FALSE	33.35	12.2	0.68	FALSE
GLY	459	32.07	7.33	0.92	FALSE	34.38	12.2	0.74	FALSE
CYS	460	10.96	5.11	0.87	TRUE	34.15	15.02	0.61	FALSE
VAL	461	25.36	10.62	0.89	FALSE	28.78	15.02	0.71	FALSE
ILE	462	21.25	10.62	0.62	FALSE	26.02	8.01	0.71	FALSE
ALA	463	7.99	3.84	0.88	FALSE	29.67	8.01	0.85	FALSE
TRP	464	6.96	3.84	0.92	FALSE	28.65	8.01	0.84	FALSE

ASN	465	11.47	3.84	0.89	FALSE	31.59	6.92	0.85	FALSE
SER	466	13.24	3.84	0.91	FALSE	33.35	6.92	0.8	FALSE
ASN	467	33.42	9.79	0.79	FALSE	12.1	4.76	0.88	TRUE
ASN	468	33.54	9.79	0.78	FALSE	37.13	20.86	0.56	TRUE
LEU	469	27.43	9.79	0.71	FALSE	16.52	9.23	0.77	TRUE
ASP	470	16.4	3.97	0.89	TRUE	28.65	5.2	0.93	FALSE
SER	471	14.22	5.35	0.83	FALSE	30.37	5.2	0.75	FALSE
LYS	472	14.61	5.35	0.89	FALSE	30.75	5.2	0.93	FALSE
VAL	473	1.33	2.89	0.92	FALSE	25.96	5.2	0.86	FALSE
GLY	474	3.67	2.89	0.92	FALSE	40.3	26.04	0.48	TRUE
GLY	475	19.12	9.71	0.6	FALSE	25.86	4.27	0.89	TRUE
ASN	476	22.09	9.71	0.81	FALSE	19.37	11.12	0.73	TRUE
TYR	477	18.6	9.71	0.91	FALSE	4.87	3.12	0.92	FALSE
ASN	478	19.09	11.08	0.73	TRUE	7.67	3.12	0.92	FALSE
TYR	479	6.58	5.08	0.87	TRUE	4.87	3.12	0.91	FALSE
LEU	480	22.82	2.88	0.92	TRUE				
TYR	481	37.27	22.49	0.53	TRUE	37.14	17.15	0.62	TRUE
ARG	482	40.72	13.37	0.75	FALSE	18.18	5.52	0.82	FALSE
LEU	483	37.92	13.37	0.63	FALSE	15.38	5.52	0.88	FALSE
PHE	484	19.46	5.08	0.87	TRUE	14.87	5.52	0.87	FALSE
ARG	485	27.55	3.11	0.92	TRUE	13.79	7.2	0.82	TRUE
LYS	486	4.99	3.35	0.91	FALSE	22.39	10.65	0.91	FALSE
SER	487	6.76	3.35	0.92	FALSE	24.16	10.65	0.73	FALSE
ASN	488	8.47	3.35	0.9	FALSE	25.87	10.65	0.62	FALSE
LEU	489	5.11	3.67	0.9	TRUE	34.32	12.02	0.66	FALSE
LYS	490	29.34	2.61	0.93	TRUE	34.38	12.02	0.78	FALSE
PRO	491								
PHE	492	5.8	5.07	0.87	TRUE	20.49	4.09	0.9	FALSE
GLU	493	40.54	9.06	0.78	TRUE	22.96	4.09	0.9	FALSE
ARG	494	17.73	6.92	0.82	TRUE	22.85	4.09	0.88	FALSE
ASP	495	27.13	8.74	0.78	TRUE	28.08	4.31	0.89	TRUE

ILE	496	10.1	9.8	0.76	TRUE	14.86	4.17	0.89	TRUE
SER	497	17.18	5.56	0.86	TRUE	21.73	4.92	0.91	FALSE
THR	498	29.08	2.73	0.93	TRUE	22.25	4.92	0.84	FALSE
GLU	499	6.04	3.87	0.9	TRUE	21.76	11.63	0.72	TRUE
ILE	500	10.72	2.67	0.93	TRUE	43.31	18.92	0.59	TRUE
TYR	501	10.2	2.1	0.94	TRUE	5.19	5.59	0.65	FALSE
GLN	502	8.6	3.99	0.89	TRUE	8.67	5.59	0.89	FALSE
ALA	503	5.92	4.17	0.89	TRUE	9.18	5.59	0.9	FALSE
GLY	504	5.89	4.28	0.89	FALSE	7.87	5.59	0.91	FALSE
SER	505	9.14	4.28	0.87	FALSE	11.12	5.59	0.91	FALSE
THR	506	7.37	4.28	0.9	FALSE	9.35	5.59	0.91	FALSE
PRO	507								
CYS	508	20.78	18.09	0.6	FALSE	8.4	4.05	0.89	FALSE
ASN	509	14.78	6.22	0.63	FALSE	12.17	4.05	0.89	FALSE
GLY	510	10.49	6.22	0.9	FALSE	7.89	4.05	0.9	FALSE
VAL	511	5.81	6.22	0.89	FALSE	19.11	8.56	0.77	FALSE
GLU	512	7.42	6.22	0.9	FALSE	20.72	8.56	0.59	FALSE
GLY	513	7.83	6.22	0.92	FALSE	21.13	8.56	0.91	FALSE
PHE	514	5.88	4.65	0.88	TRUE	21.73	8.56	0.92	FALSE
ASN	515	52.28	11.19	0.73	TRUE	6.72	4.35	0.89	TRUE
CYS	516	32.15	12.55	0.7	TRUE	25.2	13.19	0.64	FALSE
TYR	517	32.46	10.01	0.71	FALSE	21.43	13.19	0.75	FALSE
PHE	518	29.78	10.01	0.81	FALSE	22.56	8.54	0.79	FALSE
PRO	519								
LEU	520	6.73	3.81	0.89	FALSE	37.53	6.29	0.76	FALSE
GLN	521	10.55	3.81	0.91	FALSE	41.35	6.29	0.86	FALSE
SER	522	43.83	12.29	0.74	FALSE	45.47	6.29	0.9	FALSE
TYR	523	40.74	12.29	0.68	FALSE	12.11	9.06	0.78	TRUE
GLY	524	18.2	13.43	0.69	TRUE	16	5.27	0.86	TRUE
PHE	525	4.34	3.37	0.91	FALSE	34.8	7.7	0.69	FALSE
GLN	526	5.42	3.37	0.91	FALSE	35.88	7.7	0.94	FALSE

PRO	527								
THR	528	3.02	3.17	0.92	TRUE	17.34	2.3	0.94	FALSE
ASN	529	22.64	7.69	0.66	FALSE	36.56	10.68	0.7	FALSE
GLY	530	20.36	7.69	0.87	FALSE	34.28	10.68	0.69	FALSE
VAL	531	15.68	7.69	0.92	FALSE	29.59	10.68	0.85	FALSE
GLY	532	3.46	2.78	0.93	TRUE	26.11	10.44	0.63	FALSE
TYR	533	14.26	3.43	0.89	FALSE	26.51	10.44	0.82	FALSE
GLN	534	15.46	3.43	0.93	FALSE	27.71	10.44	0.81	FALSE
PRO	535								
TYR	536	55.41	15.96	0.55	FALSE	21.39	13.35	0.54	FALSE
ARG	537	59.07	15.96	0.75	FALSE	25.04	13.35	0.89	FALSE
VAL	538	24.63	24.23	0.51	TRUE	6.59	6.25	0.84	TRUE
VAL	539	49.72	12.37	0.71	TRUE	35.94	8.47	0.79	TRUE
VAL	540	49.48	12.74	0.7	TRUE	47.64	12.77	0.7	TRUE
LEU	541	48.28	12.79	0.7	TRUE	46.2	12.98	0.7	TRUE
SER	542	31.52	3.16	0.92	TRUE	30.94	1.73	0.95	TRUE
PHE	543	52.43	9.95	0.76	TRUE	50.38	11.75	0.72	TRUE
GLU	544					11.27	4.01	0.91	FALSE
LEU	545	7.94	3.24	0.91	TRUE	7.4	4.01	0.88	FALSE
LEU	546	19.66	5.24	0.78	FALSE	4.12	4.86	0.87	TRUE
HIS	547	25.27	5.24	0.91	FALSE	16.68	5.06	0.87	TRUE
ALA	548	26.95	5.24	0.9	FALSE	30.4	38.33	0.34	FALSE
PRO	549								
ALA	550	5.09	4.82	0.87	TRUE				

3. Supplementary notes

Supplementary Note 1: The pseudocode of PFNet.

Algorithm 1: PFNet

Input: Peptide-timepoint observations $\{p, t\}$: observed isotopic mass envelopes $\mathbf{p}_{p,t} \in \mathbb{R}^B$, t_0 isotopic mass envelopes $\mathbf{p}_{p,0} \in \mathbb{R}^B$, peptide start and end positions $\mathbf{s}$ and $\mathbf{e}$, labeling time t, and back-exchange $\mathbf{b}_{\text{ex}}$;

Residue-level features for $i = 1, \ldots, L$: residue position i, intrinsic exchange rate $k_{\text{ch},i}$, resolution grouping features $\mathbf{g}_{\text{res}}$, saturation $\mathbf{s}_{\text{sat}}$, and proline mask $\mathbf{m}_{\text{pro}}$;

Number of recycling cycles C.

Output: Predicted residue-level $\log_{10}$ exchange rates $\tilde{y}_i$ and predicted confidence scores $\tilde{u}_i$.

Encode all peptide-timepoint tokens in parallel:
$\mathbf{H}^{\text{env}} \leftarrow \text{ProbEnc}(\mathbf{P}, \mathbf{b}_{\text{ex}})$

$\mathbf{H}^{\text{pos}} \leftarrow \text{concat}\big(\text{PosEnc}(\mathbf{s}), \text{PosEnc}(\mathbf{e})\big)$

$\mathbf{H}^{\text{time}} \leftarrow \text{TimeEnc}(\mathbf{t})$

$\mathbf{H}^{\text{pep}} \leftarrow \text{concat}(\mathbf{H}^{\text{pos}}, \mathbf{H}^{\text{time}}, \mathbf{H}^{\text{env}})$

$\mathbf{m}_{\text{pep}} \leftarrow \text{PeptidePaddingMask}(\mathbf{s})$

$\mathbf{M} \leftarrow \text{TransformerEncoder}(\mathbf{H}^{\text{pep}}, \mathbf{m}_{\text{pep}})$

Encode all residue tokens in parallel:
$\mathbf{R} \leftarrow \text{concat}\big(\text{PosEnc}(\mathbf{i}), \text{Enc}(\log k_{\text{ch}}), \text{Enc}(\mathbf{g}_{\text{res}}), \text{Enc}(\mathbf{s}_{\text{sat}})\big)$

$\mathbf{m}_{\text{res}} \leftarrow \text{ResidueMask}(\log k_{\text{ex}}, \mathbf{g}_{\text{res}}, \mathbf{m}_{\text{pro}})$

Initialize $\tilde{\mathbf{y}}^{(0)} \leftarrow \mathbf{0}$, $\tilde{\mathbf{u}}^{(0)} \leftarrow \mathbf{0}$, $\boldsymbol{\epsilon}^{(0)} \leftarrow \mathbf{0}$

for $c = 1, \ldots, C$ **do**

- $\mathbf{H}^{\text{rec},(c)} \leftarrow \text{RecycleEnc}(\tilde{\mathbf{y}}^{(c-1)}, \tilde{\mathbf{u}}^{(c-1)})$
- $\mathbf{Q}^{(c)} \leftarrow \text{concat}(\mathbf{R}, \mathbf{H}^{\text{rec},(c)})$
- $\mathbf{M}^{(c)} \leftarrow \mathbf{M} + \text{Enc}(\boldsymbol{\epsilon}^{(c-1)})$
- $\mathbf{H}^{(c)} \leftarrow \text{TransformerDecoder}(\mathbf{Q}^{(c)}, \mathbf{M}^{(c)}, \mathbf{m}_{\text{res}}, \mathbf{m}_{\text{pep}})$
- $\tilde{\mathbf{y}}^{(c)} \leftarrow \text{Linear}(\mathbf{H}^{(c)})$
- $\tilde{\mathbf{u}}^{(c)} \leftarrow \text{MLP}_{\text{conf}}(\mathbf{H}^{(c)})$
- $\hat{\mathbf{P}}^{(c)} \leftarrow \text{HDXForward}\left(\mathbf{P}_0, \tilde{\mathbf{y}}^{(c)}, \mathbf{s}, \mathbf{e}, \mathbf{t}, \mathbf{b}_{\text{ex}}\right)$
- $\boldsymbol{\epsilon}^{(c)} \leftarrow \sum_{b=1}^{B} \left| \hat{\mathbf{P}}^{(c)}_{:,b} - \mathbf{P}_{:,b} \right|$

Return $\tilde{\mathbf{y}} \leftarrow \tilde{\mathbf{y}}^{(C)}$, $\tilde{\mathbf{u}} \leftarrow \tilde{\mathbf{u}}^{(C)}$

Algorithm 2: PosEnc

Input: Positions x and embedding dimension d.
Output: Position embeddings H^{pos}.

for $j = 0, 2, \ldots, d-2$ do
  $E_{:,j} \leftarrow \sin(x\, a_j)$
  $E_{:,j+1} \leftarrow \cos(x\, a_j)$
$H^{mlp} \leftarrow \text{MLP}(E)$
$H^{pos} \leftarrow \text{LayerNorm}(H^{mlp})$
Return H^{pos}

Algorithm 3: ProbEnc

Input: Observed isotopic mass envelopes $P \in R^{N \times B}$ and back-exchange b_{ex}.
Output: Peptide-timepoint envelope embeddings H^{env}.

$c_{ex} \leftarrow \text{MLP}_{cond}(b_{ex})$
$Z \leftarrow \text{reshape}(P)$ cp*add channel dimension
for $\ell = 1, 2, 3$ do
  $Z \leftarrow \text{Conv1D}_{\ell}(Z)$
  $Z \leftarrow \text{CondBatchNorm}_{\ell}(Z; c_{ex})$
  $Z \leftarrow \text{ELU}(Z)$
$h_{cnn} \leftarrow \text{MLP}(\text{flatten}(Z))$
$h_{res} \leftarrow \text{Linear}(P)$
$H^{env} \leftarrow \text{LayerNorm}(h_{cnn} + h_{res})$
Return H^{env}

Algorithm 4: TimeEnc

Input: Labeling times t and optional padding mask m_{time}.
Output: Time embeddings H^{time}.

$m_{time} \leftarrow \text{IsPadding}(t)$ if no mask is provided
$\tau \leftarrow \log_2(t + \epsilon)$
$\tau \leftarrow \text{MaskFill}(\tau, m_{time}, -100)$
$H^{disc} \leftarrow \text{Embedding}(\text{NearestGridIndex}(\tau))$
$H^{disc} \leftarrow \text{LayerNorm}(H^{disc})$
$H^{cont} \leftarrow \text{PosEnc}(\tau)$
$H^{time} \leftarrow \text{LayerNorm Linear concat}(H^{disc}, H^{cont})$
$H^{time} \leftarrow \text{MaskFill}(H^{time}, m_{time}, 0)$
Return H^{time}

Supplementary Note 2: Technical details: benchmarking other methods for ΔG_{op} determination

All other benchmarked software input and output files can be found on Zenodo at DOI 10.5281/zenodo.19443809

HDsite[8]

HXMS files for EEHEE_rd4_087, HHH_rd4_0557, and T4 were converted into HDsite compatible file formats. Within the HDsite GUI, ETD was disabled, Hydrogen to Deuterium exchange was selected, and Filter data was unchecked. Additionally, Envelope fitting and the LsqNonLin algorithm were utilized, while the back exchange correction was enabled. Back exchange information was provided via the input file for each respective protein. Temperature in Celsius and pD values were entered for each protein. The minimum deuterium value was set to 0.0093, while the maxD was adjusted according to each protein's specific saturation value. All timepoints, excluding t=0 and t=inf, were entered into the “hx timepoints” section in seconds with their respective files.

The "region to fit" parameters were defined as follows: EEHEE_rd4_087: residues 8–4 and 2–10; HHH_rd4_0557: residues 29–47 and 1–2; T4: residues 1–50, 55–100, 103–115, 115–140, 140–158, and 165–178.

HDfleX[9]

HXMS files for EEHEE_rd4_087, HHH_rd4_0557, and T4 were converted into the DynamX (Waters) Cluster file format. Each duplicate peptide was assigned to a separate replicate condition, followed by an underscore and the “1, 2, 3” naming convention supported by HDfleX. For each dataset, the number of replicates defined in the program was determined by the peptide with the highest replicate count.

The maxD state was assigned a time of “9999999” minutes under a separate condition labeled with the _inf suffix. The specific pH for each protein was entered into the reference and experimental pH fields of the GUI. A minimum time value of “0” was applied to all proteins, while the maximum timepoints were set to correspond to each protein's specific maximum duration. Temperatures were entered in Kelvin for each respective protein.

For the EEHEE_rd4_087 and HHH_rd4_0557 datasets, the “Include intrinsic exchange rates” option was enabled. However, this option was disabled for the T4 dataset to avoid excessive runtimes. Several peptides were removed that caused errors that prevented the assignments of intrinsic rates or deuterium uptake fits. Following the completion of the fittings, each protein sequence was entered into the “Protein Sequence Information” field within the “Absolute Uptake” section of the GUI. Finally, protection factors were calculated and exported for EEHEE_rd4_087 and HHH_rd4_0557, while the observed rate constant was exported for T4.

ExPfact[10]

HXMS files for EEHEE_rd4_087, HHH_rd4_0557, and T4 were converted into multiple exPfact formatted files. The exPfact Python scripts were modified to allow for the use of peptides with different timepoints relative to other peptides in each dataset. This was achieved by using a mask during the cost function calculation and adding “NaN” padding values to missing timepoints. The modified code can be found at Zenodo at DOI 10.5281/zenodo.19443809. For each protein, the modified exPfact scripts were used. First, exPfact.py was run on the command line with the respective pD and temperatures in Kelvin for each protein using the --pH and --temp arguments. The generated input files containing the experimental deuterium uptakes, peptide assignments, and protein sequences for each respective protein were entered using the --dexp, --ass, and --seq arguments. Additionally, 5000 random sets of protection factors and multiple solutions were set using the --rand and --rep arguments. A harm score of 0 was set for all proteins using the --harm argument. Once complete, the top 200 predictions were analyzed using the descriptive_statistics.py script and the output file from the previous step using the --res and --top arguments. The clustering.py script was finally used to obtain the clustered site values. For EEHEE_rd4_087, the 2-47.mod file was used; for HHH_rd4_0557, the 2-47.mod file was used; and for T4, the 3-178.mod file was used for comparing to NMR values.

4. Accessing data and running PFNet

Benchmark and experimental HX-MS datasets

The synthetic benchmark and experimental HX-MS datasets are provided in HXMS format, which includes isotopic mass envelope peaks or centroids for all labeling time points and peptides, defined by their start and end residues, along with key experimental parameters. Both datasets are available at Zenodo (https://zenodo.org/records/17353731). The raw HX-MS data of T4 lysozyme is available from the PRIDE database (PXD069693). The raw HX-MS data of ecDHFR and EEHEE_rd4_087 mini protein were previously reported[6] and are available from the PRIDE database (PXD057539).

The experimental dataset (HXMS format) is also provided as Supplementary Data for convenience.

Installing and running PFNet

PFNet accepts input files in **HXMS format**,[11] which can be generated using **PFLink** (https://huggingface.co/spaces/glasgow-lab/PFLink), a package that supports data exported from major HX-MS analysis software packages, including BioPharma Finder (Fisher), HDExaminer (Trajan), DynamX (Waters), and HDX Workbench.

Code for dataset generation, model training, inference, and access to pretrained models is available at:https://github.com/glasgowlab/PFNet. Installation instructions are provided in the *README.md* file within the same repository.

To facilitate broader accessibility, the trained PFNet model is also available as a cloud-based application on Hugging Face Spaces: https://huggingface.co/spaces/glasgow-lab/PFNet.

5. Supplementary References

1. Dixon, M. E., Hitchens, T. K. & Bryant, R. G. Comparisons of Pressure and Temperature Activation Parameters for Amide Hydrogen Exchange in T4 Lysozyme. *Biochemistry* **39**, 248–254 (2000).
2. Llinás, M., Gillespie, B., Dahlquist, F. W. & Marqusee, S. The energetics of T4 lysozyme reveal a hierarchy of conformations. *Nat. Struct. Biol.* **6**, 1072–1078 (1999).
3. Vendruscolo, M., Paci, E., Dobson, C. M. & Karplus, M. Rare Fluctuations of Native Proteins Sampled by Equilibrium Hydrogen Exchange. *J. Am. Chem. Soc.* **125**, 15686–15687 (2003).
4. Thompson, S., Zhang, Y., Ingle, C., Reynolds, K. A. & Kortemme, T. Altered expression of a quality control protease in E. coli reshapes the in vivo mutational landscape of a model enzyme. *Elife* **9**, e53476 (2020).
5. Weis, D. D. Recommendations for the Propagation of Uncertainty in Hydrogen Exchange-Mass Spectrometric Measurements. *J. Am. Soc. Mass Spectrom.* **32**, 1610–1617 (2021).
6. Lu, C., Wells, M. L., Reckers, A., McBride, S. K. & Glasgow, A. Site-resolved energetic information from HX–MS experiments. *Nat. Chem. Biol.* **22**, 307–317 (2026).
7. Costello, S. M. *et al.* The SARS-CoV-2 spike reversibly samples an open-trimer conformation exposing novel epitopes. *Nat. Struct. Mol. Biol.* **29**, 229–238 (2022).
8. Kan, Z.-Y., Walters, B. T., Mayne, L. & Englander, S. W. Protein hydrogen exchange at residue resolution by proteolytic fragmentation mass spectrometry analysis. *Proc. Natl. Acad. Sci.* **110**, 16438–16443 (2013).
9. Seetaloo, N., Kish, M. & Phillips, J. J. HDfleX : Software for Flexible High Structural Resolution of Hydrogen/Deuterium-Exchange Mass Spectrometry Data. *Anal. Chem.* **94**, 4557–4564 (2022).
10. Stofella, M., Skinner, S. P., Sobott, F., Houwing-Duistermaat, J. & Paci, E. High-Resolution Hydrogen–Deuterium Protection Factors from Sparse Mass Spectrometry Data Validated by Nuclear Magnetic Resonance Measurements. *J. Am. Soc. Mass Spectrom.* **33**, 813–822 (2022).
11. Weber, K. C., Lu, C., Alvarez, R. V., Pascal, B. D. & Glasgow, A. HXMS: a standardized file format for HX-MS data. *Bioinformatics* **42**, btag113 (2026).
