# Supplementary material for: A machine learning method for calculating highly localized protein stabilities
Source: Nat Commun. 2026 Sep 1;17:10414. doi: 10.1038/s41467-026-75590-9 (PMC13631364; doi:10.1038/s41467-026-75590-9)
Supplement: Supplementary file 2 [file 41467_2026_75590_MOESM2_ESM.pdf]

Description of Additional Supplementary Files

File Name: Supplementary Data 1

Description: Experimental HX-MS datasets in HXMS format.

File Name: Supplementary Data 2

Description: Experimental deuterium uptake plots and model fitting plots
