# Supplementary material for: A machine learning method for calculating highly localized protein stabilities
Source: Nat Commun. 2026 Sep 1;17:10414. doi: 10.1038/s41467-026-75590-9 (PMC13631364; doi:10.1038/s41467-026-75590-9)
Supplement: Supplementary file 6 [file 41467_2026_75590_MOESM4_ESM.zip › Supplementary Data 2/PFNet_uptake_ecDHFR_APO_1.pdf]

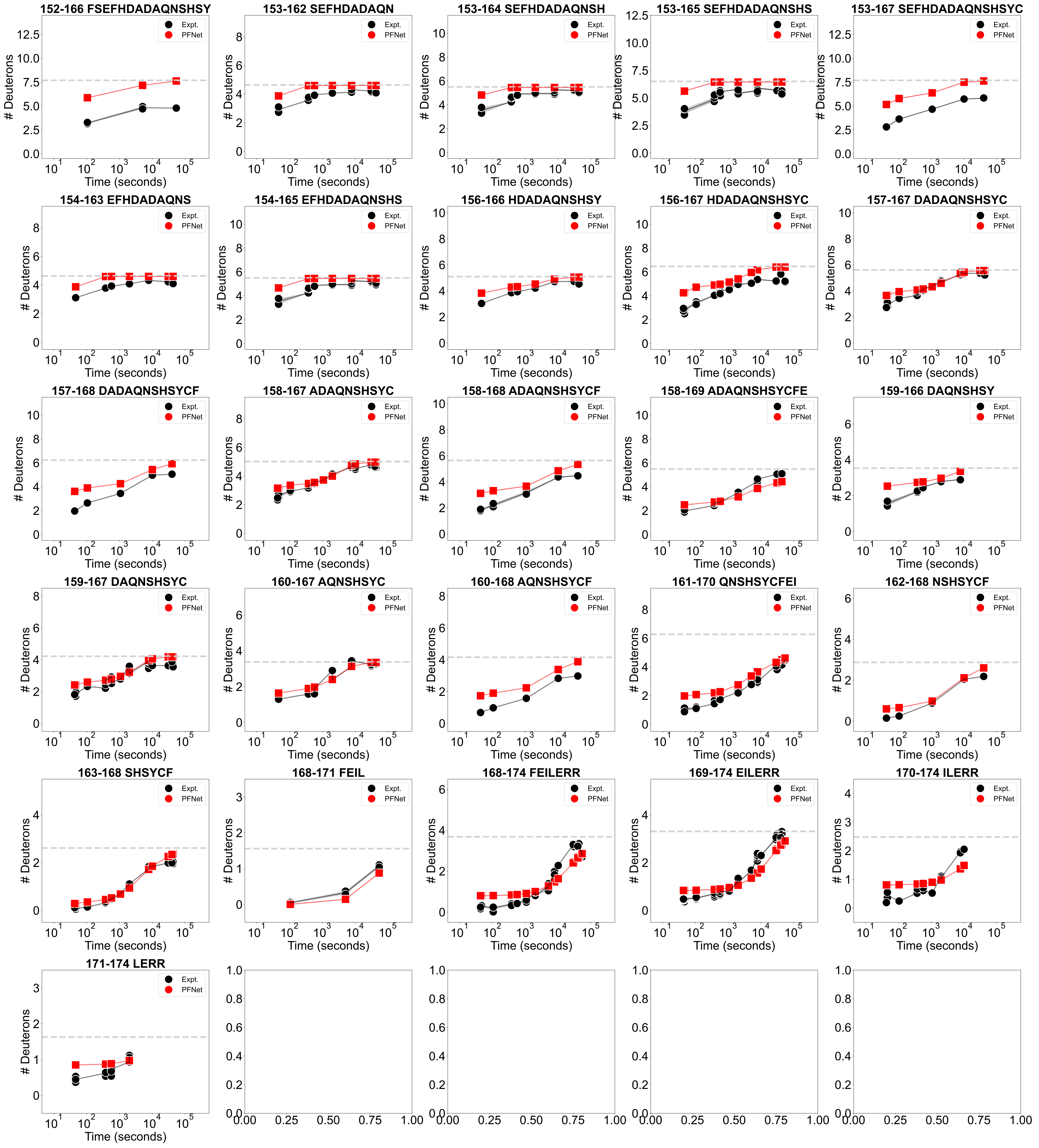

152-166 FSEFHDADAQNSHSY
153-162 SEFHDADAQN
153-164 SEFHDADAQNSH
153-165 SEFHDADAQNSHS
153-167 SEFHDADAQNSHSYC
154-163 EFHDADAQNS
154-165 EFHDADAQNSHS
156-166 HDADAQNSHSY
156-167 HDADAQNSHSYC
157-167 DADAQNSHSYC
157-168 DADAQNSHSYCF
158-167 ADAQNSHSYC
158-168 ADAQNSHSYCF
158-169 ADAQNSHSYCFE
159-166 DAQNSHSY
159-167 DAQNSHSYC
160-167 AQNSHSYC
160-168 AQNSHSYCF
161-170 QNSHSYCFEI
162-168 NSHSYCF
163-168 SHSYCF
168-171 FEIL
168-174 FEILERR
169-174 EILERR
170-174 ILERR
171-174 LERR
Expt.
PFNet
Deuterons
Time (seconds)
