# Supplementary material for: A machine learning method for calculating highly localized protein stabilities
Source: Nat Commun. 2026 Sep 1;17:10414. doi: 10.1038/s41467-026-75590-9 (PMC13631364; doi:10.1038/s41467-026-75590-9)
Supplement: Supplementary file 6 [file 41467_2026_75590_MOESM4_ESM.zip › Supplementary Data 2/PFNet_uptake_HHH_rd4_0557_APO_0.pdf]

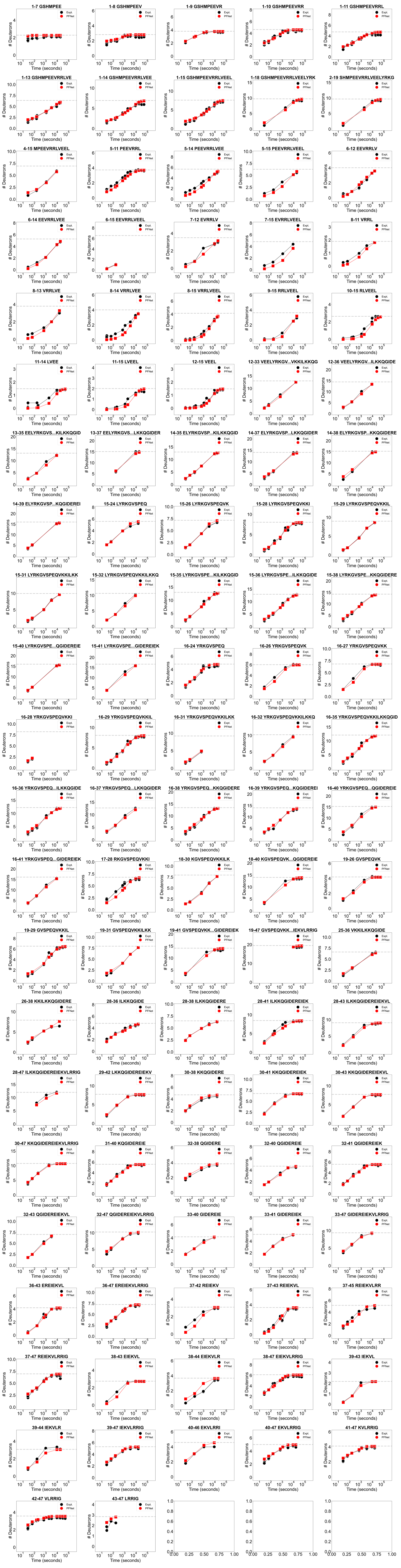

1-7 GSHMPEE
1-8 GSHMPEEV
1-9 GSHMPEEVR
1-10 GSHMPEEVRR
1-11 GSHMPEEVRRL
1-13 GSHMPEEVRRLVE
1-14 GSHMPEEVRRLVEE
1-15 GSHMPEEVRRLVEEL
1-18 GSHMPEEVRRLVEELYRK
2-19 SHMPEEVRRLVEELYRKG
4-15 MPEEVRRLVEEL
5-11 PEEVRRL
5-14 PEEVRRLVEE
5-15 PEEVRRLVEEL
6-12 EEVRRLV
6-14 EEVRRLVEE
6-15 EEVRRLVEEL
7-12 EVRRLV
7-15 EVRRLVEEL
8-11 VRRL
8-13 VRRLVE
8-14 VRRLVEE
8-15 VRRLVEEL
9-15 RRLVEEL
10-15 RLVEEL
11-14 LVEE
11-15 LVEEL
12-15 VEEL
12-33 VEELYRKGV...VKKILKKQG
12-36 VEELYRKGV...ILKKQGIDE
13-35 EELYRKGVS...KILKKQGID
13-37 EELYRKGVS...LKKQGIDER
14-35 ELYRKGVSP...KILKKQGID
14-37 ELYRKGVSP...LKKQGIDER
14-38 ELYRKGVSP...KKQGIDERE
14-39 ELYRKGVSP...KQGIDEREI
15-24 LYRKGVSPEQ
15-26 LYRKGVSPEQVK
15-28 LYRKGVSPEQVKKI
15-29 LYRKGVSPEQVKKIL
15-31 LYRKGVSPEQVKKILKK
15-32 LYRKGVSPEQVKKILKKQ
15-35 LYRKGVSPE...KILKKQGID
15-36 LYRKGVSPE...ILKKQGIDE
15-38 LYRKGVSPE...KKQGIDERE
15-40 LYRKGVSPE...QGIDEREIE
15-41 LYRKGVSPE...GIDEREIEK
16-24 YRKGVSPEQ
16-26 YRKGVSPEQVK
16-27 YRKGVSPEQVKK
16-28 YRKGVSPEQVKKI
16-29 YRKGVSPEQVKKIL
16-31 YRKGVSPEQVKKILKK
16-32 YRKGVSPEQVKKILKKQ
16-35 YRKGVSPEQVKKILKKQGID
16-36 YRKGVSPEQ...ILKKQGIDE
16-37 YRKGVSPEQ...LKKQGIDER
16-38 YRKGVSPEQ...KKQGIDERE
16-39 YRKGVSPEQ...KQGIDEREI
16-40 YRKGVSPEQ...QGIDEREIE
16-41 YRKGVSPEQ...GIDEREIEK
17-28 RKGVSPEQVKKI
18-30 KGVSPEQVKKILK
18-40 KGVSPEQVK...QGIDEREIE
19-26 GVSPEQVK
19-29 GVSPEQVKKIL
19-31 GVSPEQVKKILKK
19-41 GVSPEQVKK...GIDEREIEK
19-47 GVSPEQVKK...IEKVLRRIG
25-36 VKKILKKQGIDE
26-38 KKILKKQGIDERE
28-36 ILKKQGIDE
28-38 ILKKQGIDERE
28-41 ILKKQGIDEREIEK
28-43 ILKKQGIDEREIEKVL
28-47 ILKKQGIDEREIEKVLRRIG
29-42 LKKQGIDEREIEKV
30-38 KKQGIDERE
30-41 KKQGIDEREIEK
30-43 KKQGIDEREIEKVL
30-47 KKQGIDEREIEKVLRRIG
31-40 KQGIDEREIE
32-38 QGIDERE
32-40 QGIDEREIE
32-41 QGIDEREIEK
32-43 QGIDEREIEKVL
32-47 QGIDEREIEKVLRRIG
33-40 GIDEREIE
33-41 GIDEREIEK
33-47 GIDEREIEKVLRRIG
36-43 EREIEKVL
36-47 EREIEKVLRRIG
37-42 REIEKV
37-43 REIEKVL
37-45 REIEKVLRR
37-47 REIEKVLRRIG
38-43 EIEKVL
38-44 EIEKVLR
38-47 EIEKVLRRIG
39-43 IEKVL
39-44 IEKVLR
39-47 IEKVLRRIG
40-46 EKVLRRI
40-47 EKVLRRIG
41-47 KVLRRIG
42-47 VLRRIG
43-47 LRRIG
Expt.
PFNet
Deuterons
Time (seconds)
