# Supplementary material for: A machine learning method for calculating highly localized protein stabilities
Source: Nat Commun. 2026 Sep 1;17:10414. doi: 10.1038/s41467-026-75590-9 (PMC13631364; doi:10.1038/s41467-026-75590-9)
Supplement: Supplementary file 6 [file 41467_2026_75590_MOESM4_ESM.zip › Supplementary Data 2/PFNet_uptake_spike_protein_2P_RBD_ACE_0.pdf]

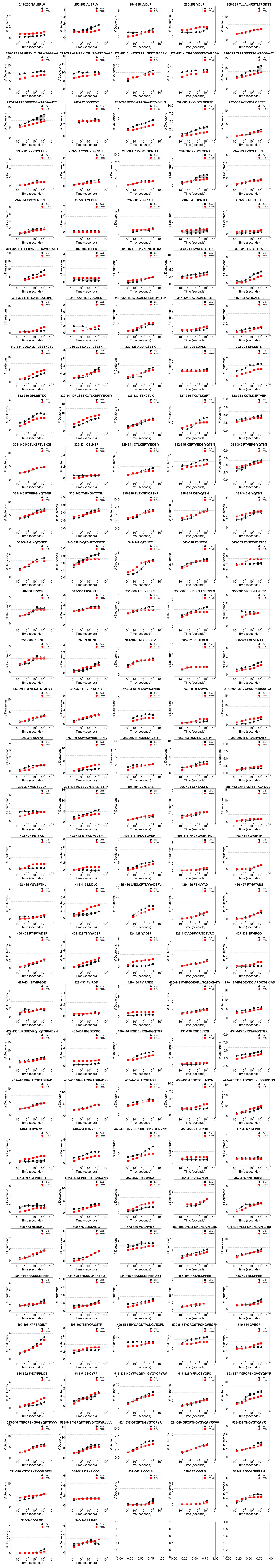

249-255 SALEPLV
250-255 ALEPLV
254-258 LVDLP
255-259 VDLPI
268-283 TLLALHRSYLTPGDSS
270-292 LALHRSYLT...SGWTAGAAA
271-292 ALHRSYLTP...SGWTAGAAA
271-293 ALHRSYLTP...GWTAGAAAY
276-292 YLTPGDSSSGWTAGAAA
276-293 YLTPGDSSSGWTAGAAAY
277-294 LTPGDSSSGWTAGAAAYY
282-287 SSSGWT
282-299 SSSGWTAGAAAYYVGYLQ
292-303 AYYVGYLQPRTF
292-305 AYYVGYLQPRTFLL
293-301 YYVGYLQPR
293-303 YYVGYLQPRTF
293-304 YYVGYLQPRTFL
294-302 YVGYLQPRT
294-303 YVGYLQPRTF
294-304 YVGYLQPRTFL
297-301 YLQPR
297-303 YLQPRTF
298-304 LQPRTFL
299-305 QPRTFLL
301-322 RTFLLKYNE...TDAVDCALD
302-306 TFLLK
302-316 TFLLKYNENGTITDA
304-315 LLKYNENGTITD
309-316 ENGTITDA
311-324 GTITDAVDCALDPL
313-322 ITDAVDCALD
313-332 ITDAVDCALDPLSETKCTLK
315-325 DAVDCALDPLS
316-324 AVDCALDPL
317-331 VDCALDPLSETKCTL
319-328 CALDPLSETK
320-328 ALDPLSETK
321-325 LDPLS
322-328 DPLSETK
322-329 DPLSETKC
322-341 DPLSETKCTLKSFTVEKGIY
326-332 ETKCTLK
327-335 TKCTLKSFT
328-338 KCTLKSFTVEK
328-340 KCTLKSFTVEKGI
329-334 CTLKSF
329-341 CTLKSFTVEKGIY
332-345 KSFTVEKGIYQTSN
334-345 FTVEKGIYQTSN
334-346 FTVEKGIYQTSNF
335-345 TVEKGIYQTSN
335-346 TVEKGIYQTSNF
338-345 KGIYQTSN
339-345 GIYQTSN
339-347 GIYQTSNFR
340-352 IYQTSNFRVQPTE
342-347 QTSNFR
343-348 TSNFRV
343-353 TSNFRVQPTES
346-350 FRVQP
346-353 FRVQPTES
351-360 TESIVRFPNI
353-367 SIVRFPNITNLCPFG
355-365 VRFPNITNLCP
356-360 RFPNI
359-363 NITNL
361-369 TNLCPFGEV
365-371 PFGEVFN
366-373 FGEVFNAT
366-379 FGEVFNATRFASVY
367-376 GEVFNATRFA
372-384 ATRFASVYAWNRK
374-380 RFASVYA
375-392 FASVYAWNRKRISNCVAD
376-380 ASVYA
376-389 ASVYAWNRKRISNC
382-392 NRKRISNCVAD
383-393 RKRISNCVADY
386-397 ISNCVADYSVLY
390-397 VADYSVLY
391-406 ADYSVLYNSASFSTFK
395-401 VLYNSAS
396-404 LYNSASFST
396-412 LYNSASFSTFKCYGVSP
402-407 FSTFKC
403-412 STFKCYGVSP
404-413 TFKCYGVSPT
405-415 FKCYGVSPTKL
408-414 YGVSPTK
408-415 YGVSPTKL
415-419 LNDLC
415-430 LNDLCFTNVYADSFVI
420-426 FTNVYAD
420-427 FTNVYADS
420-428 FTNVYADSF
421-428 TNVYADSF
424-428 YADSF
425-437 ADSFVIRGDEVRQ
427-433 SFVIRGD
427-434 SFVIRGDE
428-433 FVIRGD
428-434 FVIRGDE
428-449 FVIRGDEVR...GQTGKIADY
429-448 VIRGDEVRQIAPGQTGKIAD
429-450 VIRGDEVRQ...QTGKIADYN
430-437 IRGDEVRQ
430-446 IRGDEVRQIAPGQTGKI
431-438 RGDEVRQI
434-445 EVRQIAPGQTGK
435-448 VRQIAPGQTGKIAD
435-450 VRQIAPGQTGKIADYN
437-445 QIAPGQTGK
439-450 APGQTGKIADYN
443-476 TGKIADYNY...NLDSKVGGN
448-453 DYNYKL
448-454 DYNYKLP
449-479 YNYKLPDDF...SKVGGNYNY
450-456 NYKLPDD
451-456 YKLPDD
451-459 YKLPDDFTG
452-466 KLPDDFTGCVIAWNS
457-464 FTGCVIAW
461-467 VIAWNSN
467-474 NNLDSKVG
468-473 NLDSKV
469-475 LDSKVGG
473-479 VGGNYNY
480-495 LYRLFRKSNLKPFERD
481-496 YRLFRKSNLKPFERDI
484-494 FRKSNLKPFER
484-495 FRKSNLKPFERD
484-498 FRKSNLKPFERDIST
485-494 RKSNLKPFER
488-494 NLKPFER
490-498 KPFERDIST
498-507 TEIYQAGSTP
499-515 EIYQAGSTPCNGVEGFN
500-515 IYQAGSTPCNGVEGFN
510-514 GVEGF
514-522 FNCYFPLQS
515-519 NCYFP
515-538 NCYFPLQSY...GVGYQPYRV
517-526 YFPLQSYGFQ
523-537 YGFQPTNGVGYQPYR
523-540 YGFQPTNGVGYQPYRVVV
523-541 YGFQPTNGVGYQPYRVVVL
524-537 GFQPTNGVGYQPYR
524-540 GFQPTNGVGYQPYRVVV
528-537 TNGVGYQPYR
531-546 VGYQPYRVVVLSFELL
534-541 QPYRVVVL
537-542 RVVVLS
538-542 VVVLS
538-547 VVVLSFELLH
539-543 VVLSF
545-549 LLHAP
Expt.
PFNet
Deuterons
Time (seconds)
