# Supplementary material for: A machine learning method for calculating highly localized protein stabilities
Source: Nat Commun. 2026 Sep 1;17:10414. doi: 10.1038/s41467-026-75590-9 (PMC13631364; doi:10.1038/s41467-026-75590-9)
Supplement: Supplementary file 6 [file 41467_2026_75590_MOESM4_ESM.zip › Supplementary Data 2/PFNet_uptake_EEHEEEEHEE_rd4_APO_0.pdf]

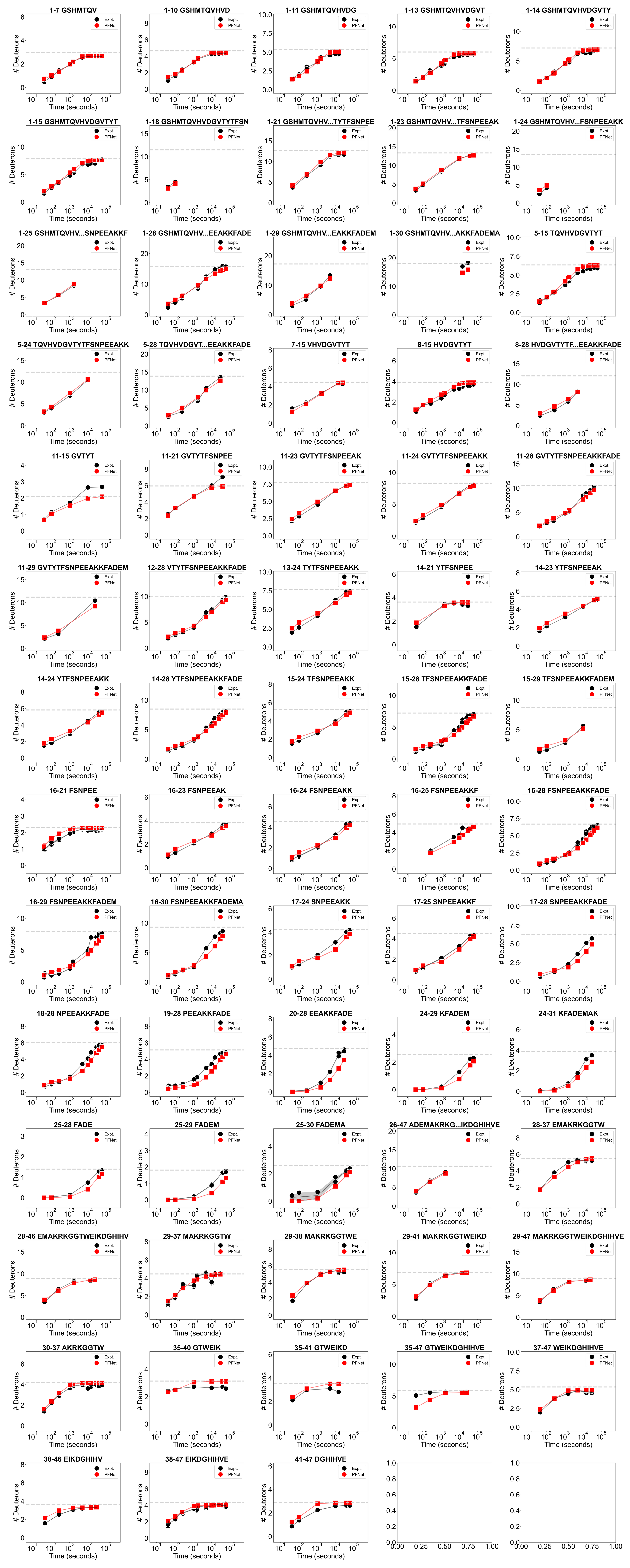

1-7 GSHMTQV
1-10 GSHMTQVHVD
1-11 GSHMTQVHVDG
1-13 GSHMTQVHVDGVT
1-14 GSHMTQVHVDGVTY
1-15 GSHMTQVHVDGVTYT
1-18 GSHMTQVHVDGVTYTFSN
1-21 GSHMTQVHV...TYTFSNPEE
1-23 GSHMTQVHV...TFSNPEEAK
1-24 GSHMTQVHV...FSNPEEAKK
1-25 GSHMTQVHV...SNPEEAKKF
1-28 GSHMTQVHV...EEAKKFADE
1-29 GSHMTQVHV...EAKKFADEM
1-30 GSHMTQVHV...AKKFADEMA
5-15 TQVHVDGVTYT
5-24 TQVHVDGVTYTFSNPEEAKK
5-28 TQVHVDGVT...EEAKKFADE
7-15 VHVDGVTYT
8-15 HVDGVTYT
8-28 HVDGVTYTF...EEAKKFADE
11-15 GVTYT
11-21 GVTYTFSNPEE
11-23 GVTYTFSNPEEAK
11-24 GVTYTFSNPEEAKK
11-28 GVTYTFSNPEEAKKFADE
11-29 GVTYTFSNPEEAKKFADEM
12-28 VTYTFSNPEEAKKFADE
13-24 TYTFSNPEEAKK
14-21 YTFSNPEE
14-23 YTFSNPEEAK
14-24 YTFSNPEEAKK
14-28 YTFSNPEEAKKFADE
15-24 TFSNPEEAKK
15-28 TFSNPEEAKKFADE
15-29 TFSNPEEAKKFADEM
16-21 FSNPEE
16-23 FSNPEEAK
16-24 FSNPEEAKK
16-25 FSNPEEAKKF
16-28 FSNPEEAKKFADE
16-29 FSNPEEAKKFADEM
16-30 FSNPEEAKKFADEMA
17-24 SNPEEAKK
17-25 SNPEEAKKF
17-28 SNPEEAKKFADE
18-28 NPEEAKKFADE
19-28 PEEAKKFADE
20-28 EEAKKFADE
24-29 KFADEM
24-31 KFADEMAK
25-28 FADE
25-29 FADEM
25-30 FADEMA
26-47 ADEMAKRKG...IKDGHIHVE
28-37 EMAKRKGGTW
28-46 EMAKRKGGTWEIKDGHIHV
29-37 MAKRKGGTW
29-38 MAKRKGGTWE
29-41 MAKRKGGTWEIKD
29-47 MAKRKGGTWEIKDGHIHVE
30-37 AKRKGGTW
35-40 GTWEIK
35-41 GTWEIKD
35-47 GTWEIKDGHIHVE
37-47 WEIKDGHIHVE
38-46 EIKDGHIHV
38-47 EIKDGHIHVE
41-47 DGHIHVE
Expt.
PFNet
Deuterons
Time (seconds)
