# Supplementary material for: A machine learning method for calculating highly localized protein stabilities
Source: Nat Commun. 2026 Sep 1;17:10414. doi: 10.1038/s41467-026-75590-9 (PMC13631364; doi:10.1038/s41467-026-75590-9)
Supplement: Supplementary file 6 [file 41467_2026_75590_MOESM4_ESM.zip › Supplementary Data 2/PFNet_uptake_T4L_0.pdf]

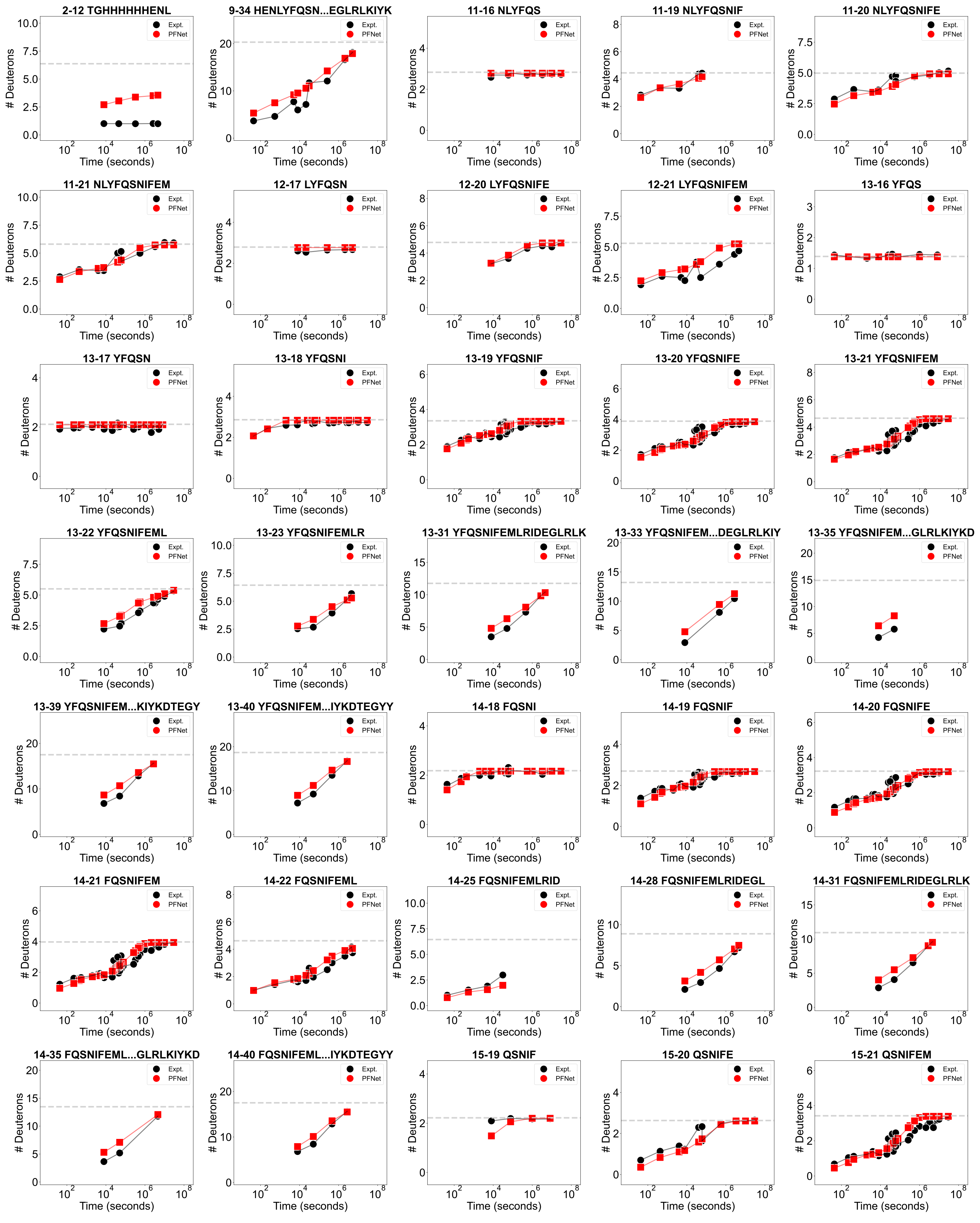
2-12 TGHHHHHHENL
9-34 HENLYFQSN...EGLRLKIYK
11-16 NLYFQS
11-19 NLYFQSNIF
11-20 NLYFQSNIFE
11-21 NLYFQSNIFEM
12-17 LYFQSN
12-20 LYFQSNIFE
12-21 LYFQSNIFEM
13-16 YFQS
13-17 YFQSN
13-18 YFQSNI
13-19 YFQSNIF
13-20 YFQSNIFE
13-21 YFQSNIFEM
13-22 YFQSNIFEML
13-23 YFQSNIFEMLR
13-31 YFQSNIFEMLRIDEGLRLK
13-33 YFQSNIFEM...DEGLRLKIY
13-35 YFQSNIFEM...GLRLKIYKD
13-39 YFQSNIFEM...KIYKDTEGY
13-40 YFQSNIFEM...IYKDTEGYY
14-18 FQSNI
14-19 FQSNIF
14-20 FQSNIFE
14-21 FQSNIFEM
14-22 FQSNIFEML
14-25 FQSNIFEMLRID
14-28 FQSNIFEMLRIDEGL
14-31 FQSNIFEMLRIDEGLRLK
14-35 FQSNIFEML...GLRLKIYKD
14-40 FQSNIFEML...IYKDTEGYY
15-19 QSNIF
15-20 QSNIFE
15-21 QSNIFEM
Expt.
PFNet
Deuterons
Time (seconds)

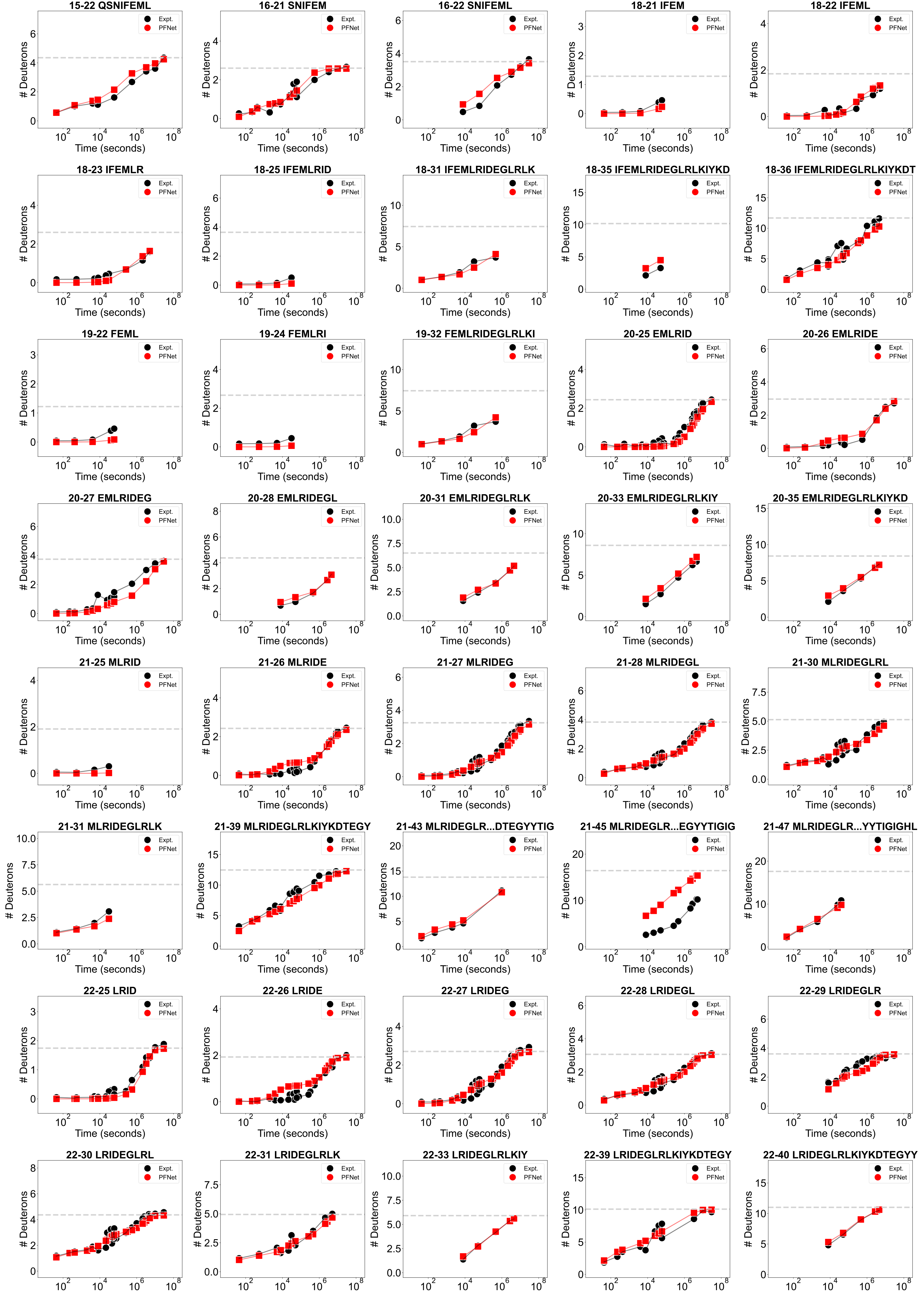
15-22 QSNIFEML
16-21 SNIFEM
16-22 SNIFEML
18-21 IFEM
18-22 IFEML
18-23 IFEMLR
18-25 IFEMLRID
18-31 IFEMLRIDEGLRLK
18-35 IFEMLRIDEGLRLKIYKD
18-36 IFEMLRIDEGLRLKIYKDT
19-22 FEML
19-24 FEMLRI
19-32 FEMLRIDEGLRLKI
20-25 EMLRID
20-26 EMLRIDE
20-27 EMLRIDEG
20-28 EMLRIDEGL
20-31 EMLRIDEGLRLK
20-33 EMLRIDEGLRLKIY
20-35 EMLRIDEGLRLKIYKD
21-25 MLRID
21-26 MLRIDE
21-27 MLRIDEG
21-28 MLRIDEGL
21-30 MLRIDEGLRL
21-31 MLRIDEGLRLK
21-39 MLRIDEGLRLKIYKDTEGY
21-43 MLRIDEGLR...DTEGYYTIG
21-45 MLRIDEGLR...EGYYTIGIG
21-47 MLRIDEGLR...YYTIGIGHL
22-25 LRID
22-26 LRIDE
22-27 LRIDEG
22-28 LRIDEGL
22-29 LRIDEGLR
22-30 LRIDEGLRL
22-31 LRIDEGLRLK
22-33 LRIDEGLRLKIY
22-39 LRIDEGLRLKIYKDTEGY
22-40 LRIDEGLRLKIYKDTEGYY
Expt.
PFNet
Deuterons
Time (seconds)

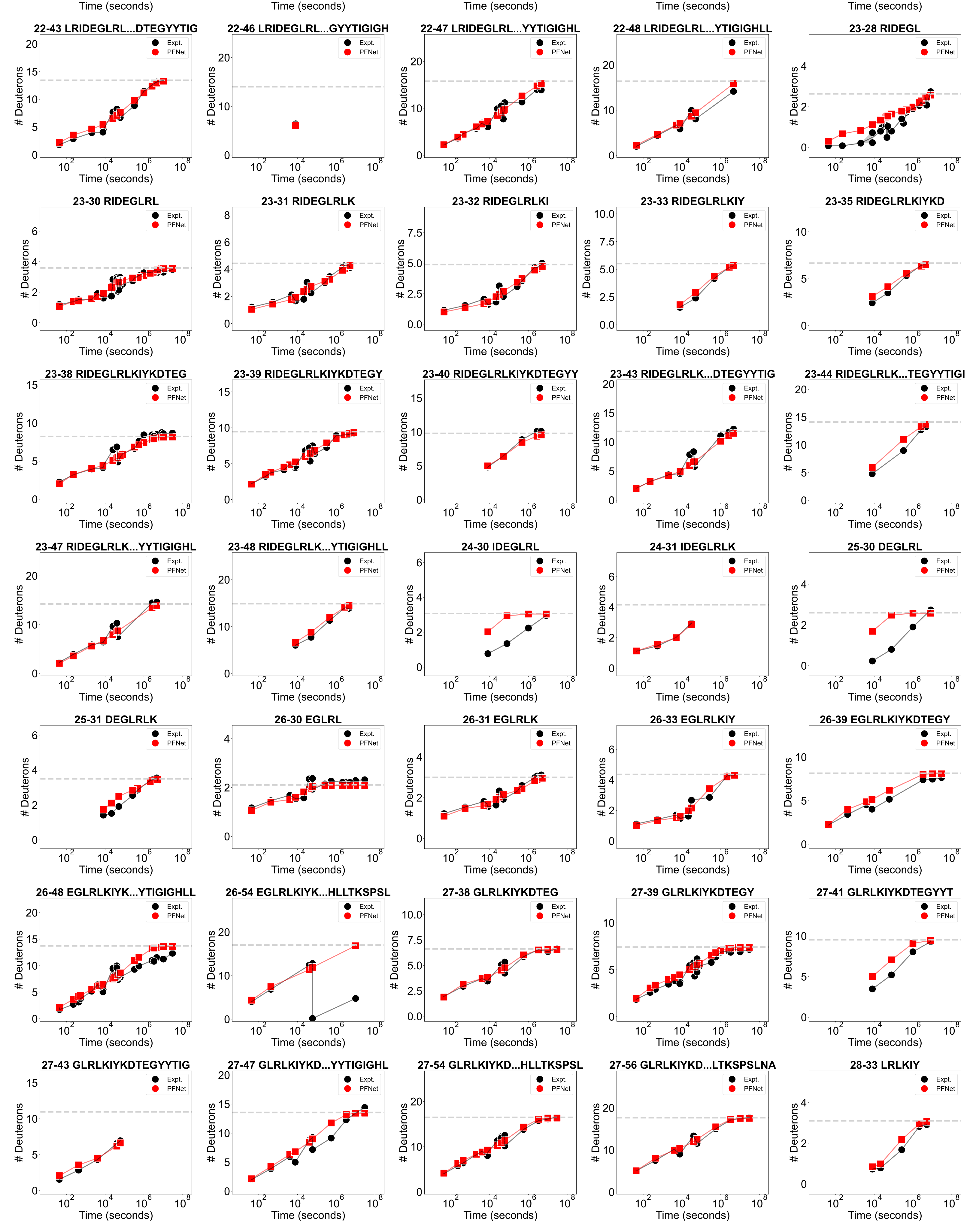

Time (seconds)
22-43 LRIDEGLRL...DTEGYYTIG
22-46 LRIDEGLRL...GYYTIGIGH
22-47 LRIDEGLRL...YYTIGIGHL
22-48 LRIDEGLRL...YTIGIGHLL
23-28 RIDEGL
23-30 RIDEGLRL
23-31 RIDEGLRLK
23-32 RIDEGLRLKI
23-33 RIDEGLRLKIY
23-35 RIDEGLRLKIYKD
23-38 RIDEGLRLKIYKDTEG
23-39 RIDEGLRLKIYKDTEGY
23-40 RIDEGLRLKIYKDTEGYY
23-43 RIDEGLRLK...DTEGYYTIG
23-44 RIDEGLRLK...TEGYYTIGI
23-47 RIDEGLRLK...YYTIGIGHL
23-48 RIDEGLRLK...YTIGIGHLL
24-30 IDEGLRL
24-31 IDEGLRLK
25-30 DEGLRL
25-31 DEGLRLK
26-30 EGLRL
26-31 EGLRLK
26-33 EGLRLKIY
26-39 EGLRLKIYKDTEGY
26-48 EGLRLKIYK...YTIGIGHLL
26-54 EGLRLKIYK...HLLTKSPSL
27-38 GLRLKIYKDTEG
27-39 GLRLKIYKDTEGY
27-41 GLRLKIYKDTEGYYT
27-43 GLRLKIYKDTEGYYTIG
27-47 GLRLKIYKD...YYTIGIGHL
27-54 GLRLKIYKD...HLLTKSPSL
27-56 GLRLKIYKD...LTKSPSLNA
28-33 LRLKIY
Expt.
PFNet
Deuterons
Time (seconds)

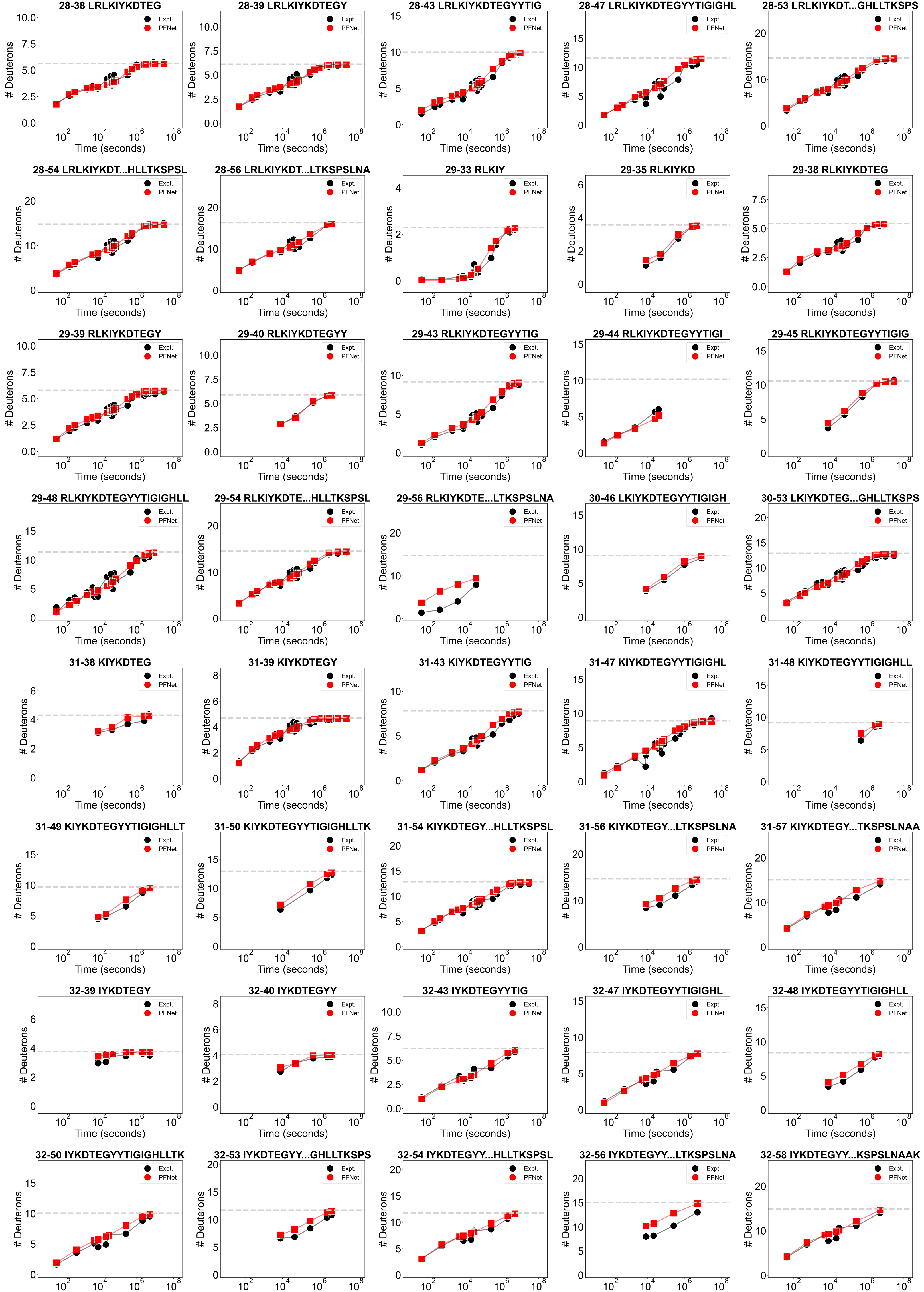
28-38 LRLKIYKDTEG
28-39 LRLKIYKDTEGY
28-43 LRLKIYKDTEGYYTIG
28-47 LRLKIYKDTEGYYTIGIGHL
28-53 LRLKIYKDT...GHLLTKSPS
28-54 LRLKIYKDT...HLLTKSPSL
28-56 LRLKIYKDT...LTKSPSLNA
29-33 RLKIY
29-35 RLKIYKD
29-38 RLKIYKDTEG
29-39 RLKIYKDTEGY
29-40 RLKIYKDTEGYY
29-43 RLKIYKDTEGYYTIG
29-44 RLKIYKDTEGYYTIGI
29-45 RLKIYKDTEGYYTIGIG
29-48 RLKIYKDTEGYYTIGIGHLL
29-54 RLKIYKDTE...HLLTKSPSL
29-56 RLKIYKDTE...LTKSPSLNA
30-46 LKIYKDTEGYYTIGIGH
30-53 LKIYKDTEG...GHLLTKSPS
31-38 KIYKDTEG
31-39 KIYKDTEGY
31-43 KIYKDTEGYYTIG
31-47 KIYKDTEGYYTIGIGHL
31-48 KIYKDTEGYYTIGIGHLL
31-49 KIYKDTEGYYTIGIGHLLT
31-50 KIYKDTEGYYTIGIGHLLTK
31-54 KIYKDTEGY...HLLTKSPSL
31-56 KIYKDTEGY...LTKSPSLNA
31-57 KIYKDTEGY...TKSPSLNAA
32-39 IYKDTEGY
32-40 IYKDTEGYY
32-43 IYKDTEGYYTIG
32-47 IYKDTEGYYTIGIGHL
32-48 IYKDTEGYYTIGIGHLL
32-50 IYKDTEGYYTIGIGHLLTK
32-53 IYKDTEGYY...GHLLTKSPS
32-54 IYKDTEGYY...HLLTKSPSL
32-56 IYKDTEGYY...LTKSPSLNA
32-58 IYKDTEGYY...KSPSLNAAK
Expt.
PFNet
Deuterons
Time (seconds)

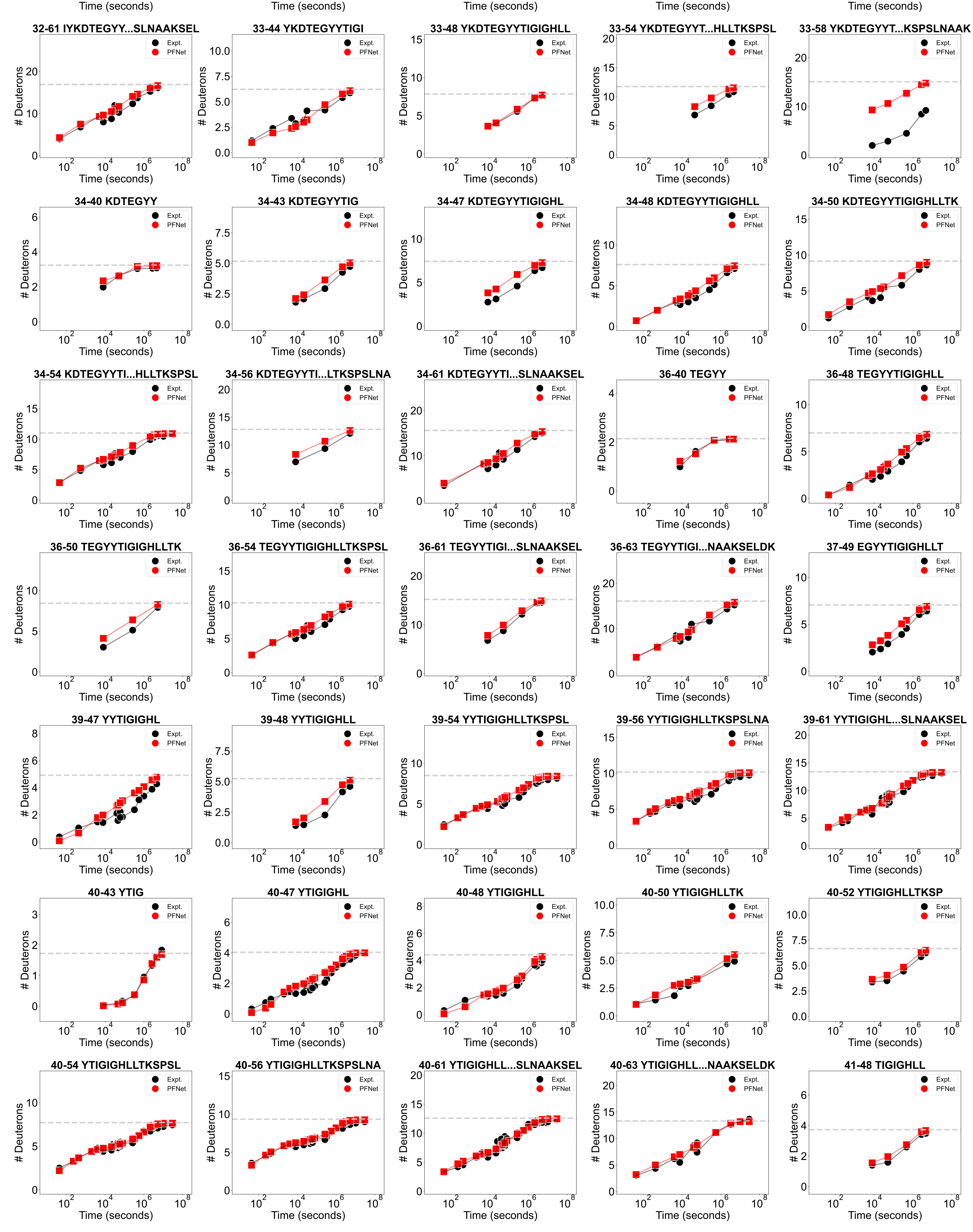
Time (seconds)
32-61 IYKDTEGYY...SLNAAKSEL
33-44 YKDTEGYYTIGI
33-48 YKDTEGYYTIGIGHLL
33-54 YKDTEGYYT...HLLTKSPSL
33-58 YKDTEGYYT...KSPSLNAAK
34-40 KDTEGYY
34-43 KDTEGYYTIG
34-47 KDTEGYYTIGIGHL
34-48 KDTEGYYTIGIGHLL
34-50 KDTEGYYTIGIGHLLTK
34-54 KDTEGYYTI...HLLTKSPSL
34-56 KDTEGYYTI...LTKSPSLNA
34-61 KDTEGYYTI...SLNAAKSEL
36-40 TEGYY
36-48 TEGYYTIGIGHLL
36-50 TEGYYTIGIGHLLTK
36-54 TEGYYTIGIGHLLTKSPSL
36-61 TEGYYTIGI...SLNAAKSEL
36-63 TEGYYTIGI...NAAKSELDK
37-49 EGYYTIGIGHLLT
39-47 YYTIGIGHL
39-48 YYTIGIGHLL
39-54 YYTIGIGHLLTKSPSL
39-56 YYTIGIGHLLTKSPSLNA
39-61 YYTIGIGHL...SLNAAKSEL
40-43 YTIG
40-47 YTIGIGHL
40-48 YTIGIGHLL
40-50 YTIGIGHLLTK
40-52 YTIGIGHLLTKSP
40-54 YTIGIGHLLTKSPSL
40-56 YTIGIGHLLTKSPSLNA
40-61 YTIGIGHLL...SLNAAKSEL
40-63 YTIGIGHLL...NAAKSELDK
41-48 TIGIGHLL
Expt.
PFNet
Deuterons
Time (seconds)

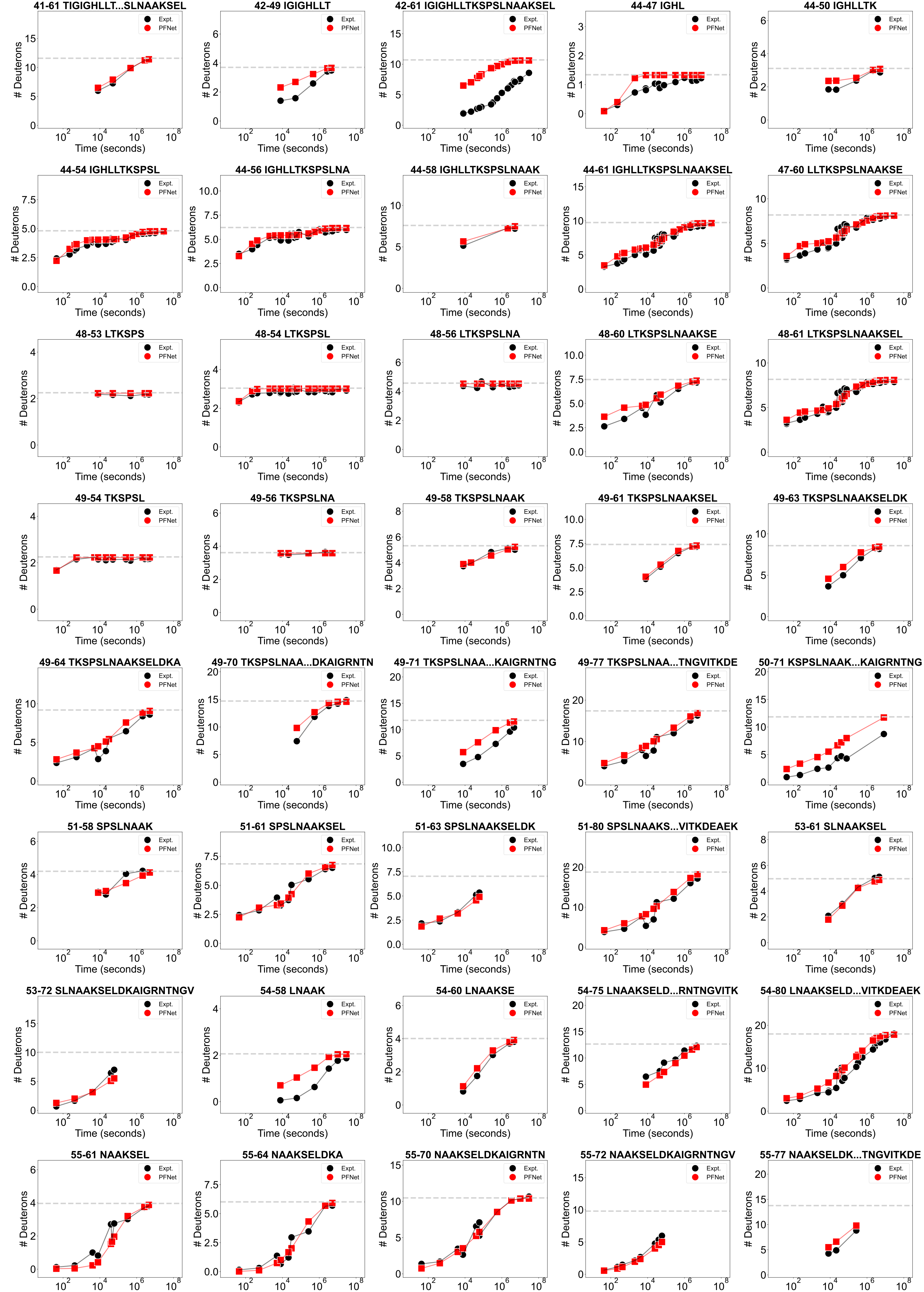

41-61 TIGIGHLLT...SLNAAKSEL
42-49 IGIGHLLT
42-61 IGIGHLLTKSPSLNAAKSEL
44-47 IGHL
44-50 IGHLLTK
44-54 IGHLLTKSPSL
44-56 IGHLLTKSPSLNA
44-58 IGHLLTKSPSLNAAK
44-61 IGHLLTKSPSLNAAKSEL
47-60 LLTKSPSLNAAKSE
48-53 LTKSPS
48-54 LTKSPSL
48-56 LTKSPSLNA
48-60 LTKSPSLNAAKSE
48-61 LTKSPSLNAAKSEL
49-54 TKSPSL
49-56 TKSPSLNA
49-58 TKSPSLNAAK
49-61 TKSPSLNAAKSEL
49-63 TKSPSLNAAKSELDK
49-64 TKSPSLNAAKSELDKA
49-70 TKSPSLNAA...DKAIGRNTN
49-71 TKSPSLNAA...KAIGRNTNG
49-77 TKSPSLNAA...TNGVITKDE
50-71 KSPSLNAAK...KAIGRNTNG
51-58 SPSLNAAK
51-61 SPSLNAAKSEL
51-63 SPSLNAAKSELDK
51-80 SPSLNAAKS...VITKDEAEK
53-61 SLNAAKSEL
53-72 SLNAAKSELDKAIGRNTNGV
54-58 LNAAK
54-60 LNAAKSE
54-75 LNAAKSELD...RNTNGVITK
54-80 LNAAKSELD...VITKDEAEK
55-61 NAAKSEL
55-64 NAAKSELDKA
55-70 NAAKSELDKAIGRNTN
55-72 NAAKSELDKAIGRNTNGV
55-77 NAAKSELDK...TNGVITKDE
Expt.
PFNet
Deuterons
Time (seconds)

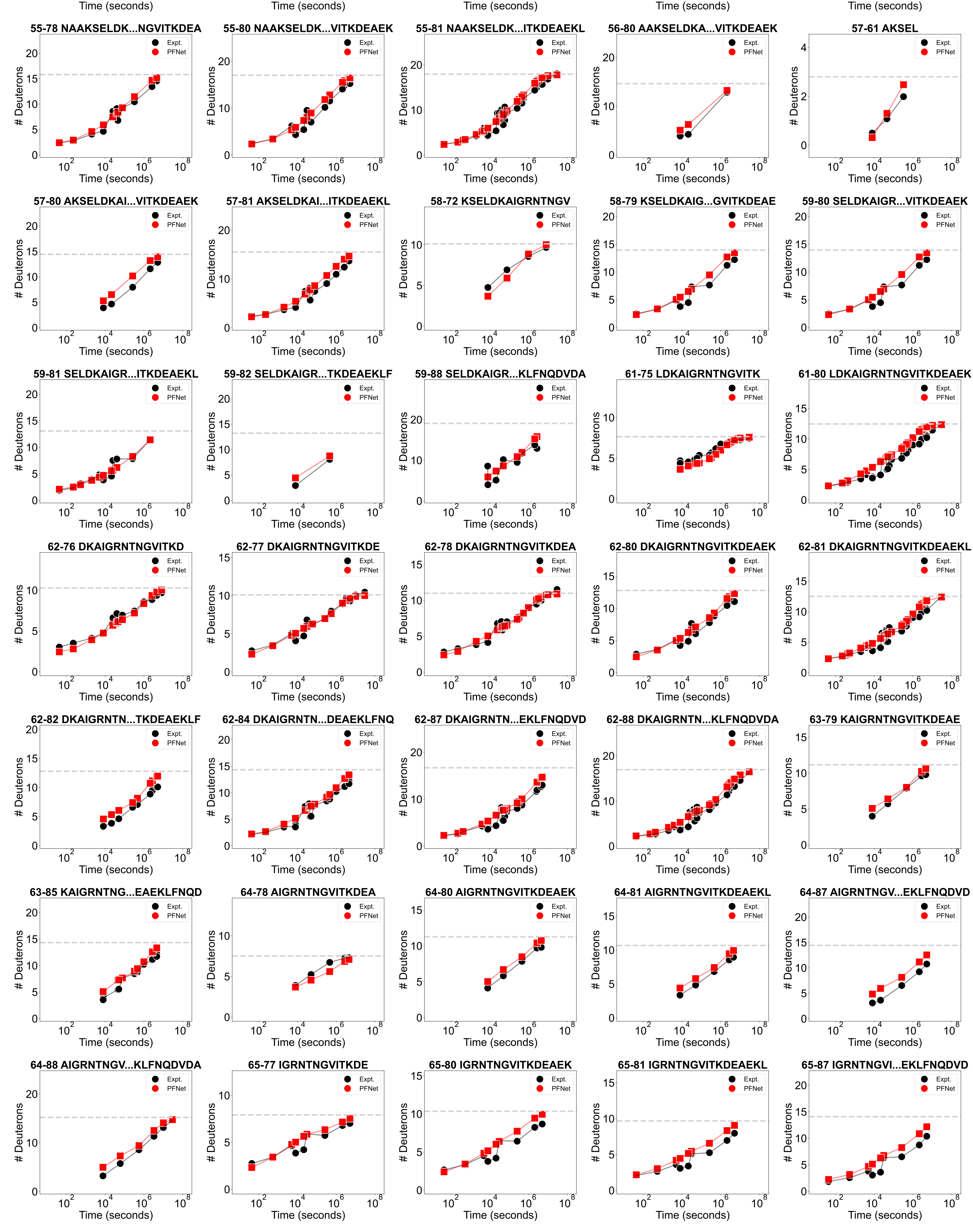
55-78 NAAKSELDK...NGVITKDEA
55-80 NAAKSELDK...VITKDEAEK
55-81 NAAKSELDK...ITKDEAEKL
56-80 AAKSELDKA...VITKDEAEK
57-61 AKSEL
57-80 AKSELDKAI...VITKDEAEK
57-81 AKSELDKAI...ITKDEAEKL
58-72 KSELDKAIGRNTNGV
58-79 KSELDKAIG...GVITKDEAE
59-80 SELDKAIGR...VITKDEAEK
59-81 SELDKAIGR...ITKDEAEKL
59-82 SELDKAIGR...TKDEAEKLF
59-88 SELDKAIGR...KLFNQDVDA
61-75 LDKAIGRNTNGVITK
61-80 LDKAIGRNTNGVITKDEAEK
62-76 DKAIGRNTNGVITKD
62-77 DKAIGRNTNGVITKDE
62-78 DKAIGRNTNGVITKDEA
62-80 DKAIGRNTNGVITKDEAEK
62-81 DKAIGRNTNGVITKDEAEKL
62-82 DKAIGRNTN...TKDEAEKLF
62-84 DKAIGRNTN...DEAEKLFNQ
62-87 DKAIGRNTN...EKLFNQDVD
62-88 DKAIGRNTN...KLFNQDVDA
63-79 KAIGRNTNGVITKDEAE
63-85 KAIGRNTNG...EAEKLFNQD
64-78 AIGRNTNGVITKDEA
64-80 AIGRNTNGVITKDEAEK
64-81 AIGRNTNGVITKDEAEKL
64-87 AIGRNTNGV...EKLFNQDVD
64-88 AIGRNTNGV...KLFNQDVDA
65-77 IGRNTNGVITKDE
65-80 IGRNTNGVITKDEAEK
65-81 IGRNTNGVITKDEAEKL
65-87 IGRNTNGVI...EKLFNQDVD
Expt.
PFNet
Deuterons
Time (seconds)

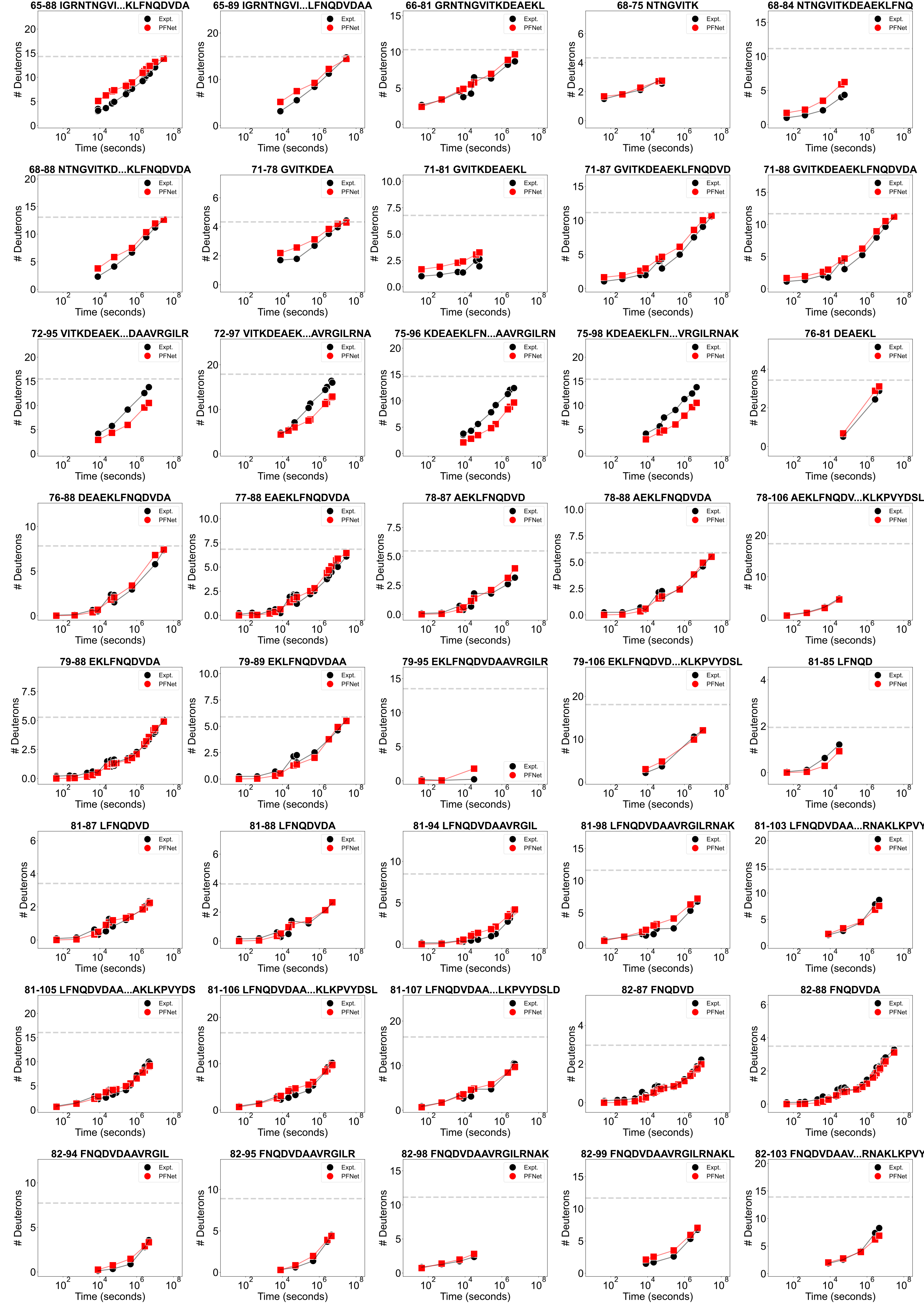
65-88 IGRNTNGVI...KLFNQDVDA
65-89 IGRNTNGVI...LFNQDVDAA
66-81 GRNTNGVITKDEAEKL
68-75 NTNGVITK
68-84 NTNGVITKDEAEKLFNQ
68-88 NTNGVITKD...KLFNQDVDA
71-78 GVITKDEA
71-81 GVITKDEAEKL
71-87 GVITKDEAEKLFNQDVD
71-88 GVITKDEAEKLFNQDVDA
72-95 VITKDEAEK...DAAVRGILR
72-97 VITKDEAEK...AVRGILRNA
75-96 KDEAEKLFN...AAVRGILRN
75-98 KDEAEKLFN...VRGILRNAK
76-81 DEAEKL
76-88 DEAEKLFNQDVDA
77-88 EAEKLFNQDVDA
78-87 AEKLFNQDVD
78-88 AEKLFNQDVDA
78-106 AEKLFNQDV...KLKPVYDSL
79-88 EKLFNQDVDA
79-89 EKLFNQDVDAA
79-95 EKLFNQDVDAAVRGILR
79-106 EKLFNQDVD...KLKPVYDSL
81-85 LFNQD
81-87 LFNQDVD
81-88 LFNQDVDA
81-94 LFNQDVDAAVRGIL
81-98 LFNQDVDAAVRGILRNAK
81-103 LFNQDVDAA...RNAKLKPVY
81-105 LFNQDVDAA...AKLKPVYDS
81-106 LFNQDVDAA...KLKPVYDSL
81-107 LFNQDVDAA...LKPVYDSLD
82-87 FNQDVD
82-88 FNQDVDA
82-94 FNQDVDAAVRGIL
82-95 FNQDVDAAVRGILR
82-98 FNQDVDAAVRGILRNAK
82-99 FNQDVDAAVRGILRNAKL
82-103 FNQDVDAAV...RNAKLKPVY
Expt.
PFNet
Deuterons
Time (seconds)
