# Supplementary material for: A machine learning method for calculating highly localized protein stabilities
Source: Nat Commun. 2026 Sep 1;17:10414. doi: 10.1038/s41467-026-75590-9 (PMC13631364; doi:10.1038/s41467-026-75590-9)
Supplement: Supplementary file 6 [file 41467_2026_75590_MOESM4_ESM.zip › Supplementary Data 2/PFNet_uptake_T4L_1.pdf]

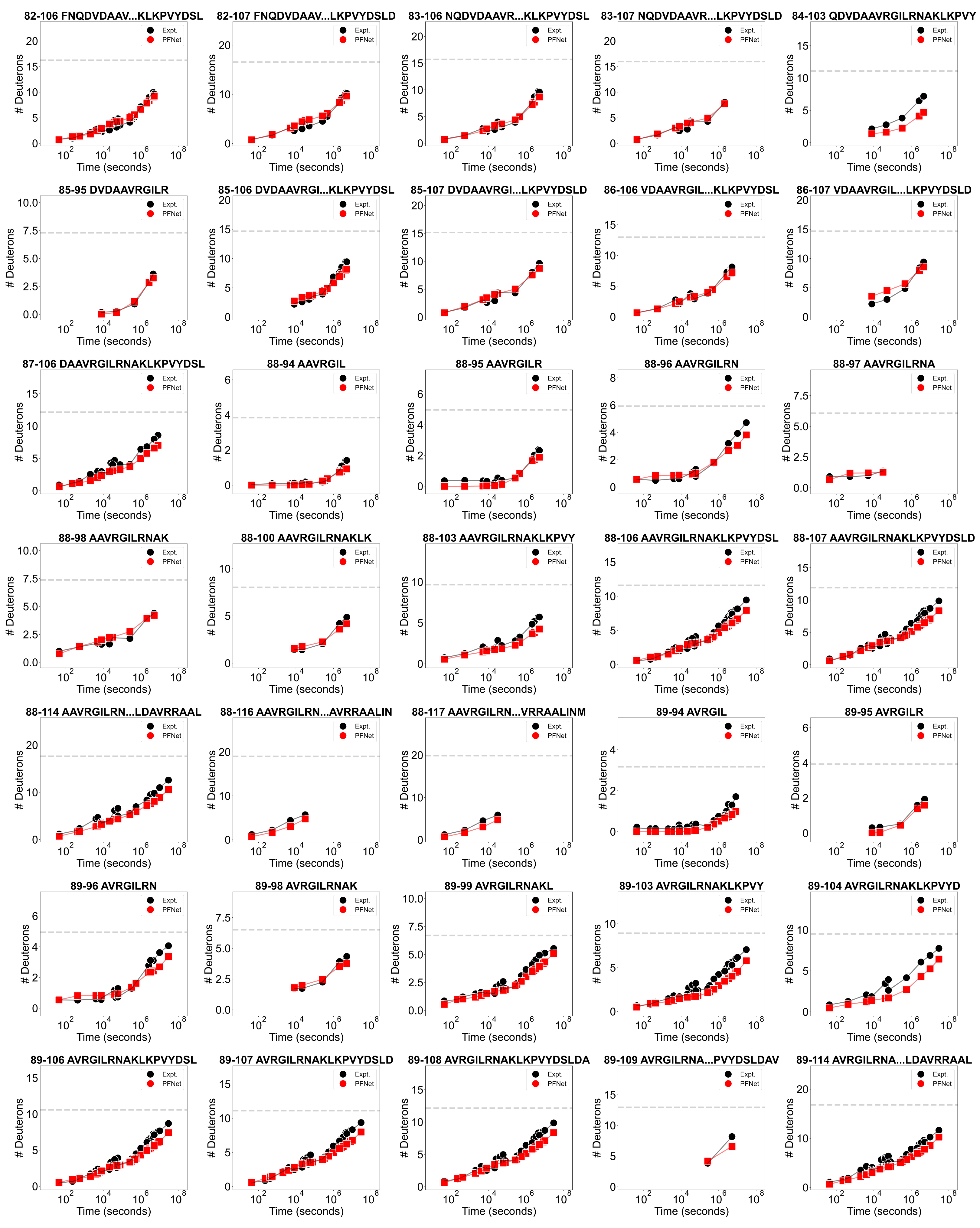
82-106 FNQDVDAAV...KLKPVYDSL
82-107 FNQDVDAAV...LKPVYDSLD
83-106 NQDVDAAVR...KLKPVYDSL
83-107 NQDVDAAVR...LKPVYDSLD
84-103 QDVDAAVRGILRNAKLKPVY
85-95 DVDAAVRGILR
85-106 DVDAAVRGI...KLKPVYDSL
85-107 DVDAAVRGI...LKPVYDSLD
86-106 VDAAVRGIL...KLKPVYDSL
86-107 VDAAVRGIL...LKPVYDSLD
87-106 DAAVRGILRNAKLKPVYDSL
88-94 AAVRGIL
88-95 AAVRGILR
88-96 AAVRGILRN
88-97 AAVRGILRNA
88-98 AAVRGILRNAK
88-100 AAVRGILRNAKLK
88-103 AAVRGILRNAKLKPVY
88-106 AAVRGILRNAKLKPVYDSL
88-107 AAVRGILRNAKLKPVYDSLD
88-114 AAVRGILRN...LDAVRRAAL
88-116 AAVRGILRN...AVRRAALIN
88-117 AAVRGILRN...VRRAALINM
89-94 AVRGIL
89-95 AVRGILR
89-96 AVRGILRN
89-98 AVRGILRNAK
89-99 AVRGILRNAKL
89-103 AVRGILRNAKLKPVY
89-104 AVRGILRNAKLKPVYD
89-106 AVRGILRNAKLKPVYDSL
89-107 AVRGILRNAKLKPVYDSLD
89-108 AVRGILRNAKLKPVYDSLDA
89-109 AVRGILRNA...PVYDSLDAV
89-114 AVRGILRNA...LDAVRRAAL
Expt.
PFNet
Deuterons
Time (seconds)

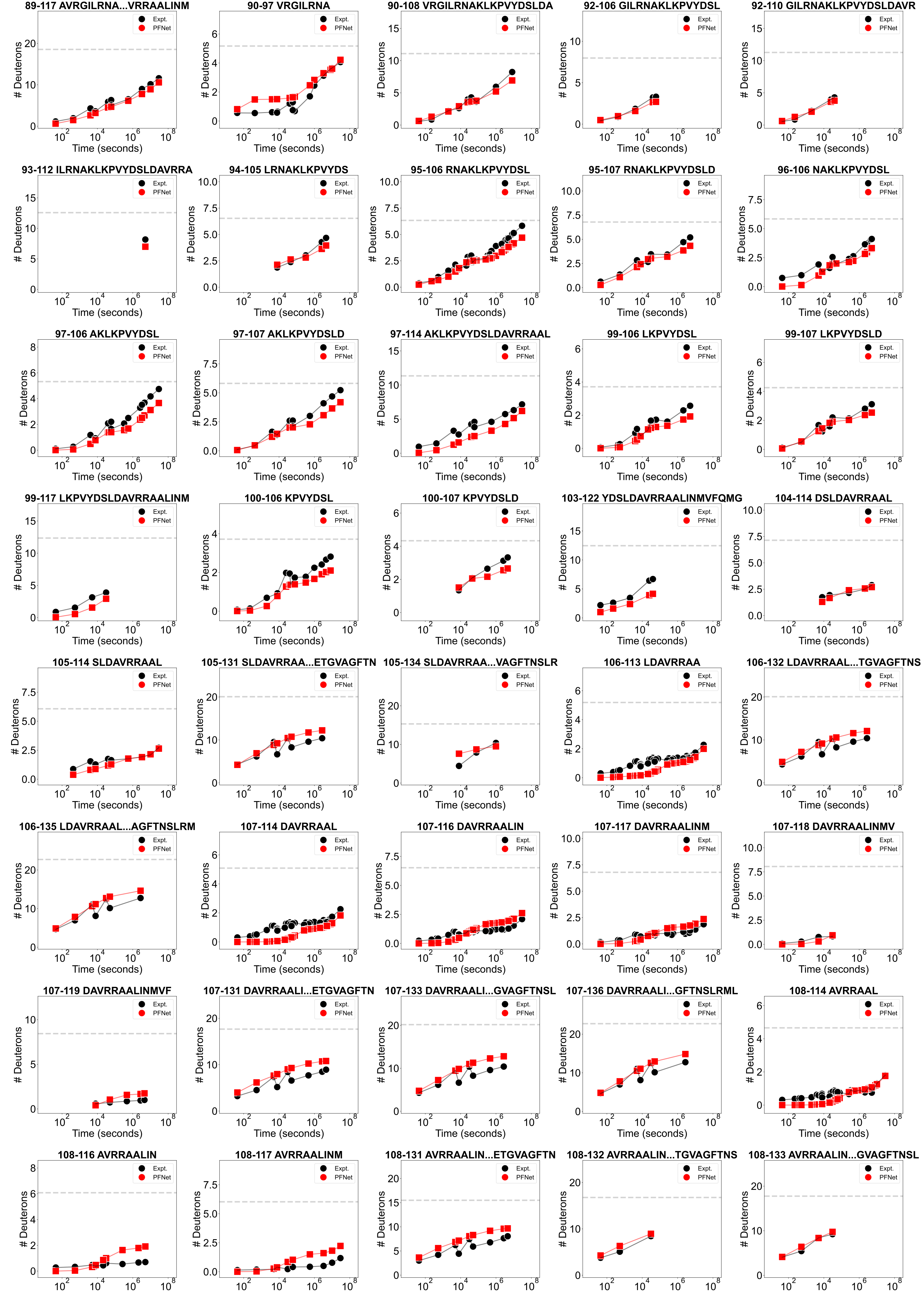
89-117 AVRGILRNA...VRRAALINM
90-97 VRGILRNA
90-108 VRGILRNAKLKPVYDSLDA
92-106 GILRNAKLKPVYDSL
92-110 GILRNAKLKPVYDSLDAVR
93-112 ILRNAKLKPVYDSLDAVRRA
94-105 LRNAKLKPVYDS
95-106 RNAKLKPVYDSL
95-107 RNAKLKPVYDSLD
96-106 NAKLKPVYDSL
97-106 AKLKPVYDSL
97-107 AKLKPVYDSLD
97-114 AKLKPVYDSLDAVRRAAL
99-106 LKPVYDSL
99-107 LKPVYDSLD
99-117 LKPVYDSLDAVRRAALINM
100-106 KPVYDSL
100-107 KPVYDSLD
103-122 YDSLDAVRRAALINMVFQMG
104-114 DSLDAVRRAAL
105-114 SLDAVRRAAL
105-131 SLDAVRRAA...ETGVAGFTN
105-134 SLDAVRRAA...VAGFTNSLR
106-113 LDAVRRAA
106-132 LDAVRRAAL...TGVAGFTNS
106-135 LDAVRRAAL...AGFTNSLRM
107-114 DAVRRAAL
107-116 DAVRRAALIN
107-117 DAVRRAALINM
107-118 DAVRRAALINMV
107-119 DAVRRAALINMVF
107-131 DAVRRAALI...ETGVAGFTN
107-133 DAVRRAALI...GVAGFTNSL
107-136 DAVRRAALI...GFTNSLRML
108-114 AVRRAAL
108-116 AVRRAALIN
108-117 AVRRAALINM
108-131 AVRRAALIN...ETGVAGFTN
108-132 AVRRAALIN...TGVAGFTNS
108-133 AVRRAALIN...GVAGFTNSL
Expt.
PFNet
Deuterons
Time (seconds)

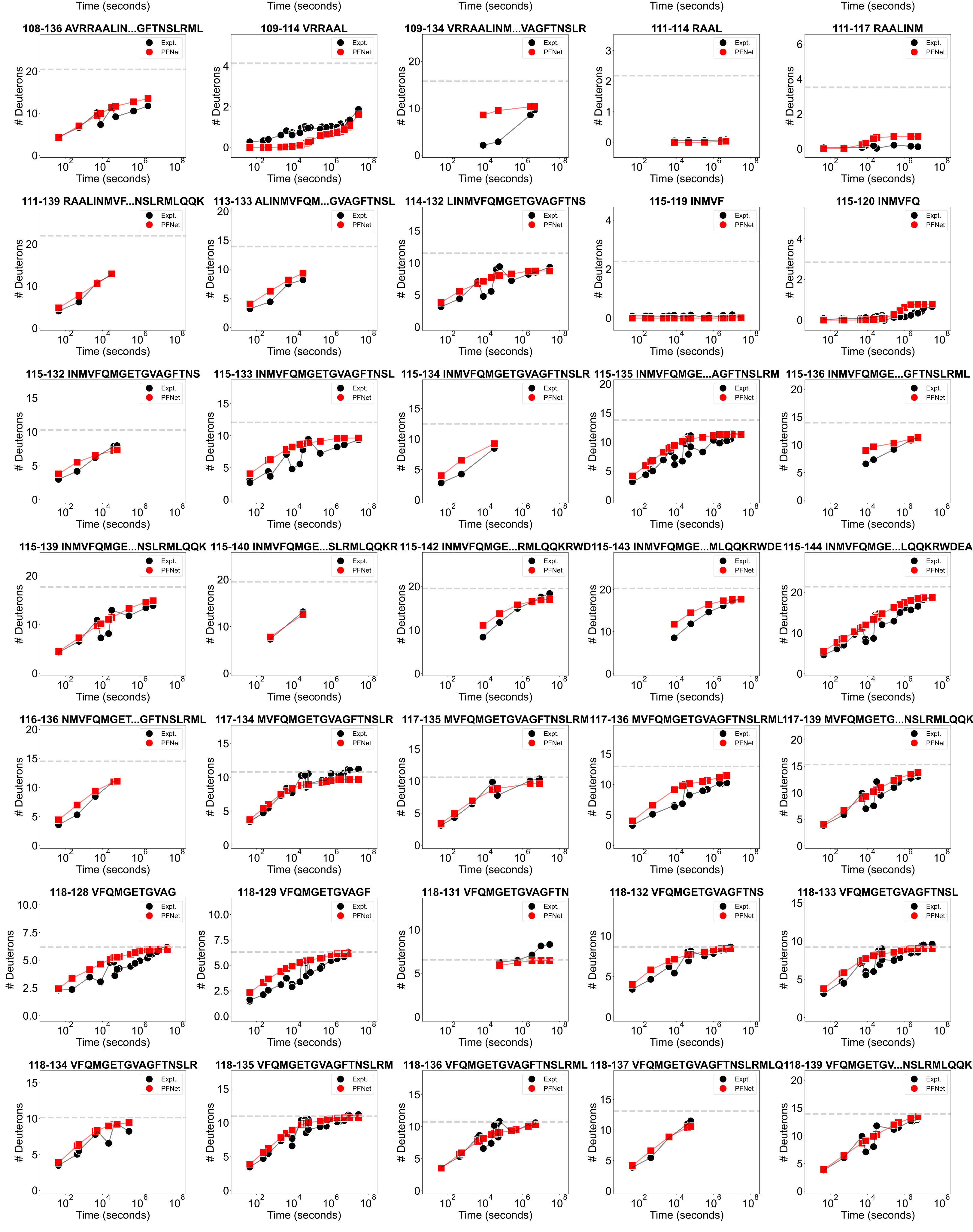
Time (seconds)
108-136 AVRRAALIN...GFTNSLRML
109-114 VRRAAL
109-134 VRRAALINM...VAGFTNSLR
111-114 RAAL
111-117 RAALINM
111-139 RAALINMVF...NSLRMLQQK
113-133 ALINMVFQM...GVAGFTNSL
114-132 LINMVFQMGETGVAGFTNS
115-119 INMVF
115-120 INMVFQ
115-132 INMVFQMGETGVAGFTNS
115-133 INMVFQMGETGVAGFTNSL
115-134 INMVFQMGETGVAGFTNSLR
115-135 INMVFQMGE...AGFTNSLRM
115-136 INMVFQMGE...GFTNSLRML
115-139 INMVFQMGE...NSLRMLQQK
115-140 INMVFQMGE...SLRMLQQKR
115-142 INMVFQMGE...RMLQQKRWD
115-143 INMVFQMGE...MLQQKRWDE
115-144 INMVFQMGE...LQQKRWDEA
116-136 NMVFQMGET...GFTNSLRML
117-134 MVFQMGETGVAGFTNSLR
117-135 MVFQMGETGVAGFTNSLRM
117-136 MVFQMGETGVAGFTNSLRML
117-139 MVFQMGETG...NSLRMLQQK
118-128 VFQMGETGVAG
118-129 VFQMGETGVAGF
118-131 VFQMGETGVAGFTN
118-132 VFQMGETGVAGFTNS
118-133 VFQMGETGVAGFTNSL
118-134 VFQMGETGVAGFTNSLR
118-135 VFQMGETGVAGFTNSLRM
118-136 VFQMGETGVAGFTNSLRML
118-137 VFQMGETGVAGFTNSLRMLQ
118-139 VFQMGETGV...NSLRMLQQK
Deuterons
Time (seconds)
Expt.
PFNet

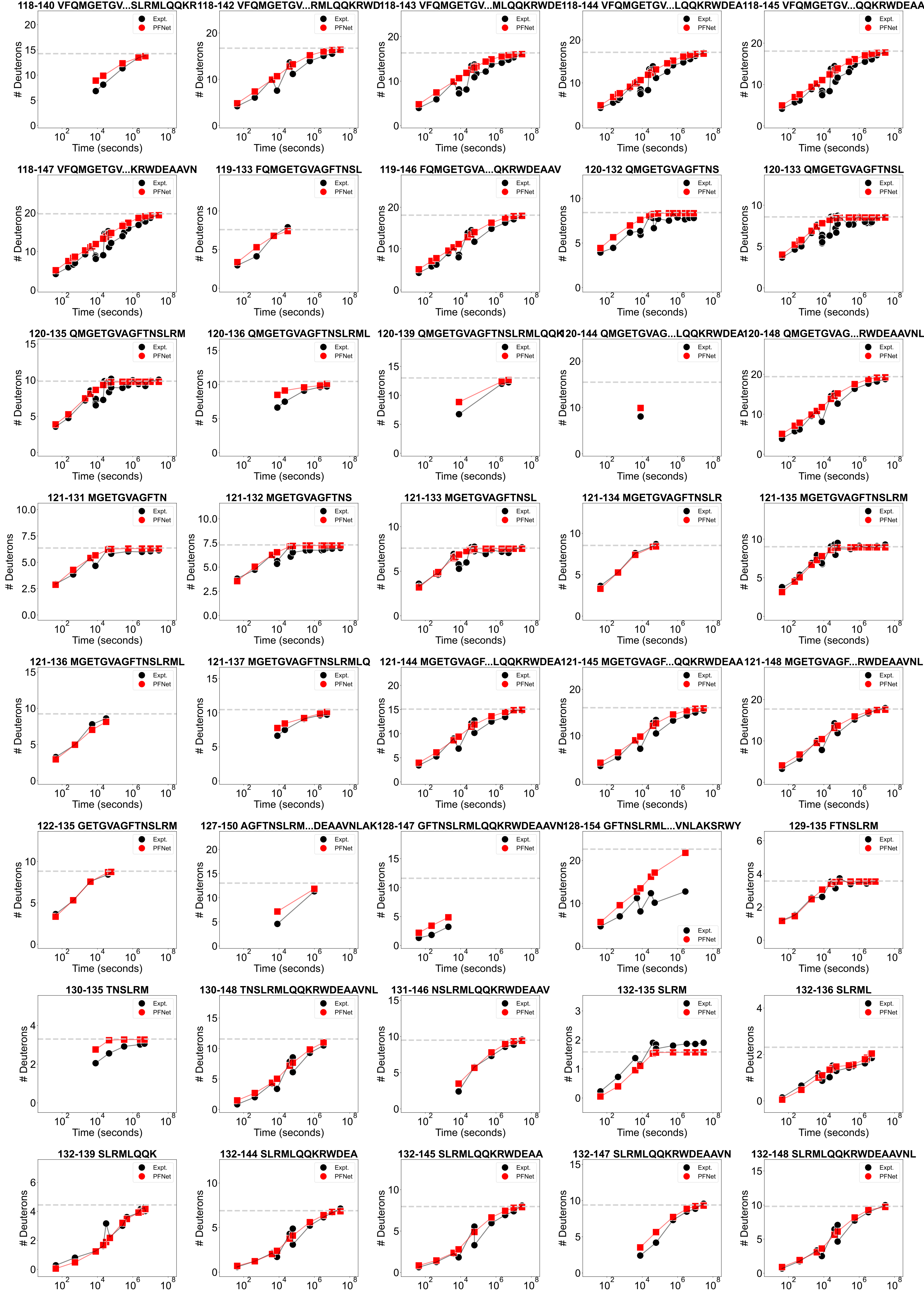
118-140 VFQMGETGV...SLRMLQQKR
118-142 VFQMGETGV...RMLQQKRWD
118-143 VFQMGETGV...MLQQKRWDE
118-144 VFQMGETGV...LQQKRWDEA
118-145 VFQMGETGV...QQKRWDEAA
118-147 VFQMGETGV...KRWDEAAVN
119-133 FQMGETGVAGFTNSL
119-146 FQMGETGVA...QKRWDEAAV
120-132 QMGETGVAGFTNS
120-133 QMGETGVAGFTNSL
120-135 QMGETGVAGFTNSLRM
120-136 QMGETGVAGFTNSLRML
120-139 QMGETGVAGFTNSLRMLQQK
120-144 QMGETGVAG...LQQKRWDEA
120-148 QMGETGVAG...RWDEAAVNL
121-131 MGETGVAGFTN
121-132 MGETGVAGFTNS
121-133 MGETGVAGFTNSL
121-134 MGETGVAGFTNSLR
121-135 MGETGVAGFTNSLRM
121-136 MGETGVAGFTNSLRML
121-137 MGETGVAGFTNSLRMLQ
121-144 MGETGVAGF...LQQKRWDEA
121-145 MGETGVAGF...QQKRWDEAA
121-148 MGETGVAGF...RWDEAAVNL
122-135 GETGVAGFTNSLRM
127-150 AGFTNSLRM...DEAAVNLAK
128-147 GFTNSLRMLQQKRWDEAAVN
128-154 GFTNSLRML...VNLAKSRWY
129-135 FTNSLRM
130-135 TNSLRM
130-148 TNSLRMLQQKRWDEAAVNL
131-146 NSLRMLQQKRWDEAAV
132-135 SLRM
132-136 SLRML
132-139 SLRMLQQK
132-144 SLRMLQQKRWDEA
132-145 SLRMLQQKRWDEAA
132-147 SLRMLQQKRWDEAAVN
132-148 SLRMLQQKRWDEAAVNL
Deuterons
Time (seconds)
Expt.
PFNet

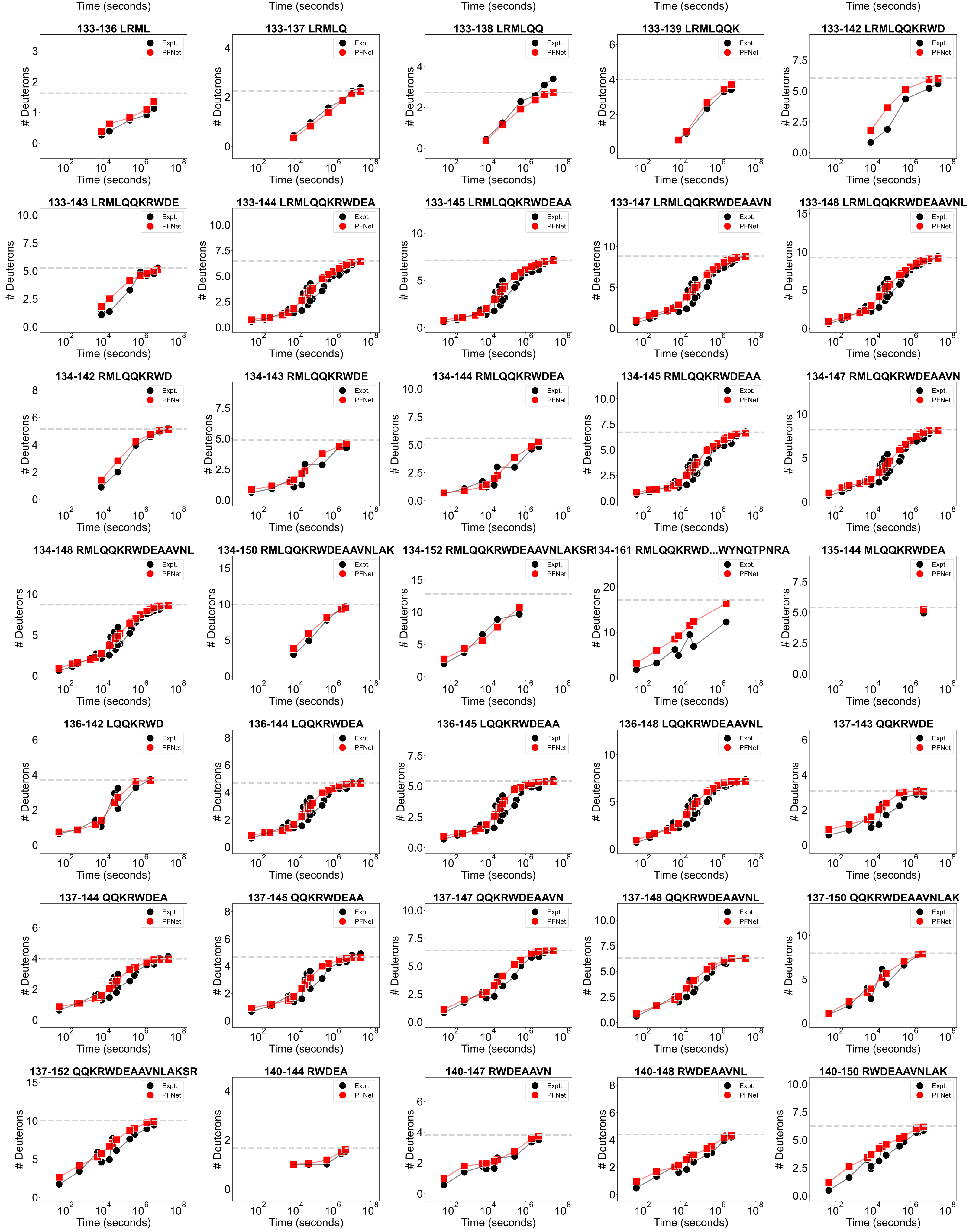

133-136 LRML
133-137 LRMLQ
133-138 LRMLQQ
133-139 LRMLQQK
133-142 LRMLQQKRWD
133-143 LRMLQQKRWDE
133-144 LRMLQQKRWDEA
133-145 LRMLQQKRWDEAA
133-147 LRMLQQKRWDEAAVN
133-148 LRMLQQKRWDEAAVNL
134-142 RMLQQKRWD
134-143 RMLQQKRWDE
134-144 RMLQQKRWDEA
134-145 RMLQQKRWDEAA
134-147 RMLQQKRWDEAAVN
134-148 RMLQQKRWDEAAVNL
134-150 RMLQQKRWDEAAVNLAK
134-152 RMLQQKRWDEAAVNLAKSR
134-161 RMLQQKRWD...WYNQTPNRA
135-144 MLQQKRWDEA
136-142 LQQKRWD
136-144 LQQKRWDEA
136-145 LQQKRWDEAA
136-148 LQQKRWDEAAVNL
137-143 QQKRWDE
137-144 QQKRWDEA
137-145 QQKRWDEAA
137-147 QQKRWDEAAVN
137-148 QQKRWDEAAVNL
137-150 QQKRWDEAAVNLAK
137-152 QQKRWDEAAVNLAKSR
140-144 RWDEA
140-147 RWDEAAVN
140-148 RWDEAAVNL
140-150 RWDEAAVNLAK
Expt.
PFNet
Deuterons
Time (seconds)

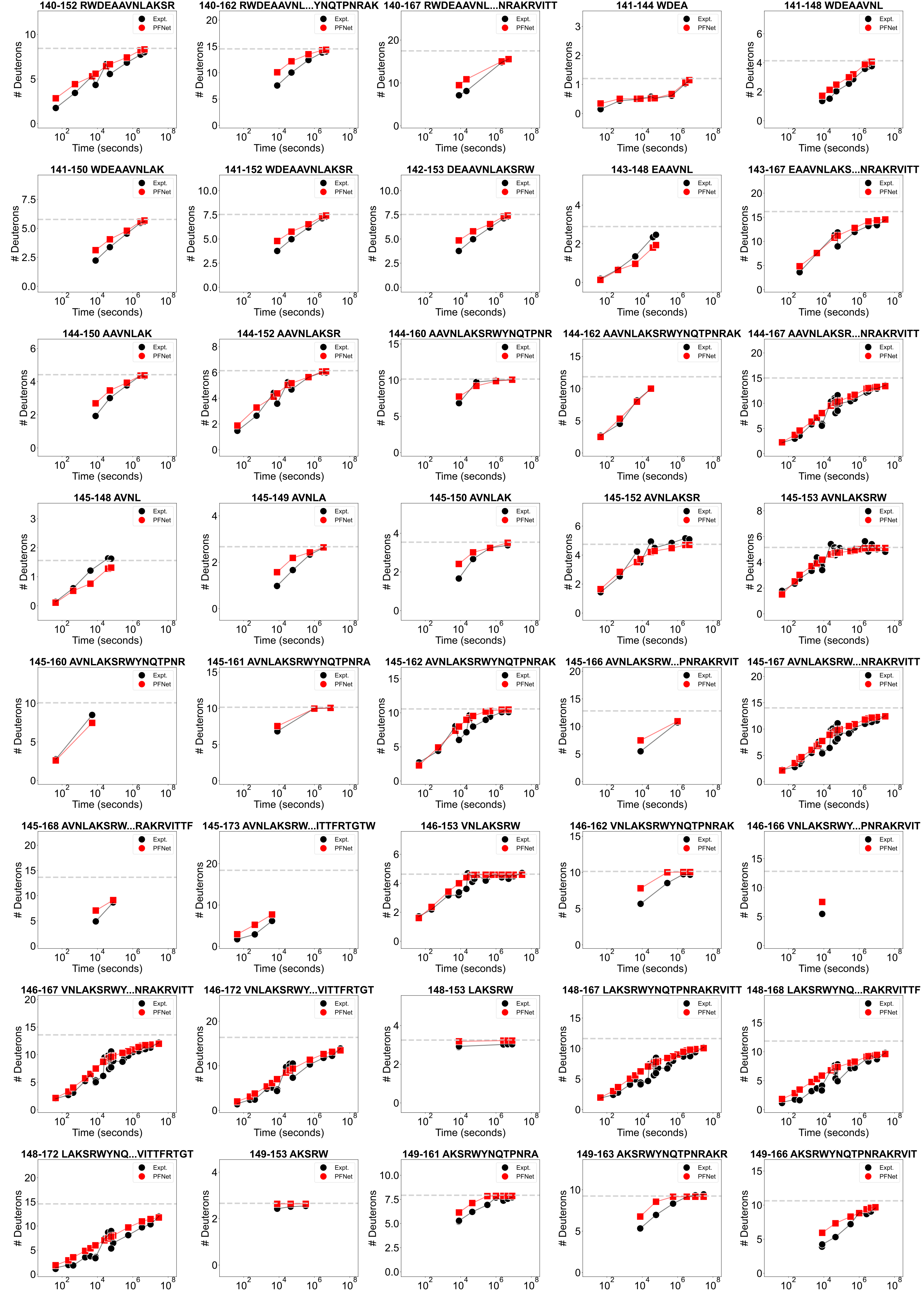
140-152 RWDEAAVNLAKSR
140-162 RWDEAAVNL...YNQTPNRAK
140-167 RWDEAAVNL...NRAKRVITT
141-144 WDEA
141-148 WDEAAVNL
141-150 WDEAAVNLAK
141-152 WDEAAVNLAKSR
142-153 DEAAVNLAKSRW
143-148 EAAVNL
143-167 EAAVNLAKS...NRAKRVITT
144-150 AAVNLAK
144-152 AAVNLAKSR
144-160 AAVNLAKSRWYNQTPNR
144-162 AAVNLAKSRWYNQTPNRAK
144-167 AAVNLAKSR...NRAKRVITT
145-148 AVNL
145-149 AVNLA
145-150 AVNLAK
145-152 AVNLAKSR
145-153 AVNLAKSRW
145-160 AVNLAKSRWYNQTPNR
145-161 AVNLAKSRWYNQTPNRA
145-162 AVNLAKSRWYNQTPNRAK
145-166 AVNLAKSRW...PNRAKRVIT
145-167 AVNLAKSRW...NRAKRVITT
145-168 AVNLAKSRW...RAKRVITTF
145-173 AVNLAKSRW...ITTFRTGTW
146-153 VNLAKSRW
146-162 VNLAKSRWYNQTPNRAK
146-166 VNLAKSRWY...PNRAKRVIT
146-167 VNLAKSRWY...NRAKRVITT
146-172 VNLAKSRWY...VITTFRTGT
148-153 LAKSRW
148-167 LAKSRWYNQTPNRAKRVITT
148-168 LAKSRWYNQ...RAKRVITTF
148-172 LAKSRWYNQ...VITTFRTGT
149-153 AKSRW
149-161 AKSRWYNQTPNRA
149-163 AKSRWYNQTPNRAKR
149-166 AKSRWYNQTPNRAKRVIT
Expt.
PFNet
Deuterons
Time (seconds)

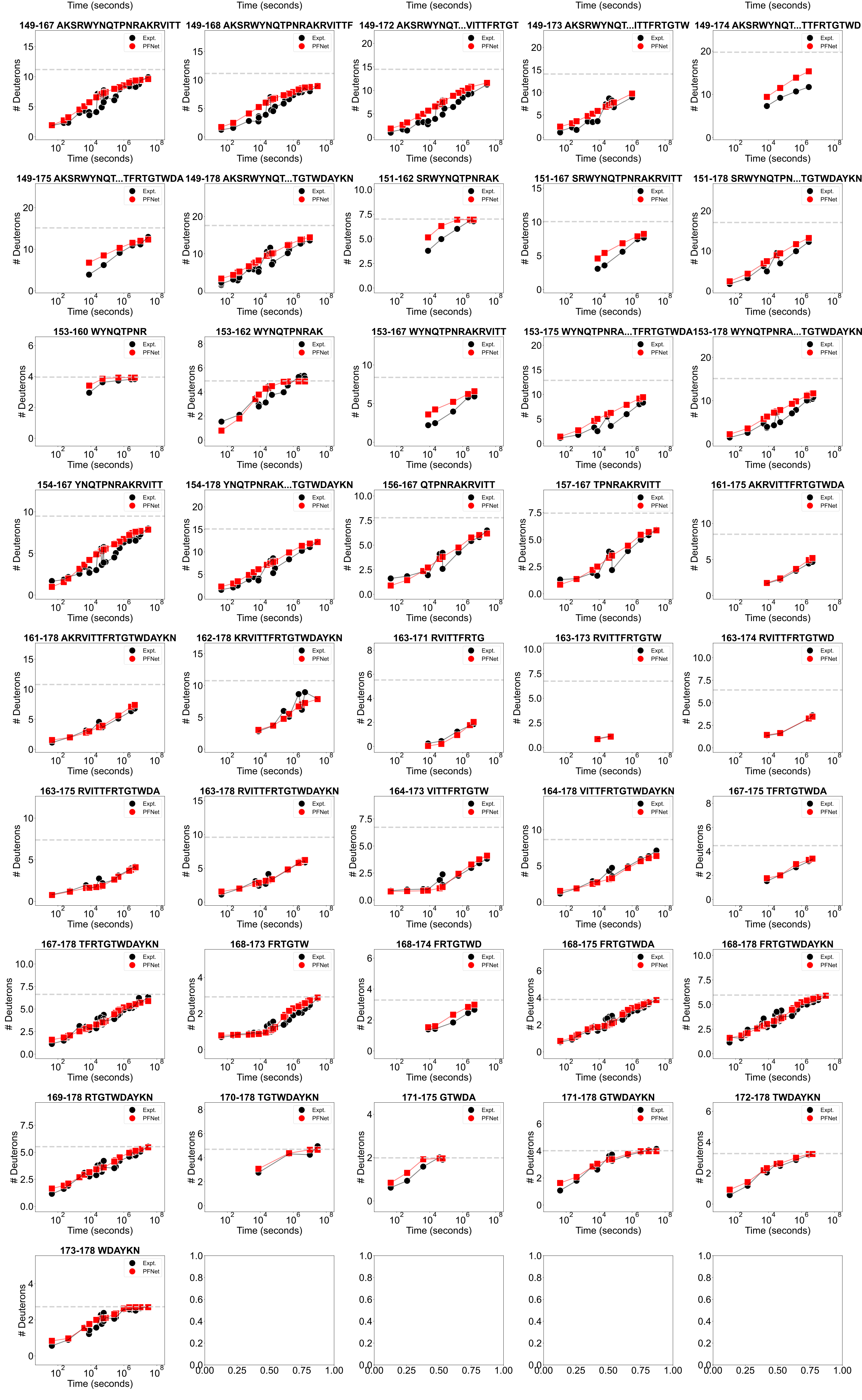
149-167 AKSRWYNQTPNRAKRVITT
149-168 AKSRWYNQTPNRAKRVITTF
149-172 AKSRWYNQT...VITTFRTGT
149-173 AKSRWYNQT...ITTFRTGTW
149-174 AKSRWYNQT...TTFRTGTWD
149-175 AKSRWYNQT...TFRTGTWDA
149-178 AKSRWYNQT...TGTWDAYKN
151-162 SRWYNQTPNRAK
151-167 SRWYNQTPNRAKRVITT
151-178 SRWYNQTPN...TGTWDAYKN
153-160 WYNQTPNR
153-162 WYNQTPNRAK
153-167 WYNQTPNRAKRVITT
153-175 WYNQTPNRA...TFRTGTWDA
153-178 WYNQTPNRA...TGTWDAYKN
154-167 YNQTPNRAKRVITT
154-178 YNQTPNRAK...TGTWDAYKN
156-167 QTPNRAKRVITT
157-167 TPNRAKRVITT
161-175 AKRVITTFRTGTWDA
161-178 AKRVITTFRTGTWDAYKN
162-178 KRVITTFRTGTWDAYKN
163-171 RVITTFRTG
163-173 RVITTFRTGTW
163-174 RVITTFRTGTWD
163-175 RVITTFRTGTWDA
163-178 RVITTFRTGTWDAYKN
164-173 VITTFRTGTW
164-178 VITTFRTGTWDAYKN
167-175 TFRTGTWDA
167-178 TFRTGTWDAYKN
168-173 FRTGTW
168-174 FRTGTWD
168-175 FRTGTWDA
168-178 FRTGTWDAYKN
169-178 RTGTWDAYKN
170-178 TGTWDAYKN
171-175 GTWDA
171-178 GTWDAYKN
172-178 TWDAYKN
173-178 WDAYKN
Expt.
PFNet
Deuterons
Time (seconds)
