# Supplementary material for: A machine learning method for calculating highly localized protein stabilities
Source: Nat Commun. 2026 Sep 1;17:10414. doi: 10.1038/s41467-026-75590-9 (PMC13631364; doi:10.1038/s41467-026-75590-9)
Supplement: Supplementary file 6 [file 41467_2026_75590_MOESM4_ESM.zip › Supplementary Data 2/PFNet_uptake_ecDHFR_TMP_0.pdf]

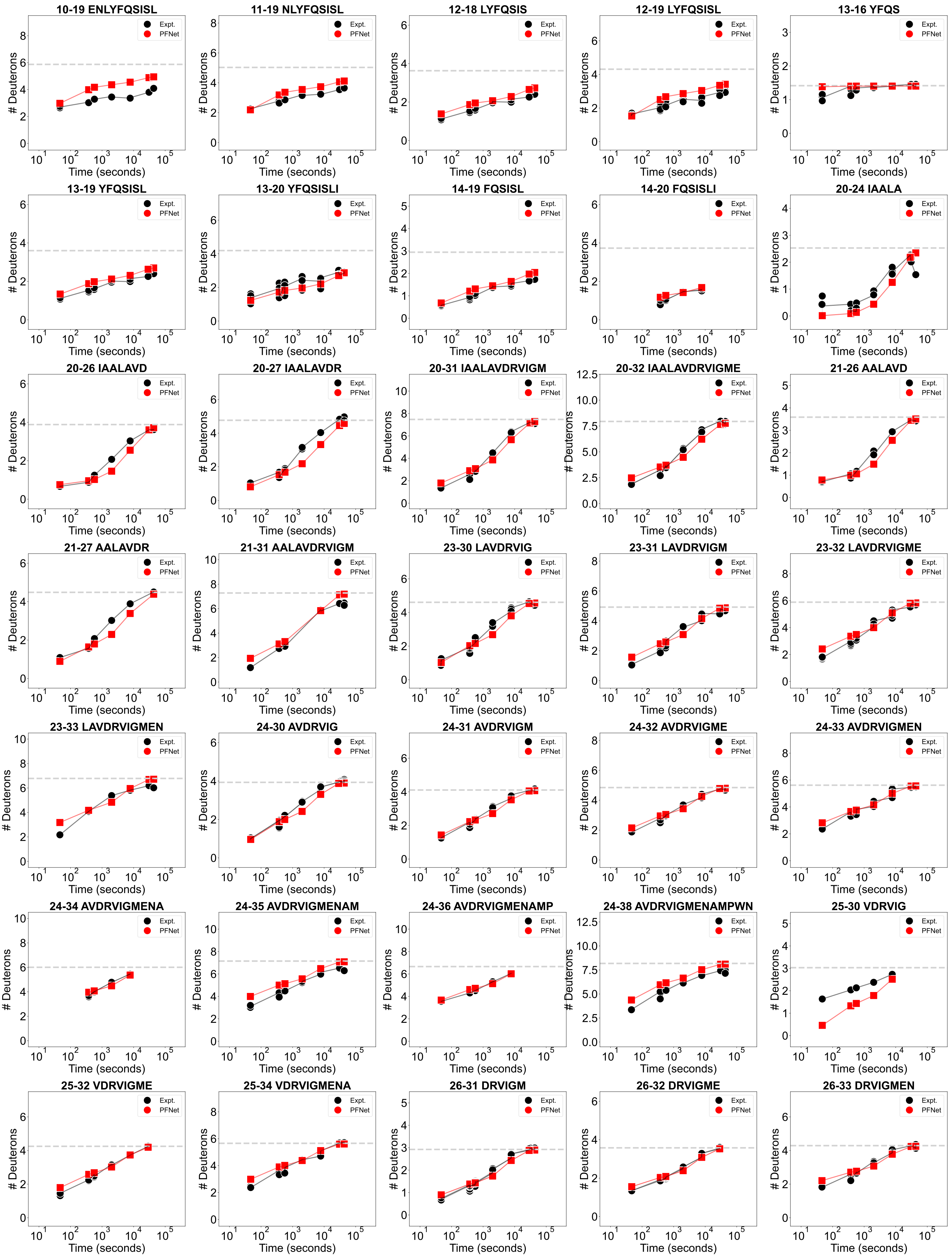
10-19 ENLYFQSISL
11-19 NLYFQSISL
12-18 LYFQSIS
12-19 LYFQSISL
13-16 YFQS
13-19 YFQSISL
13-20 YFQSISLI
14-19 FQSISL
14-20 FQSISLI
20-24 IAALA
20-26 IAALAVD
20-27 IAALAVDR
20-31 IAALAVDRVIGM
20-32 IAALAVDRVIGME
21-26 AALAVD
21-27 AALAVDR
21-31 AALAVDRVIGM
23-30 LAVDRVIG
23-31 LAVDRVIGM
23-32 LAVDRVIGME
23-33 LAVDRVIGMEN
24-30 AVDRVIG
24-31 AVDRVIGM
24-32 AVDRVIGME
24-33 AVDRVIGMEN
24-34 AVDRVIGMENA
24-35 AVDRVIGMENAM
24-36 AVDRVIGMENAMP
24-38 AVDRVIGMENAMPWN
25-30 VDRVIG
25-32 VDRVIGME
25-34 VDRVIGMENA
26-31 DRVIGM
26-32 DRVIGME
26-33 DRVIGMEN
Expt.
PFNet
Deuterons
Time (seconds)

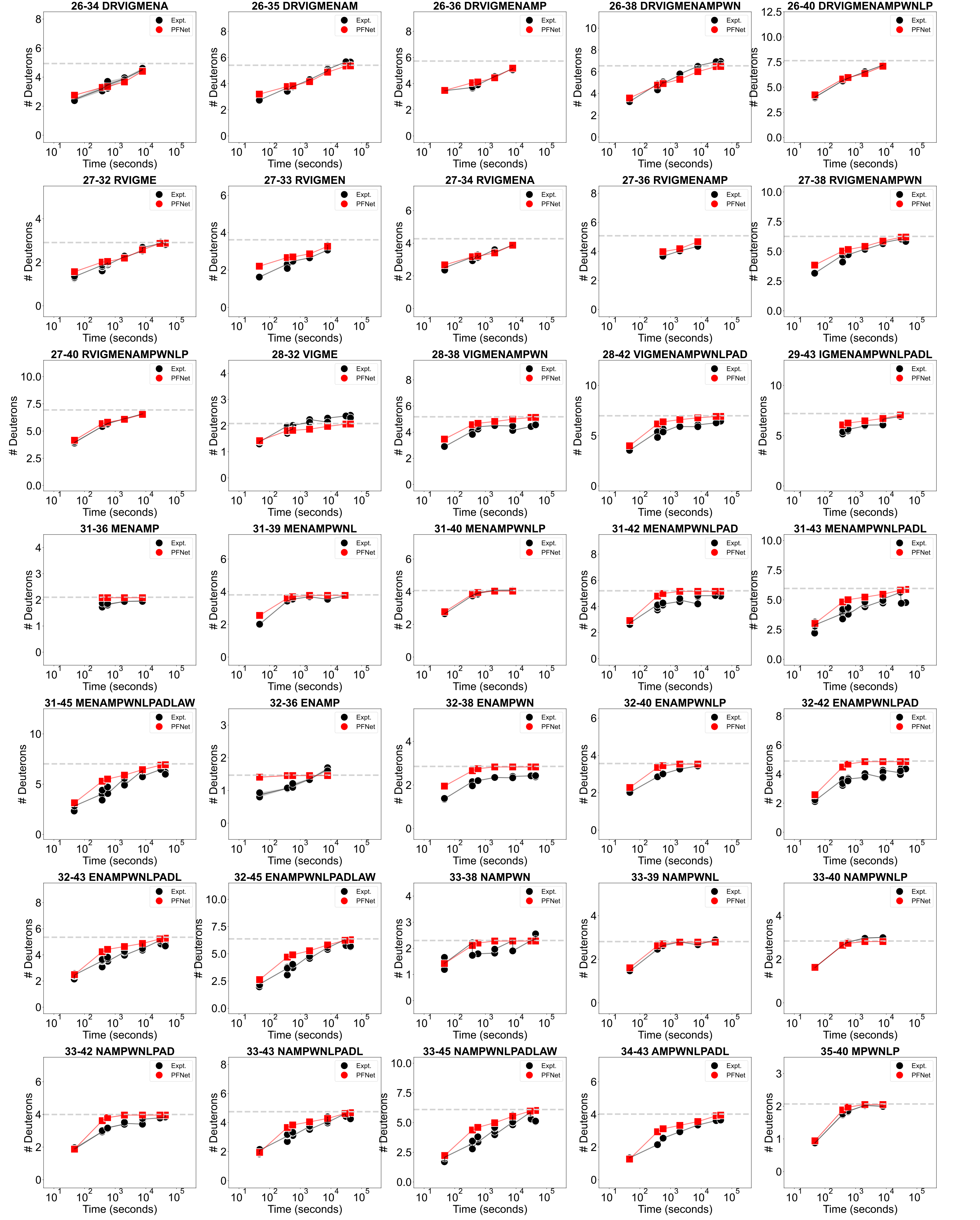
26-34 DRVIGMENA
26-35 DRVIGMENAM
26-36 DRVIGMENAMP
26-38 DRVIGMENAMPWN
26-40 DRVIGMENAMPWNLP
27-32 RVIGME
27-33 RVIGMEN
27-34 RVIGMENA
27-36 RVIGMENAMP
27-38 RVIGMENAMPWN
27-40 RVIGMENAMPWNLP
28-32 VIGME
28-38 VIGMENAMPWN
28-42 VIGMENAMPWNLPAD
29-43 IGMENAMPWNLPADL
31-36 MENAMP
31-39 MENAMPWNL
31-40 MENAMPWNLP
31-42 MENAMPWNLPAD
31-43 MENAMPWNLPADL
31-45 MENAMPWNLPADLAW
32-36 ENAMP
32-38 ENAMPWN
32-40 ENAMPWNLP
32-42 ENAMPWNLPAD
32-43 ENAMPWNLPADL
32-45 ENAMPWNLPADLAW
33-38 NAMPWN
33-39 NAMPWNL
33-40 NAMPWNLP
33-42 NAMPWNLPAD
33-43 NAMPWNLPADL
33-45 NAMPWNLPADLAW
34-43 AMPWNLPADL
35-40 MPWNLP
Expt.
PFNet
Deuterons
Time (seconds)

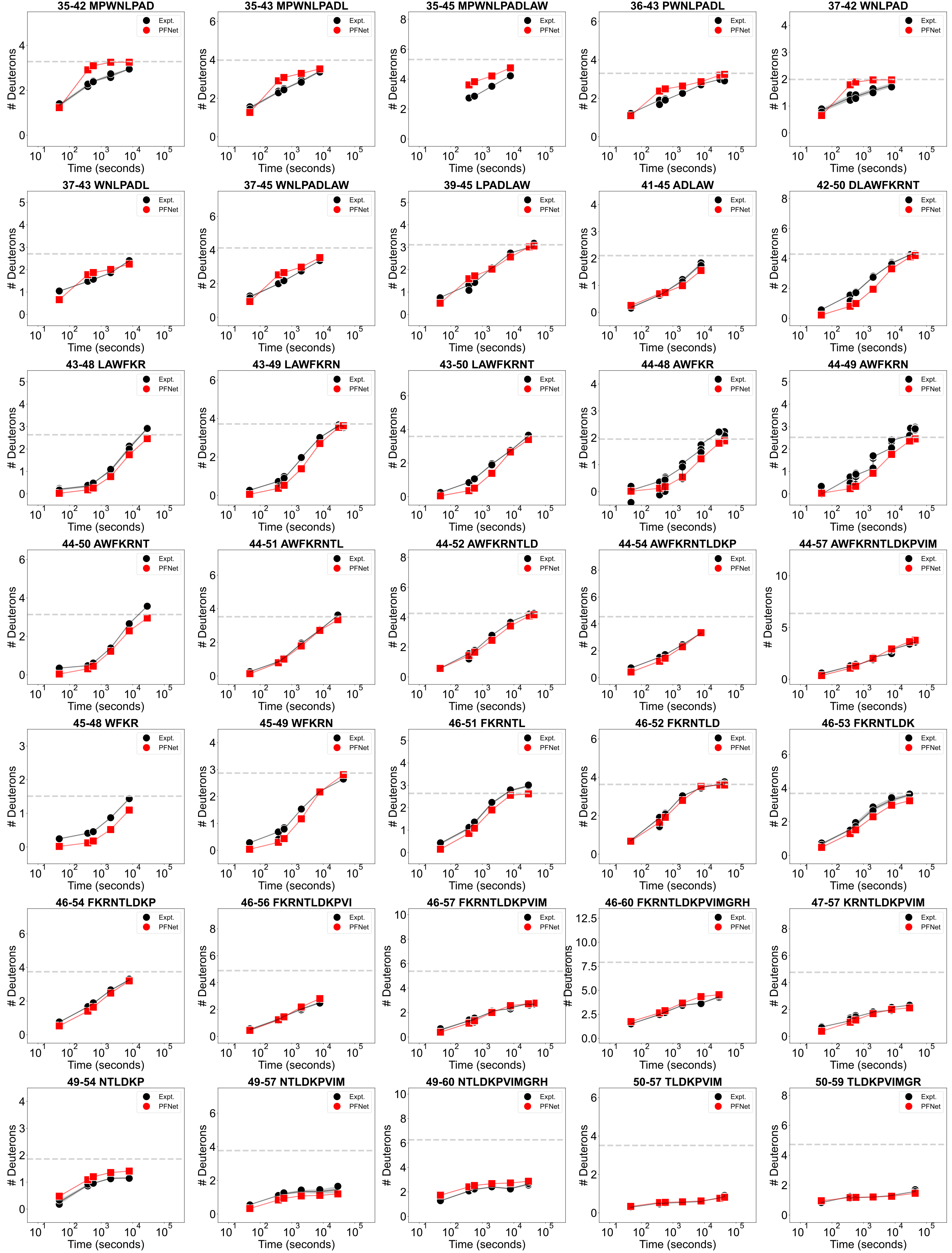
35-42 MPWNLPAD
35-43 MPWNLPADL
35-45 MPWNLPADLAW
36-43 PWNLPADL
37-42 WNLPAD
37-43 WNLPADL
37-45 WNLPADLAW
39-45 LPADLAW
41-45 ADLAW
42-50 DLAWFKRNT
43-48 LAWFKR
43-49 LAWFKRN
43-50 LAWFKRNT
44-48 AWFKR
44-49 AWFKRN
44-50 AWFKRNT
44-51 AWFKRNTL
44-52 AWFKRNTLD
44-54 AWFKRNTLDKP
44-57 AWFKRNTLDKPVIM
45-48 WFKR
45-49 WFKRN
46-51 FKRNTL
46-52 FKRNTLD
46-53 FKRNTLDK
46-54 FKRNTLDKP
46-56 FKRNTLDKPVI
46-57 FKRNTLDKPVIM
46-60 FKRNTLDKPVIMGRH
47-57 KRNTLDKPVIM
49-54 NTLDKP
49-57 NTLDKPVIM
49-60 NTLDKPVIMGRH
50-57 TLDKPVIM
50-59 TLDKPVIMGR
Expt.
PFNet
Deuterons
Time (seconds)

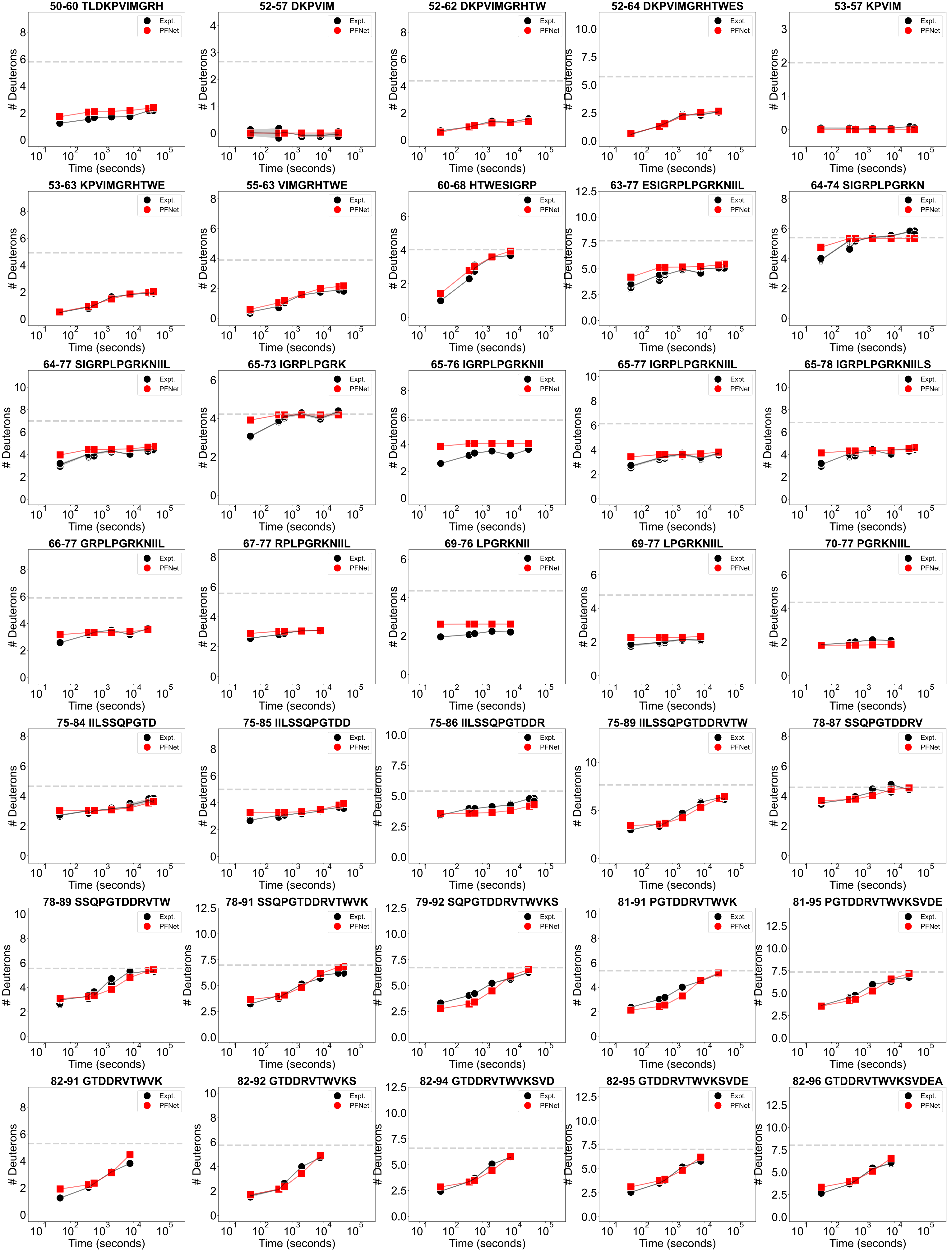
50-60 TLDKPVIMGRH
52-57 DKPVIM
52-62 DKPVIMGRHTW
52-64 DKPVIMGRHTWES
53-57 KPVIM
53-63 KPVIMGRHTWE
55-63 VIMGRHTWE
60-68 HTWESIGRP
63-77 ESIGRPLPGRKNIIL
64-74 SIGRPLPGRKN
64-77 SIGRPLPGRKNIIL
65-73 IGRPLPGRK
65-76 IGRPLPGRKNII
65-77 IGRPLPGRKNIIL
65-78 IGRPLPGRKNIILS
66-77 GRPLPGRKNIIL
67-77 RPLPGRKNIIL
69-76 LPGRKNII
69-77 LPGRKNIIL
70-77 PGRKNIIL
75-84 IILSSQPGTD
75-85 IILSSQPGTDD
75-86 IILSSQPGTDDR
75-89 IILSSQPGTDDRVTW
78-87 SSQPGTDDRV
78-89 SSQPGTDDRVTW
78-91 SSQPGTDDRVTWVK
79-92 SQPGTDDRVTWVKS
81-91 PGTDDRVTWVK
81-95 PGTDDRVTWVKSVDE
82-91 GTDDRVTWVK
82-92 GTDDRVTWVKS
82-94 GTDDRVTWVKSVD
82-95 GTDDRVTWVKSVDE
82-96 GTDDRVTWVKSVDEA
Expt.
PFNet
Deuterons
Time (seconds)

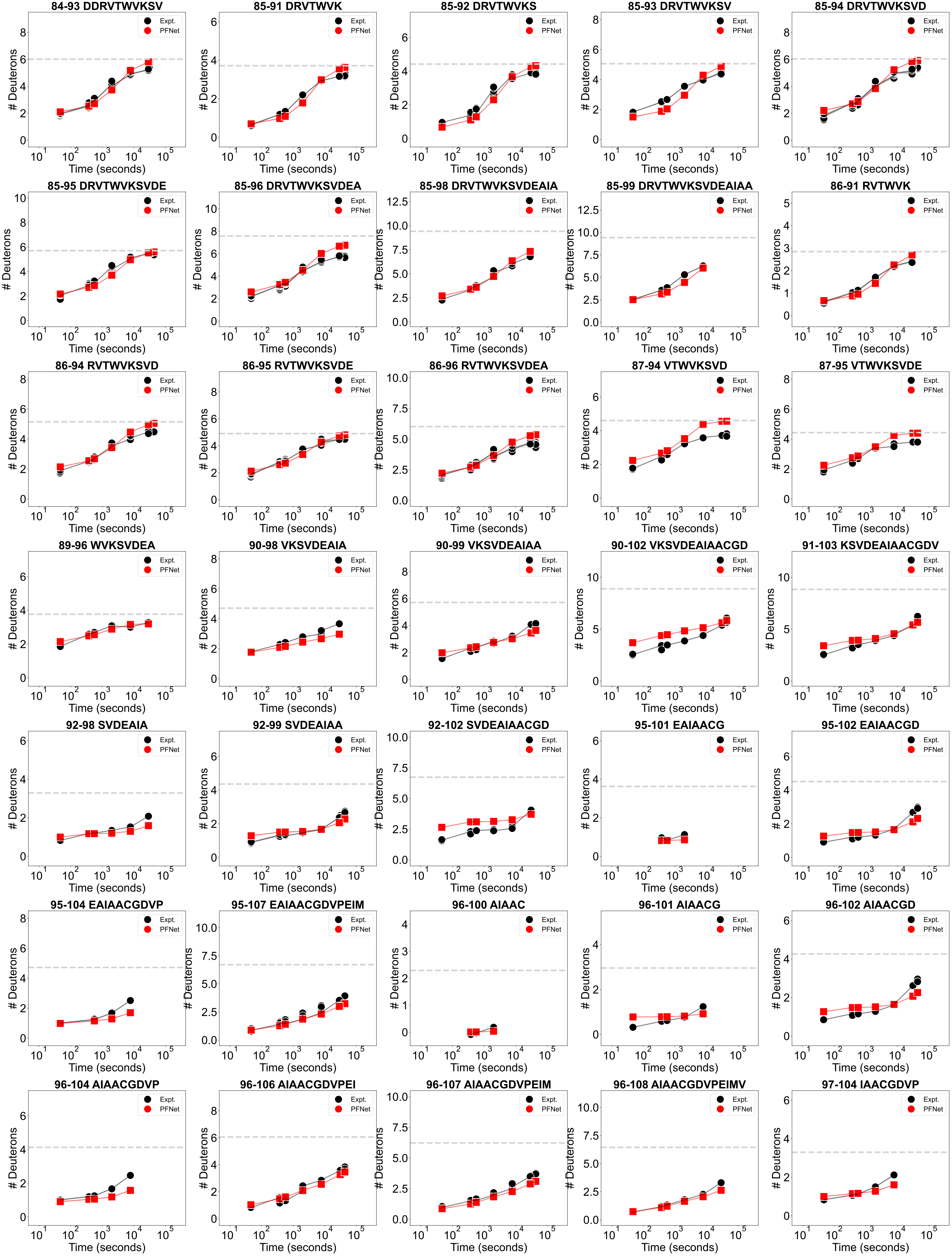
84-93 DDRVTWVKSV
85-91 DRVTWVK
85-92 DRVTWVKS
85-93 DRVTWVKSV
85-94 DRVTWVKSVD
85-95 DRVTWVKSVDE
85-96 DRVTWVKSVDEA
85-98 DRVTWVKSVDEAIA
85-99 DRVTWVKSVDEAIAA
86-91 RVTWVK
86-94 RVTWVKSVD
86-95 RVTWVKSVDE
86-96 RVTWVKSVDEA
87-94 VTWVKSVD
87-95 VTWVKSVDE
89-96 WVKSVDEA
90-98 VKSVDEAIA
90-99 VKSVDEAIAA
90-102 VKSVDEAIAACGD
91-103 KSVDEAIAACGDV
92-98 SVDEAIA
92-99 SVDEAIAA
92-102 SVDEAIAACGD
95-101 EAIAACG
95-102 EAIAACGD
95-104 EAIAACGDVP
95-107 EAIAACGDVPEIM
96-100 AIAAC
96-101 AIAACG
96-102 AIAACGD
96-104 AIAACGDVP
96-106 AIAACGDVPEI
96-107 AIAACGDVPEIM
96-108 AIAACGDVPEIMV
97-104 IAACGDVP
Expt.
PFNet
Deuterons
Time (seconds)

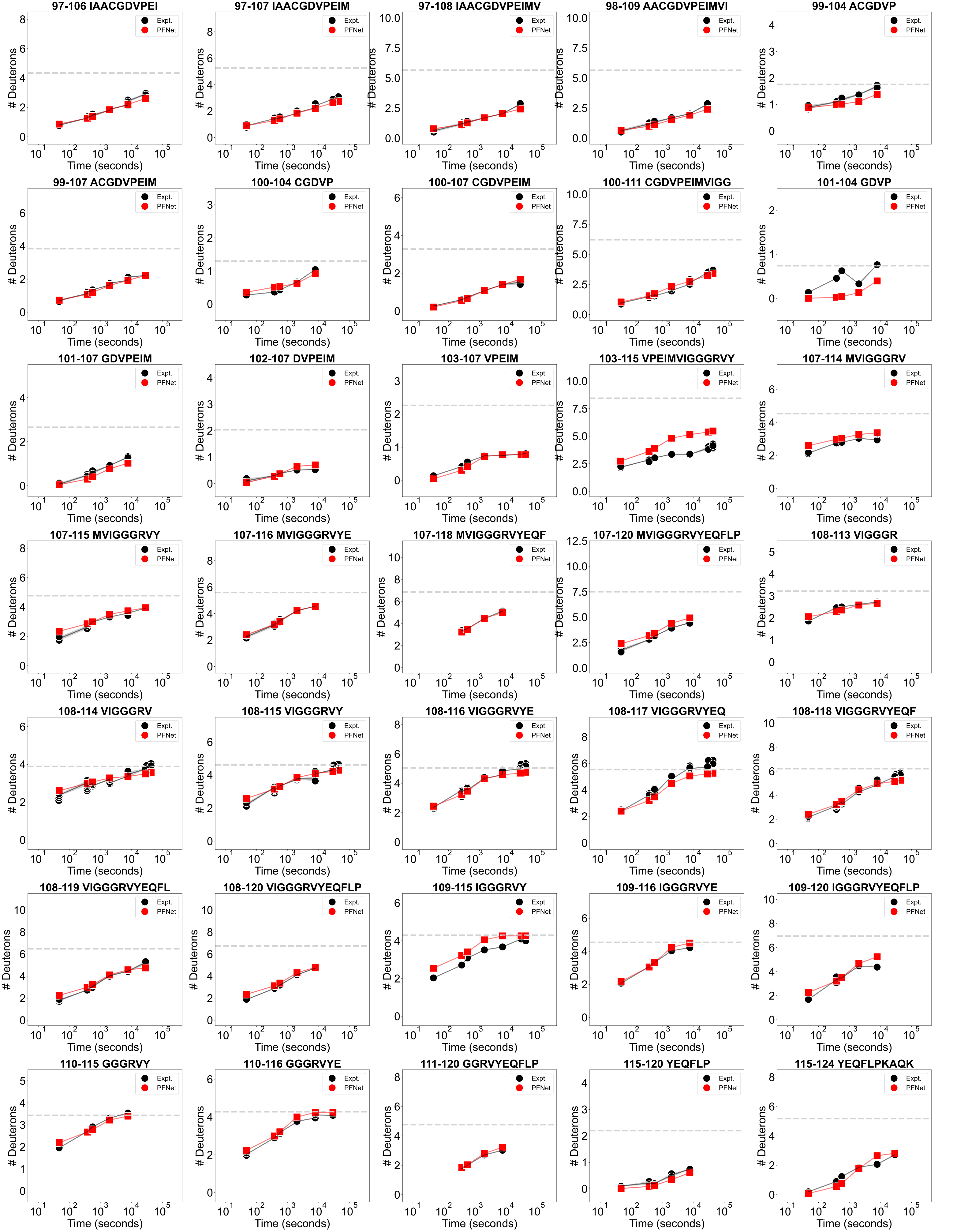
97-106 IAACGDVPEI
97-107 IAACGDVPEIM
97-108 IAACGDVPEIMV
98-109 AACGDVPEIMVI
99-104 ACGDVP
99-107 ACGDVPEIM
100-104 CGDVP
100-107 CGDVPEIM
100-111 CGDVPEIMVIGG
101-104 GDVP
101-107 GDVPEIM
102-107 DVPEIM
103-107 VPEIM
103-115 VPEIMVIGGGRVY
107-114 MVIGGGRV
107-115 MVIGGGRVY
107-116 MVIGGGRVYE
107-118 MVIGGGRVYEQF
107-120 MVIGGGRVYEQFLP
108-113 VIGGGR
108-114 VIGGGRV
108-115 VIGGGRVY
108-116 VIGGGRVYE
108-117 VIGGGRVYEQ
108-118 VIGGGRVYEQF
108-119 VIGGGRVYEQFL
108-120 VIGGGRVYEQFLP
109-115 IGGGRVY
109-116 IGGGRVYE
109-120 IGGGRVYEQFLP
110-115 GGGRVY
110-116 GGGRVYE
111-120 GGRVYEQFLP
115-120 YEQFLP
115-124 YEQFLPKAQK
Expt.
PFNet
Deuterons
Time (seconds)

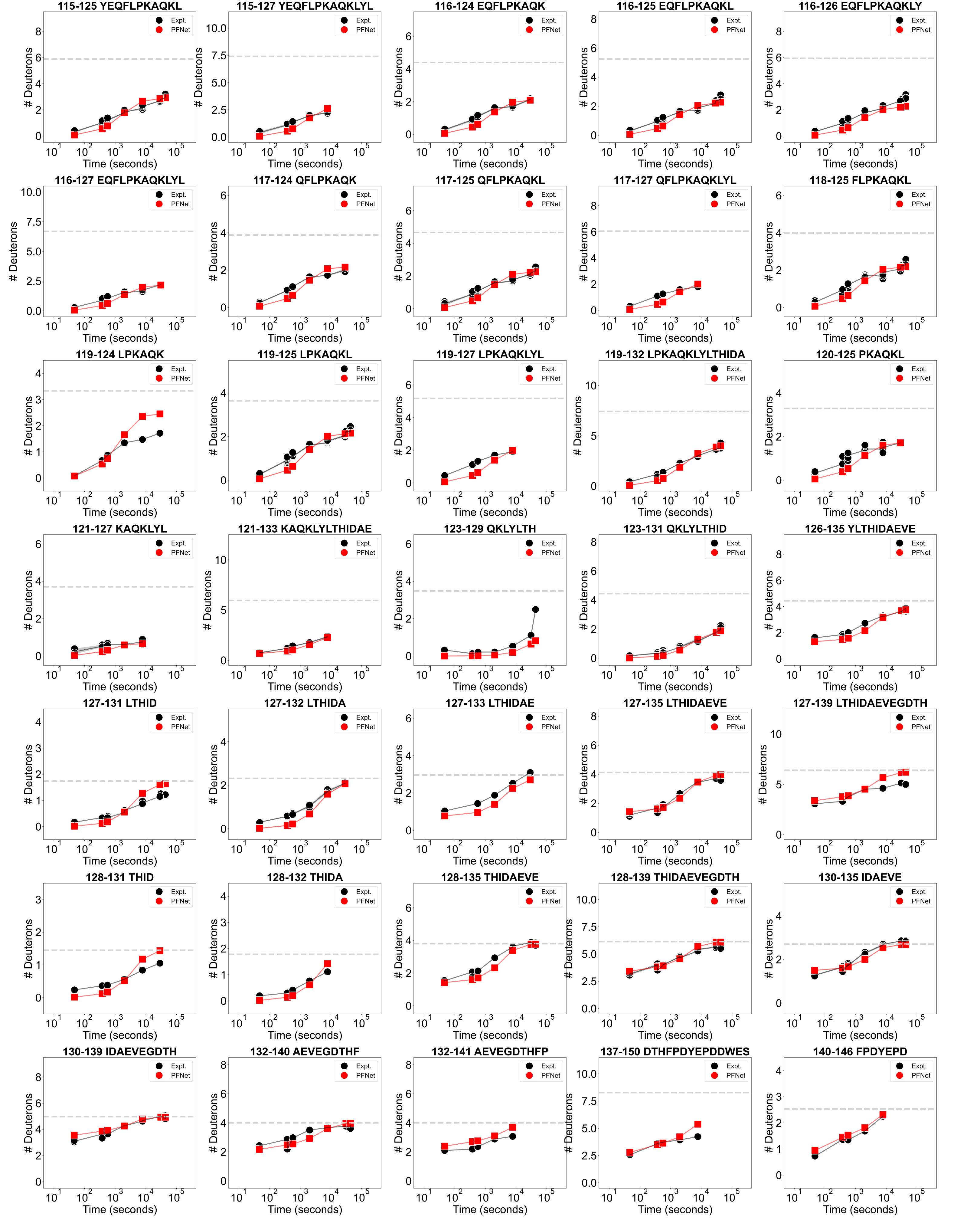
115-125 YEQFLPKAQKL
115-127 YEQFLPKAQKLYL
116-124 EQFLPKAQK
116-125 EQFLPKAQKL
116-126 EQFLPKAQKLY
116-127 EQFLPKAQKLYL
117-124 QFLPKAQK
117-125 QFLPKAQKL
117-127 QFLPKAQKLYL
118-125 FLPKAQKL
119-124 LPKAQK
119-125 LPKAQKL
119-127 LPKAQKLYL
119-132 LPKAQKLYLTHIDA
120-125 PKAQKL
121-127 KAQKLYL
121-133 KAQKLYLTHIDAE
123-129 QKLYLTH
123-131 QKLYLTHID
126-135 YLTHIDAEVE
127-131 LTHID
127-132 LTHIDA
127-133 LTHIDAE
127-135 LTHIDAEVE
127-139 LTHIDAEVEGDTH
128-131 THID
128-132 THIDA
128-135 THIDAEVE
128-139 THIDAEVEGDTH
130-135 IDAEVE
130-139 IDAEVEGDTH
132-140 AEVEGDTHF
132-141 AEVEGDTHFP
137-150 DTHFPDYEPDDWES
140-146 FPDYEPD
Expt.
PFNet
Deuterons
Time (seconds)

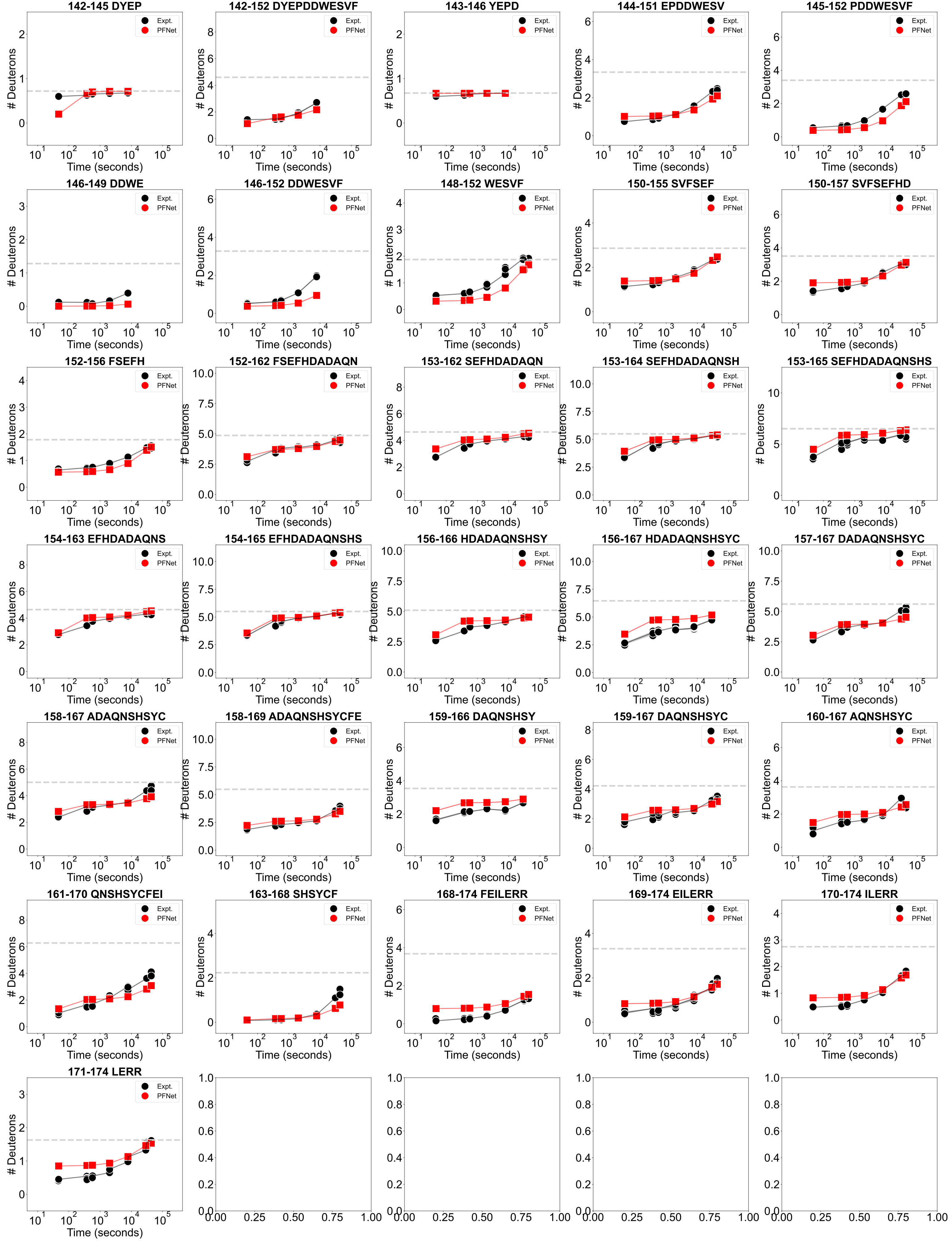
142-145 DYEP
142-152 DYEPDDWESVF
143-146 YEPD
144-151 EPDDWESV
145-152 PDDWESVF
146-149 DDWE
146-152 DDWESVF
148-152 WESVF
150-155 SVFSEF
150-157 SVFSEFHD
152-156 FSEFH
152-162 FSEFHDADAQN
153-162 SEFHDADAQN
153-164 SEFHDADAQNSH
153-165 SEFHDADAQNSHS
154-163 EFHDADAQNS
154-165 EFHDADAQNSHS
156-166 HDADAQNSHSY
156-167 HDADAQNSHSYC
157-167 DADAQNSHSYC
158-167 ADAQNSHSYC
158-169 ADAQNSHSYCFE
159-166 DAQNSHSY
159-167 DAQNSHSYC
160-167 AQNSHSYC
161-170 QNSHSYCFEI
163-168 SHSYCF
168-174 FEILERR
169-174 EILERR
170-174 ILERR
171-174 LERR
Expt.
PFNet
Deuterons
Time (seconds)
