# Supplementary material for: A machine learning method for calculating highly localized protein stabilities
Source: Nat Commun. 2026 Sep 1;17:10414. doi: 10.1038/s41467-026-75590-9 (PMC13631364; doi:10.1038/s41467-026-75590-9)
Supplement: Supplementary file 6 [file 41467_2026_75590_MOESM4_ESM.zip › Supplementary Data 2/PFNet_uptake_spike_protein_2P_RBD_APO_0.pdf]

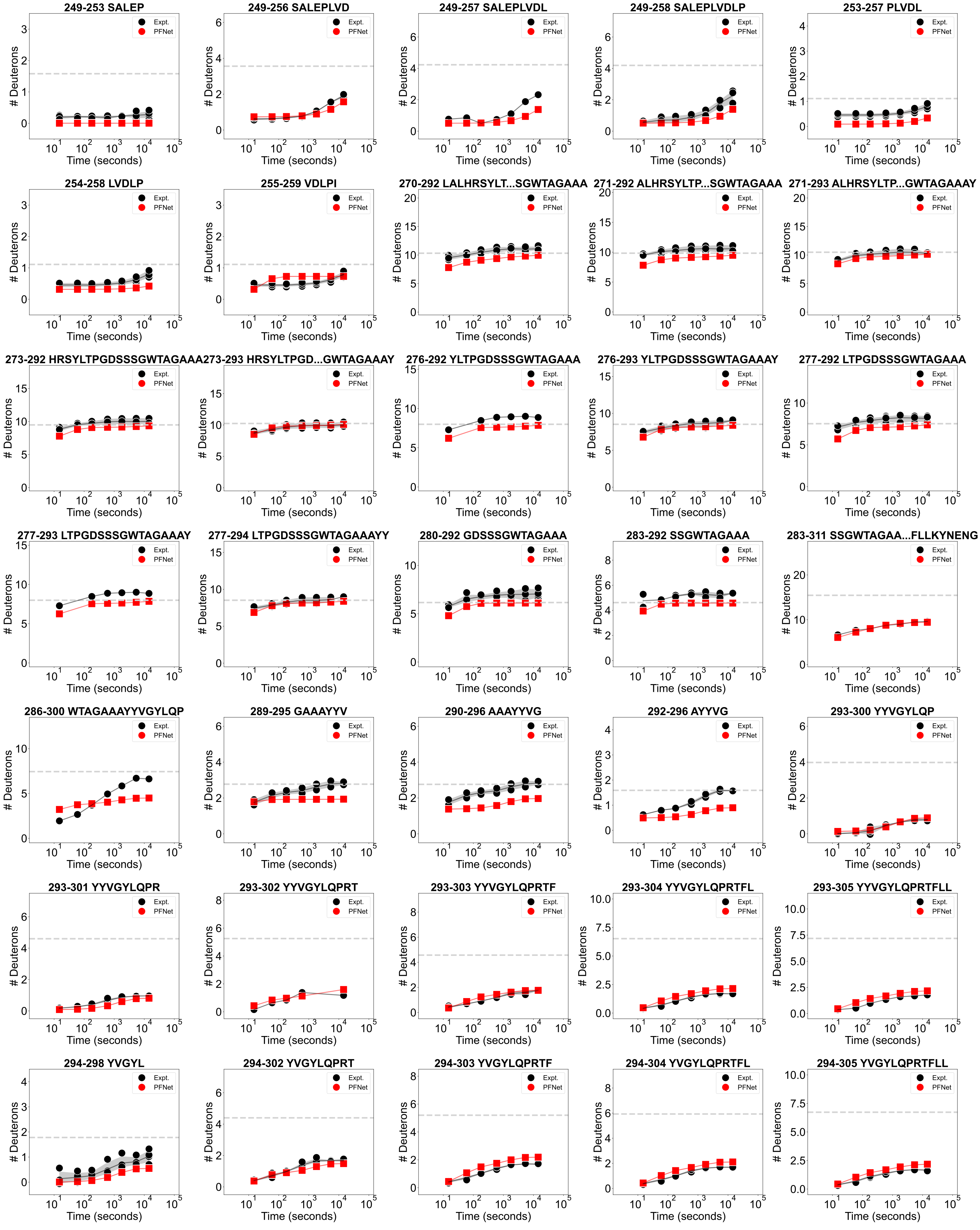

249-253 SALEP
249-256 SALEPLVD
249-257 SALEPLVDL
249-258 SALEPLVDLP
253-257 PLVDL
254-258 LVDLP
255-259 VDLPI
270-292 LALHRSYLT...SGWTAGAAA
271-292 ALHRSYLTP...SGWTAGAAA
271-293 ALHRSYLTP...GWTAGAAAY
273-292 HRSYLTPGDSSSGWTAGAAA
273-293 HRSYLTPGD...GWTAGAAAY
276-292 YLTPGDSSSGWTAGAAA
276-293 YLTPGDSSSGWTAGAAAY
277-292 LTPGDSSSGWTAGAAA
277-293 LTPGDSSSGWTAGAAAY
277-294 LTPGDSSSGWTAGAAAYY
280-292 GDSSSGWTAGAAA
283-292 SSGWTAGAAA
283-311 SSGWTAGAA...FLLKYNENG
286-300 WTAGAAAYYVGYLQP
289-295 GAAAYYV
290-296 AAAYYVG
292-296 AYYVG
293-300 YYVGYLQP
293-301 YYVGYLQPR
293-302 YYVGYLQPRT
293-303 YYVGYLQPRTF
293-304 YYVGYLQPRTFL
293-305 YYVGYLQPRTFLL
294-298 YVGYL
294-302 YVGYLQPRT
294-303 YVGYLQPRTF
294-304 YVGYLQPRTFL
294-305 YVGYLQPRTFLL
Expt.
PFNet
Deuterons
Time (seconds)

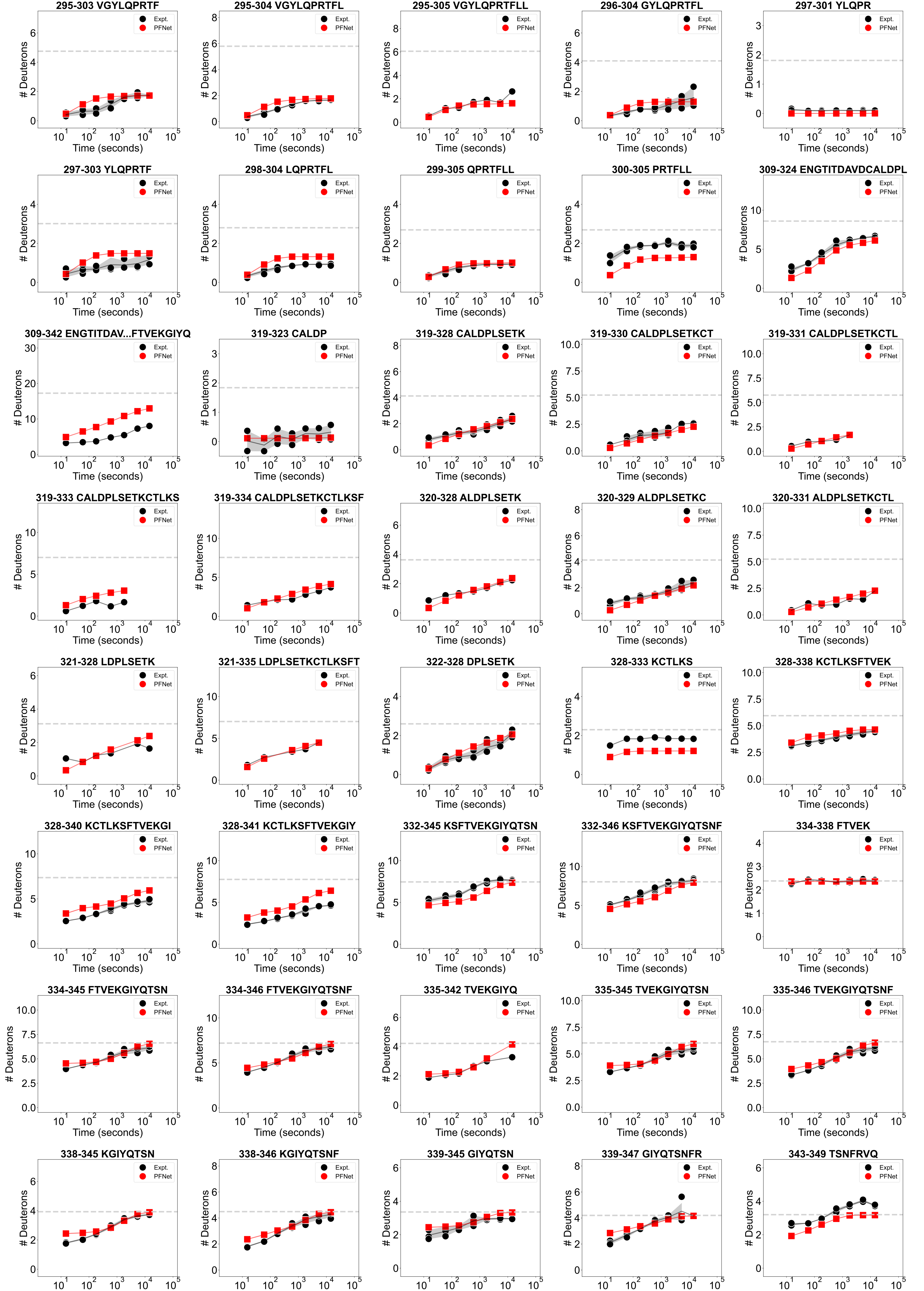
295-303 VGYLQPRTF
295-304 VGYLQPRTFL
295-305 VGYLQPRTFLL
296-304 GYLQPRTFL
297-301 YLQPR
297-303 YLQPRTF
298-304 LQPRTFL
299-305 QPRTFLL
300-305 PRTFLL
309-324 ENGTITDAVDCALDPL
309-342 ENGTITDAV...FTVEKGIYQ
319-323 CALDP
319-328 CALDPLSETK
319-330 CALDPLSETKCT
319-331 CALDPLSETKCTL
319-333 CALDPLSETKCTLKS
319-334 CALDPLSETKCTLKSF
320-328 ALDPLSETK
320-329 ALDPLSETKC
320-331 ALDPLSETKCTL
321-328 LDPLSETK
321-335 LDPLSETKCTLKSFT
322-328 DPLSETK
328-333 KCTLKS
328-338 KCTLKSFTVEK
328-340 KCTLKSFTVEKGI
328-341 KCTLKSFTVEKGIY
332-345 KSFTVEKGIYQTSN
332-346 KSFTVEKGIYQTSNF
334-338 FTVEK
334-345 FTVEKGIYQTSN
334-346 FTVEKGIYQTSNF
335-342 TVEKGIYQ
335-345 TVEKGIYQTSN
335-346 TVEKGIYQTSNF
338-345 KGIYQTSN
338-346 KGIYQTSNF
339-345 GIYQTSN
339-347 GIYQTSNFR
343-349 TSNFRVQ
Expt.
PFNet
Deuterons
Time (seconds)

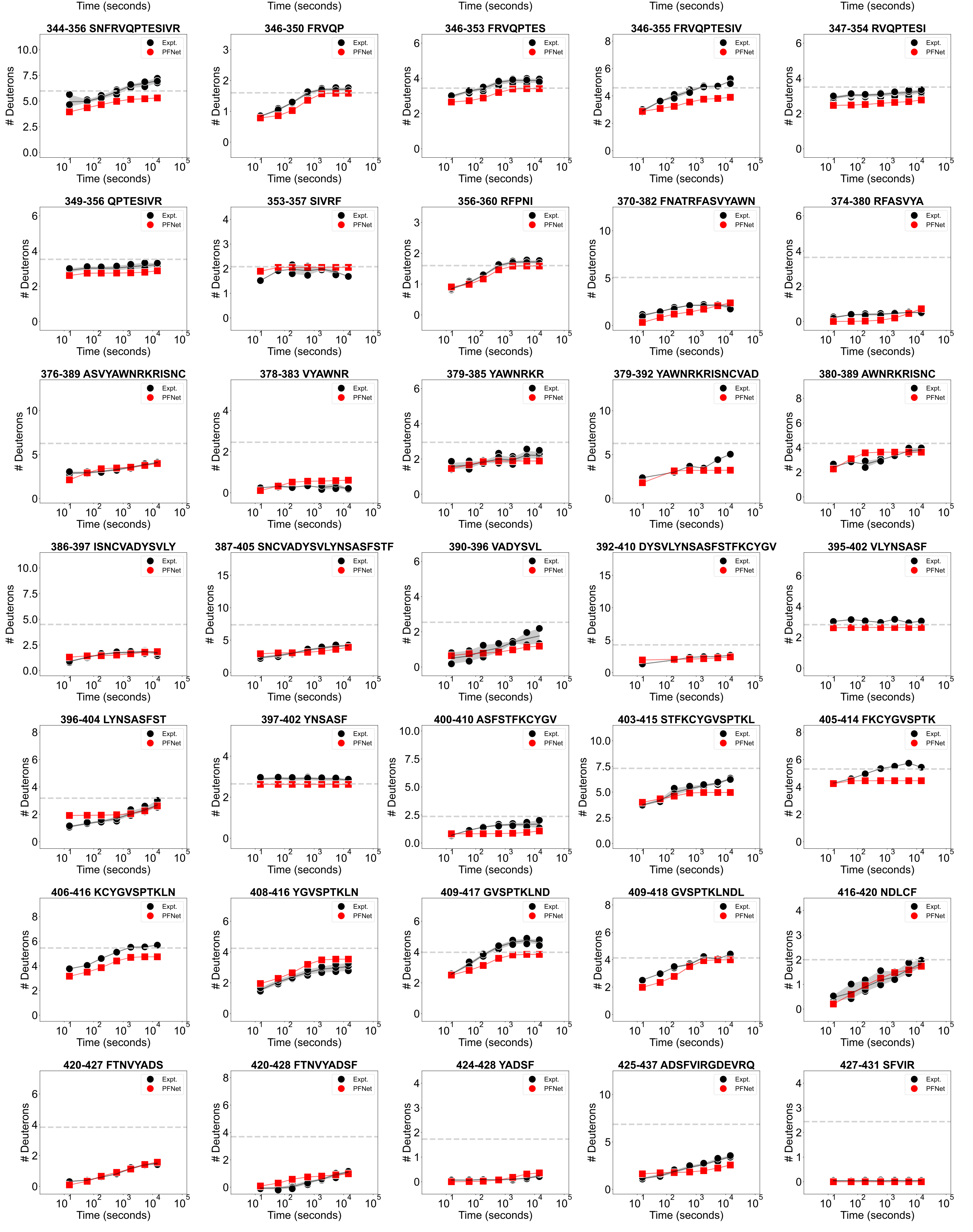

Time (seconds)
344-356 SNFRVQPTESIVR
346-350 FRVQP
346-353 FRVQPTES
346-355 FRVQPTESIV
347-354 RVQPTESI
349-356 QPTESIVR
353-357 SIVRF
356-360 RFPNI
370-382 FNATRFASVYAWN
374-380 RFASVYA
376-389 ASVYAWNRKRISNC
378-383 VYAWNR
379-385 YAWNRKR
379-392 YAWNRKRISNCVAD
380-389 AWNRKRISNC
386-397 ISNCVADYSVLY
387-405 SNCVADYSVLYNSASFSTF
390-396 VADYSVL
392-410 DYSVLYNSASFSTFKCYGV
395-402 VLYNSASF
396-404 LYNSASFST
397-402 YNSASF
400-410 ASFSTFKCYGV
403-415 STFKCYGVSPTKL
405-414 FKCYGVSPTK
406-416 KCYGVSPTKLN
408-416 YGVSPTKLN
409-417 GVSPTKLND
409-418 GVSPTKLNDL
416-420 NDLCF
420-427 FTNVYADS
420-428 FTNVYADSF
424-428 YADSF
425-437 ADSFVIRGDEVRQ
427-431 SFVIR
Expt.
PFNet
Deuterons

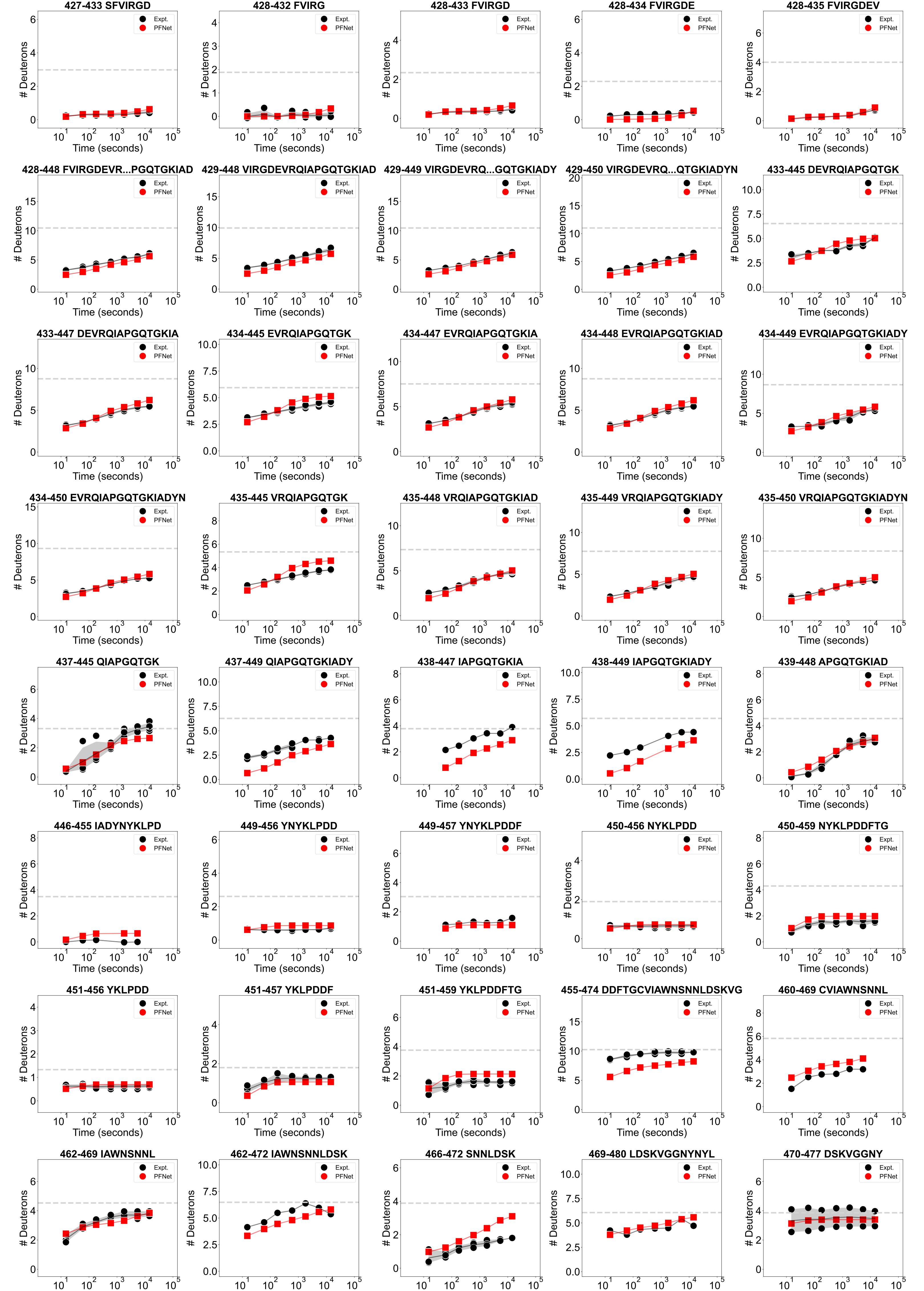
427-433 SFVIRGD
428-432 FVIRG
428-433 FVIRGD
428-434 FVIRGDE
428-435 FVIRGDEV
428-448 FVIRGDEVR...PGQTGKIAD
429-448 VIRGDEVRQIAPGQTGKIAD
429-449 VIRGDEVRQ...GQTGKIADY
429-450 VIRGDEVRQ...QTGKIADYN
433-445 DEVRQIAPGQTGK
433-447 DEVRQIAPGQTGKIA
434-445 EVRQIAPGQTGK
434-447 EVRQIAPGQTGKIA
434-448 EVRQIAPGQTGKIAD
434-449 EVRQIAPGQTGKIADY
434-450 EVRQIAPGQTGKIADYN
435-445 VRQIAPGQTGK
435-448 VRQIAPGQTGKIAD
435-449 VRQIAPGQTGKIADY
435-450 VRQIAPGQTGKIADYN
437-445 QIAPGQTGK
437-449 QIAPGQTGKIADY
438-447 IAPGQTGKIA
438-449 IAPGQTGKIADY
439-448 APGQTGKIAD
446-455 IADYNYKLPD
449-456 YNYKLPDD
449-457 YNYKLPDDF
450-456 NYKLPDD
450-459 NYKLPDDFTG
451-456 YKLPDD
451-457 YKLPDDF
451-459 YKLPDDFTG
455-474 DDFTGCVIAWNSNNLDSKVG
460-469 CVIAWNSNNL
462-469 IAWNSNNL
462-472 IAWNSNNLDSK
466-472 SNNLDSK
469-480 LDSKVGGNYNYL
470-477 DSKVGGNY
Deuterons
Time (seconds)
Expt.
PFNet

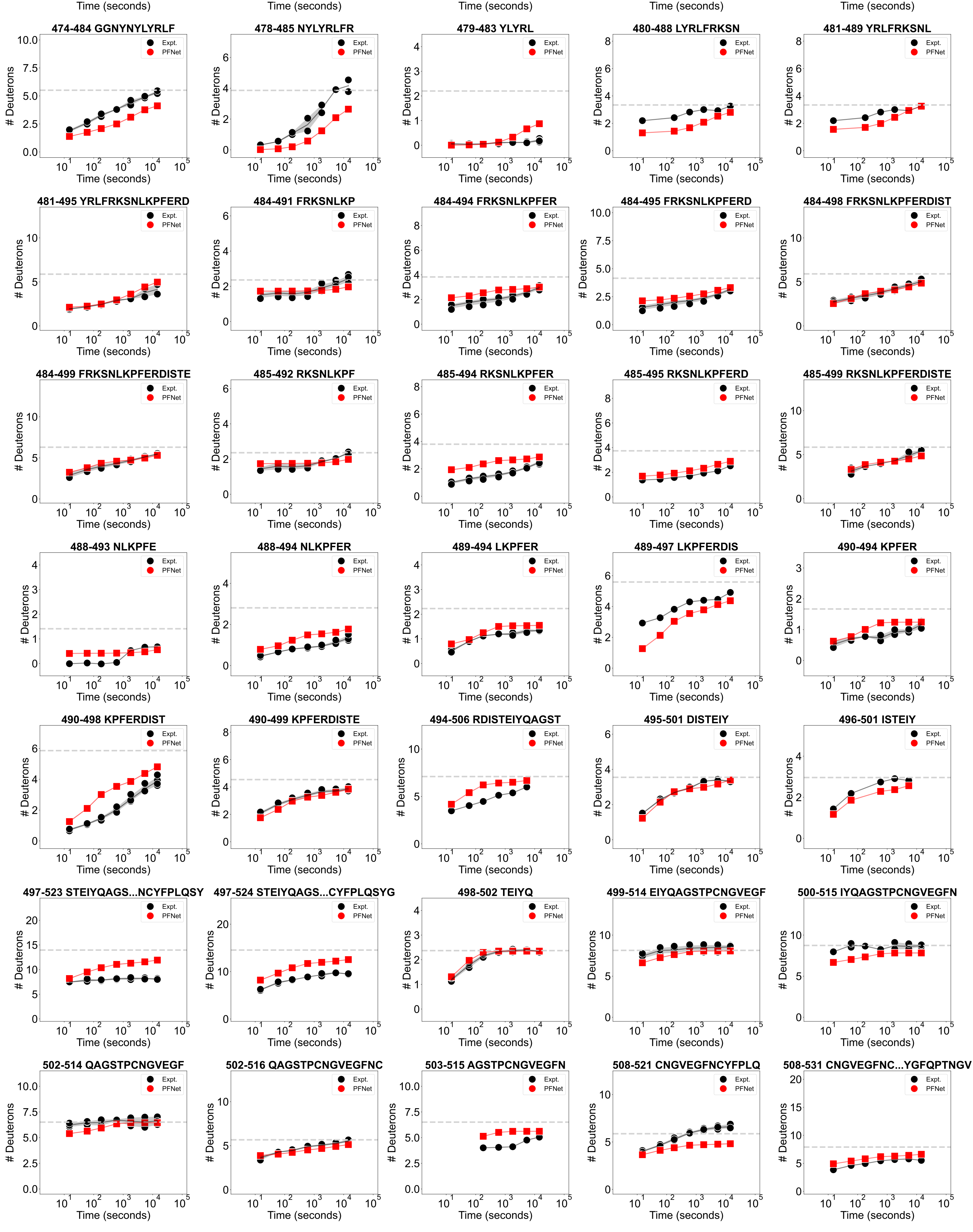
Time (seconds)
474-484 GGNYNYLYRLF
478-485 NYLYRLFR
479-483 YLYRL
480-488 LYRLFRKSN
481-489 YRLFRKSNL
481-495 YRLFRKSNLKPFERD
484-491 FRKSNLKP
484-494 FRKSNLKPFER
484-495 FRKSNLKPFERD
484-498 FRKSNLKPFERDIST
484-499 FRKSNLKPFERDISTE
485-492 RKSNLKPF
485-494 RKSNLKPFER
485-495 RKSNLKPFERD
485-499 RKSNLKPFERDISTE
488-493 NLKPFE
488-494 NLKPFER
489-494 LKPFER
489-497 LKPFERDIS
490-494 KPFER
490-498 KPFERDIST
490-499 KPFERDISTE
494-506 RDISTEIYQAGST
495-501 DISTEIY
496-501 ISTEIY
497-523 STEIYQAGS...NCYFPLQSY
497-524 STEIYQAGS...CYFPLQSYG
498-502 TEIYQ
499-514 EIYQAGSTPCNGVEGF
500-515 IYQAGSTPCNGVEGFN
502-514 QAGSTPCNGVEGF
502-516 QAGSTPCNGVEGFNC
503-515 AGSTPCNGVEGFN
508-521 CNGVEGFNCYFPLQ
508-531 CNGVEGFNC...YGFQPTNGV
Expt.
PFNet
Deuterons
Time (seconds)

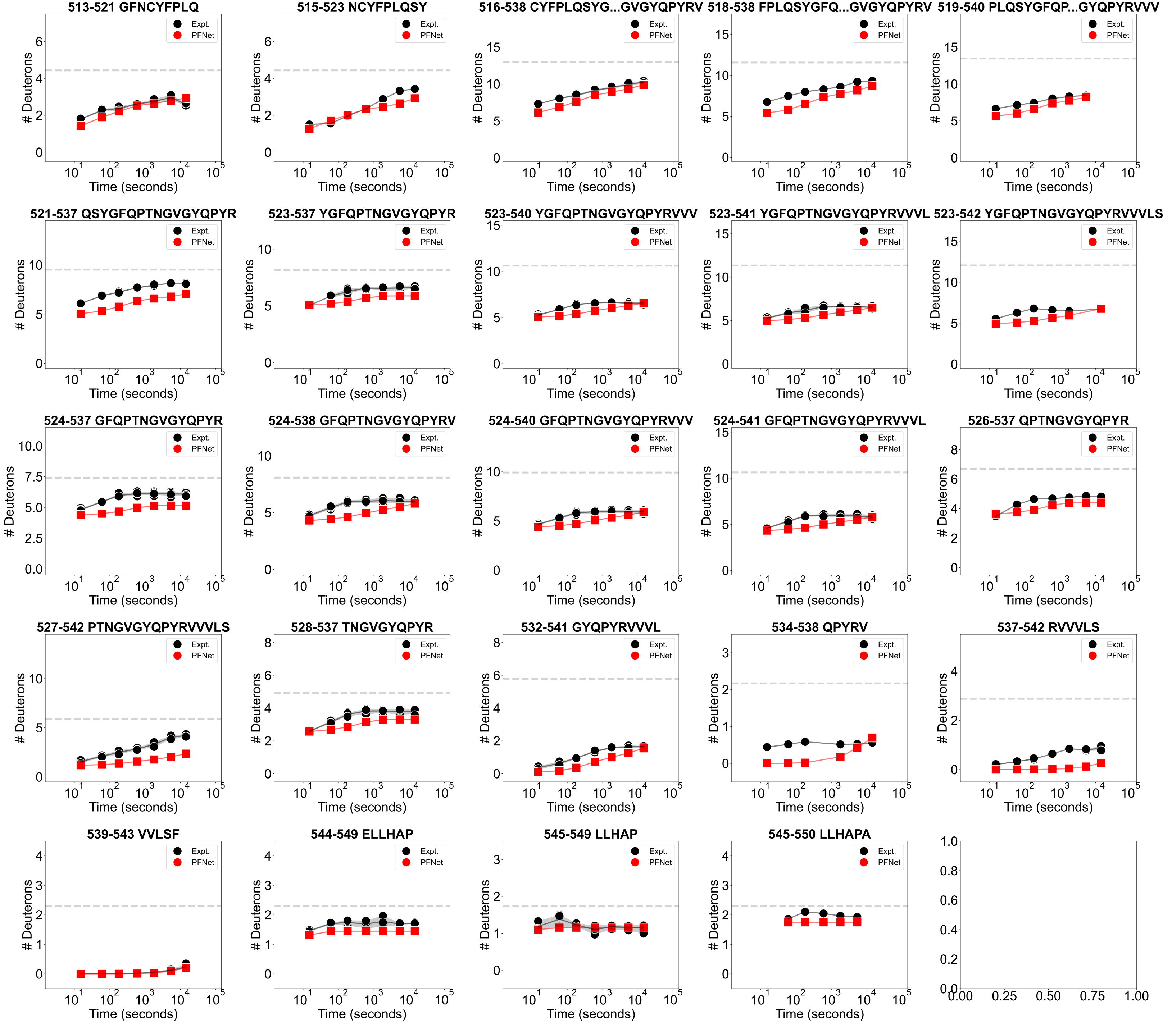
513-521 GFNCYFPLQ
515-523 NCYFPLQSY
516-538 CYFPLQSYG...GVGYQPYRV
518-538 FPLQSYGFQ...GVGYQPYRV
519-540 PLQSYGFQP...GYQPYRVVV
521-537 QSYGFQPTNGVGYQPYR
523-537 YGFQPTNGVGYQPYR
523-540 YGFQPTNGVGYQPYRVVV
523-541 YGFQPTNGVGYQPYRVVVL
523-542 YGFQPTNGVGYQPYRVVVLS
524-537 GFQPTNGVGYQPYR
524-538 GFQPTNGVGYQPYRV
524-540 GFQPTNGVGYQPYRVVV
524-541 GFQPTNGVGYQPYRVVVL
526-537 QPTNGVGYQPYR
527-542 PTNGVGYQPYRVVVLS
528-537 TNGVGYQPYR
532-541 GYQPYRVVVL
534-538 QPYRV
537-542 RVVVLS
539-543 VVLSF
544-549 ELLHAP
545-549 LLHAP
545-550 LLHAPA
Expt.
PFNet
Deuterons
Time (seconds)
